# Systematic Determination of the Impact of Structural Edits on Accumulation into Mycobacteria

**DOI:** 10.1101/2025.01.17.633618

**Published:** 2025-01-18

**Authors:** Rachita Dash, Zichen Liu, Irene Lepori, Mahendra D. Chordia, Karl Ocius, Kadie Holsinger, Han Zhang, Ryan Kenyon, Wonpil Im, M. Sloan Siegrist, Marcos M. Pires

**Affiliations:** 1 Department of Chemistry, University of Virginia, Charlottesville, VA 22904, USA; 2 Department of Microbiology, Immunology, and Cancer, University of Virginia, Charlottesville, VA 22904, USA; 3 Department of Microbiology, University of Massachusetts, Amherst, MA USA; 4 Department of Chemistry, Lehigh University, Bethlehem, PA 18015, USA; 5 Departments of Biological Sciences and Bioengineering, Lehigh University, Bethlehem, PA 18015, USA

## Abstract

The mycomembrane of mycobacteria has long been regarded as the primary barrier to the accumulation of molecules within these bacteria. Understanding accumulation beyond the mycomembrane of *Mycobacterium tuberculosis* (*Mtb*) is crucial for developing effective antimycobacterial agents. This study investigates two design principles commonly found in natural products and mammalian cell-permeable peptides – backbone *N*-methylation and macrocyclization – aimed at enhancing accumulation. To assess the impact of these structural edits on accumulation, we employed our recently described Peptidoglycan Accessibility Click-Mediated Assessment (PAC-MAN) assay for live-cell analysis. Our findings indicate that peptide macrocyclization generally enhances permeability and metabolic stability, while *N*-methylation modifies accumulation in a context-dependent manner. Furthermore, we applied these design principles to the peptide antibiotic Tridecaptin A1, demonstrating improved permeability and efficacy against mycobacteria, in specific contexts. This work suggests that strategic structural modifications can enhance accumulation past the mycomembrane and provide the first systematic governing rules for the rational redesign of potential antimycobacterial agents. Most importantly, our results broadly challenge the notion that large hydrophilic molecules (*e.g*., peptides) cannot readily bypass the mycomembrane to reach the cytosolic space.

## MAIN

Tuberculosis (TB) is a critical and major global public health concern, with over 10 million cases reported worldwide in 2022.^1^ Often recognized as the deadliest infectious disease, TB is currently exhibiting a surge in incidence, reversing a ten-year trend of decline.^2^ The causative agent of TB is the bacterium *Mycobacterium tuberculosis* (*Mtb*). *Mtb* and other mycobacteria feature a complex cellular envelope comprising of an outer mycomembrane layer, followed by an arabinogalactan layer, the peptidoglycan (PG) layer, and ultimately, the inner membrane.^3^ The mycomembrane is uniquely thick and hydrophobic in nature, as it primarily consists of long-chain lipids (mycolic acids) and glycolipids.^3^ The mycomembrane is also particularly dynamic and complex, highlighted by the fact that the mycolic acids in the outer leaflet can exist either freely or linked to trehalose moieties.^4^ Further, the mycolic acids in the inner leaflet are covalently attached to the underlying arabinogalactan layer.^4^ Importantly, the mycomembrane is widely considered to be the primary permeation barrier for the entry of antibiotics and endow mycobacteria with a high level of intrinsic resistance.^4–7^

Most antibiotics currently used in the treatment of tuberculosis, such as isoniazid, rifampicin, ethambutol, and pyrazinamide, are small, hydrophobic molecules that were developed several decades ago.^8^ These molecular features of efficacious TB antibiotics have been purported to be a direct consequence of the difficult-to-cross mycomembrane barrier. The overuse of these therapeutics, compounded by suboptimal patient adherence due to the prolonged treatment regimens, has led to the emergence of multi-drug resistant (MDR) and total drug-resistant (XDR) strains of *Mtb*.^9^ In recent years, only two new antibiotics (also small and hydrophobic), bedaquiline and pretomanid, have been introduced for the treatment of drug-resistant tuberculosis, emphasizing the urgent necessity to enhance the current pre-clinical antimycobacterial pipeline.^10^

**Figure 1.**
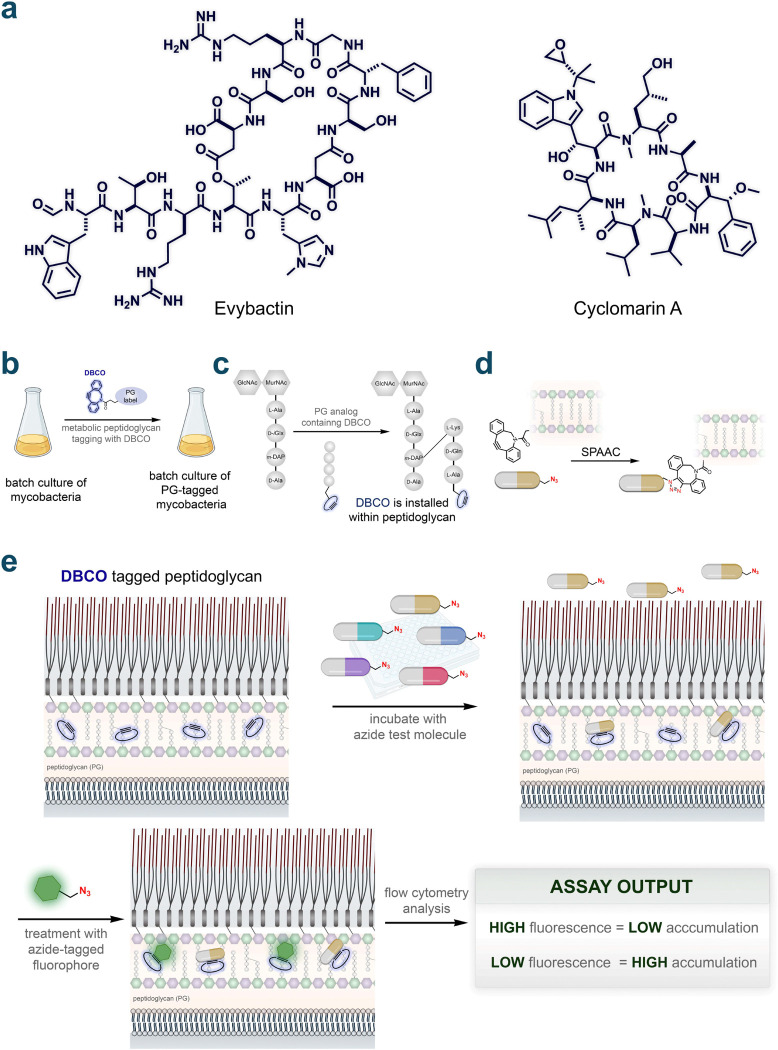
Structures of example antimicrobial peptides and assay schemes for determination of accumulation for azide test molecules. **a**, Chemical structure of evybactin and cyclomarin A. **b**, General schematic for DBCO modification of mycobacteria. Mycobacteria were grown in batches and labeled metabolically using a DBCO-tagged peptidoglycan probe. **c**, Schematic showing the installation of DBCO within peptidoglycan. The PG analog containing DBCO on the N-terminus of a tetra peptide is installed on the stem peptide in peptidoglycan with covalent link between the meso-α,ε-Diaminopimelic acid residue (m-DAP) on the stem peptide and Lys residue on the PG analog. **d**, Schematic illustration of strain-promoted azide-alkyne cycloaddition (SPAAC) between the azide test molecules and the installed DBCO within peptidoglycan. **e**, Schematic illustration of biological assay in this work determining the accumulation of azide test molecules within the periplasmic space of mycobacteria. Mycobacteria with DBCO tagged peptidoglycan are incubated with azide test molecules which will form a covalent link with the DBCO once accumulated in the peptidoglycan. The bacteria cells are then treated with an azide-tagged fluorophore, which will attach to the unoccupied DBCO in the peptidoglycan. Cellular fluorescent intensities are measured by flow cytometry where hits with high fluorescence intensity are interpreted as low accumulation and vice versa.

There are notable exceptions (e.g., rifampicin) to the size constraint purported to operate in TB antibiotics. Peptidic molecules that are well beyond Lipinski’s Rule of Five (bRo5)^11^ have attracted interest in drug design because of their ability to bind targets with greater specificity and affinity. Broadly, peptide drugs have recently drawn considerable interest^12,13^ across disease areas including oncology^14^ and metabolic^15,16^ disorders. A noteworthy antibacterial example is the development of an entirely new class of macrocyclic peptide antibiotics (Zosurabalpin developed by Roche) that specifically target the lipopolysaccharide transporter in carbapenem-resistant *Acinetobacter baumannii*.^17^ Additionally, peptide candidates are also being actively developed for mycobacterial infections.^18,19^ This growth in interest is highlighted by the recent discovery of evybactin^20^ and cyclomarin A^21^ (Fig. 1a), which was also the basis of the promising series of BacPROTACs.^22–25^

Backbone *N*-alkylation and the macrocyclization of peptides have been extensively studied for the enhancement of passive membrane permeability across mammalian cells.^26–30^ These modifications are frequently found in nature, as evidenced by the abundance of natural *N*-methylated macrocyclic peptide products.^31–35^ However, despite some indication that *N*-methylated and macrocyclic peptides may be privileged in their ability to permeate across mammalian bilayers, these strategies have not yet been empirically validated in mycobacteria. A challenge in the field is the general lack of tools to readily measure the penetration of molecules past the mycomembrane. In our previous work, we developed the Peptidoglycan Accessibility Click-Mediated Assessment (PAC-MAN) assay for live-cell analysis, and demonstrated the assessment of the accumulation of small molecules across the mycomembrane.^36^ Herein, we expand the work to the systematic evaluation of two structural alterations in peptides that could potentially enhance molecular accumulation past the mycomembrane: backbone *N*-alkylation and macrocyclization. Through a comprehensive series of peptides, we demonstrated that specific structural features significantly influence accumulation levels in mycobacteria. This discovery lays the groundwork for a set of prescriptive modifications that can be employed in developing more effective antibiotics targeting mycobacteria. By applying these strategies to a poorly permeable antibiotic, we observed that, in certain cases, these structural modifications can substantially enhance antibiotic activity against mycobacteria.

## RESULTS

### Establishing PAC-MAN Assay Parameters

The measurement of the accumulation of molecules in bacteria has proven to be technically challenging. Previously, liquid chromatography-tandem mass spectrometry (LC-MS/MS) has been used to measure whole cell associated molecule concentrations in bacteria.^37,38^ Several key aspects of this method present significant advantages for measuring the uptake of molecules in bacteria, including the elimination of the requirement for a chemical tag. But there are also two principal limitations in its current application: (a) limited throughput capacity and (b) a lack of precision in pinpointing the exact location of molecules, as it typically measures ‘whole-cell’ colocalization or association. Despite these challenges, recent efforts by the Hergenrother laboratory have successfully utilized LC-MS/MS to develop the eNTRy rules in *Escherichia coli* and *Pseudomonas aeruginosa*.^39,40^ In contrast, no large scale LC-MS/MS analysis has been described in mycobacteria; the largest screen performed prior to our development of PAC-MAN was eleven small molecules in total.^41^ Critically, PAC-MAN registers the arrival of a compound past the mycomembrane once it has reached the PG layer in the periplasmic space and not whole cell association.

We previously demonstrated that the PG layer of live mycobacteria can be metabolically labeled by treating live cells with fluorophore-tagged analogs of the stem peptide of the PG.^42–44^ In PAC-MAN, the fluorophore is replaced with a strained alkyne dibenzocyclooctyne (DBCO) unit. Similarly, treating mycobacterial cells with this DBCO-tagged cell wall analog enables the site-specific tagging of the PG layer (Fig. 1b). More specifically, cell wall linked transpeptidases crosslink the exogenous stem peptide onto the existing PG framework, thereby establishing a landmark past the mycomembrane (Fig. 1c). Incubation of azido-tagged molecules with the DBCO-labeled mycobacteria cells then allows the register of their arrival past the mycomembrane onto the PG through strain-promoted alkyne-azide cycloaddition (SPAAC) (Fig. 1d). A subsequent incubation step with an azido-tagged fluorophore in a secondary SPAAC reveals the unoccupied DBCO landmarks. As a result, cellular fluorescence is inversely proportional to the level of molecule accumulation (Fig. 1e). The robust and straightforward nature of these steps removes two critical bottlenecks in accumulation measurements: hundreds of measurements can be performed in a single experiment and there is confirmation of the arrival of the molecule past the most critical barrier of entry.

**Figure 2.**
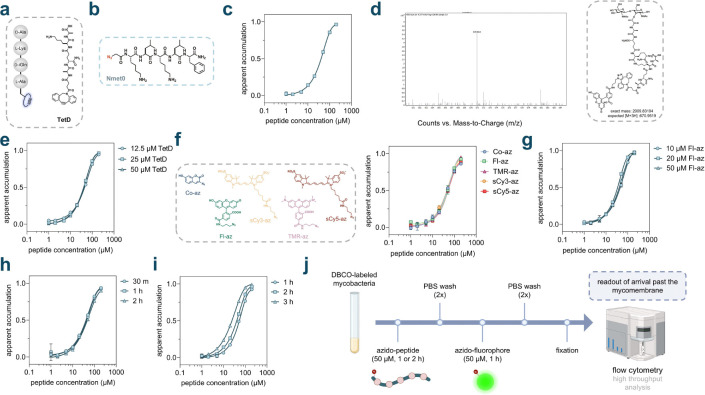
Optimization of assay conditions. Mycobacterial cells were incubated at 37 °C without being mentioned in all experiments. Fluorescence intensities measured from flow cytometry were normalized to the negative control in each experiment (data not shown). Apparent accumulations are calculated as the subtraction of normalized fluorescence intensities from 1. Data points and error bars represent the average and s.d. from three technical repeats, respectively. **a**, Schematic illustration and chemical structure of **TetD**. **b**, Chemical structure of model peptide **Nmet0**. The azide group is tagged on the N-terminus of the peptide through a-azido acetamide. **c**, Concentration dependent accumulation of **Nmet0**. Peptides were incubated with DBCO labeled mycobacterial cells for 1 h at 37 °C, then treated with 50 mM **Fl-az** for 1 h. **d**, Mass spectrometry analysis of peptidoglycan composition containing fluorescent labeled muropeptides conjugated with **TetD**. Bacterial cells were labeled with 25 mM **TetD** for 18 h and treated with 50 mM **Fl-az** for 1 h after harvest. The peptidoglycan components from treated cells were isolated and digested using lysozyme and mutanolysin as described in the supporting information. Digested peptidoglycan samples were filtered through 10kDa MWCO membrane and subjected for analysis by LC-MS. **e**, Concentration dependent accumulation of **Nmet0** with bacteria cells treated with different concentrations of **TetD**. Mid-log phase mycobacteria mc^2^ 155 were incubated with different amounts of **TetD** for 18 h and then tested for concentration dependent accumulation of **Nmet0**. **f**, Concentration dependent accumulation of **Nmet0** tested using different azido-fluorophores across the fluorescence spectrum. **Co-az**, 7-hydroxy-3-azido coumarin. **Fl-az**, 6-azido fluorescein. **sCy3-az**, azido-sulfo-Cy3. **TMR-az**, 5-azido tetramethyl rhodamine. **sCy5-az**, azido-sulfo-Cy5. Mycobacterial cells were treated with 50 mM different azido fluorophores for 1 h after incubation with different concentrations of **Nmet0**. **g**, Concentration dependent accumulation of **Nmet0** using different concentrations of **Fl-az**. Mycobacterial cells were treated with indicated concentrations of **Fl-az** for 1 h after incubation with different concentrations of **Nmet0**. **h**, Concentration dependent accumulation of **Nmet0** using different treatment lengths of **Fl-az**. Mycobacterial cells were treated with 50 mM **Fl-az** for different lengths of time after incubation with different concentrations of **Nmet0**. **i**, Concentration dependent accumulation of **Nmet0** using different incubation lengths. Harvested DBCO labeled mycobacterial cells were incubated with different concentrations of **Nmet0** for 30 min, 1 h, or 2 h, then treated with 50 mM **Fl-az** for 1 h. **j**, Schematic illustration of optimized assay protocol. For the rest of experiments, harvested DBCO-labeled mycobacteria were incubated with 50 mM azido-peptides for 1 h or 2 h, washed with PBST (phosphate buffered saline with 0.05% tween80) twice, then treated with 50 mM azido-fluorophore for 1 h, washed with PBST twice and fixed in 4% formaldehyde in PBS before high throughput analysis on flow cytometry.

At first, we aimed to build on our prior assay parameters by conducting initial experiments in *Mycobacterium smegmatis* (*Msm*), a model organism that recapitulates key features of the pathogenic *Mycobacterium tuberculosis* (*Mtb*).^45^ Live cell PG labeling was initiated by incubating *Msm* cells with **TetD**, which is the stem peptide analog featuring DBCO on the *N*-terminus (Fig. 2a). Two peptides were assembled to serve as the baseline members of a backbone methylated (**Nmet0**) library (Fig. 2b) and linear (**Lin0**) library (Fig. S1). For each peptide, a phenylalanine residue was included to obtain definitive concentration levels based on the chromophore, a lysine residue to impart greater water solubility, and an azido tag for quantification. To initially test the baseline molecule **Nmet0** *via* PAC-MAN, **TetD** labeled cells were harvested and subsequently incubated with varying concentrations of the peptide. This pulse step was followed by chase treatment with an azide-tagged fluorophore, **Fl-az** and analyzed *via* flow cytometry. Notably, to facilitate clearer interpretation, the data were processed as 1−(normalized fluorescence intensity), establishing a positive correlation between the Y-axis values and observed accumulation, represented as ‘apparent accumulation’. A high apparent accumulation value indicates that the molecule exhibits relatively better accumulation through the mycomembrane and greater accumulation in the periplasm. There is a clear concentration-dependent response that indicates peptide **Nmet0** is arriving within the periplasmic space of *Msm* cells (Fig. 2c). This finding was rather surprising, considering the physiochemical properties of the molecules – generally small and greasy – that were believed to enhance permeation across the mycomembrane of mycobacteria.

A series of subsequent experiments were performed to establish key aspects of PAC-MAN. To confirm the installation of DBCO within the PG scaffold, sacculi was isolated from **TetD** treated cells that were subsequently treated with the dye **Fl-az**. Isolation was performed using standard procedures (familiar to our labs) by enzymatically depolymerizing the sacculi with mutanolysin and lysozyme.^36^ Fragments were analyzed by high-resolution Q-TOF mass spectrometry and revealed a muropeptide fragment containing the click chemistry product (Fig. 2d). We next turned our attention to testing whether the concentration of **TetD** during the labeling step could alter the total levels of DBCO within the PG layer, as we had previously found.^36^ As expected, increasing concentrations of **TetD** led to higher levels of cellular fluorescence (Fig. S2). Despite observing distinct absolute fluorescence intensities with varying DBCO amounts, the normalized EC_50_ values were nearly identical across the tested concentration ranges (Fig. 2e). This indicates that upon normalization, the accumulation profiles are broadly unaffected by the absolute amount of DBCO landmarks with the PG scaffold. From these results, we set 25 μM of **TetD** as the label concentration for the PG labelling for all subsequent assays. Importantly, the cell morphology or cell envelope integrity did not change in cells treated with **TetD** at this concentration, as evidenced by microscopy (Fig. S3) and ethidium bromide (EtBr) accumulation/efflux experiments (Fig. S4), respectively. Notably, EtBr is used as an indicator of mycomembrane integrity, which, when disrupted, leads to increased intracellular accumulation of EtBr and subsequently heightened cellular fluorescence.^46–50^

Next, we aimed to test the tolerance of PAC-MAN across a range of azide-tagged fluorophores. In addition to **Fl-az**, we tested 3-azido-7-hydroxy coumarin (**Co-az**), azido-tetramethyl rhodamine (**TMR-az**), azido-sulfo Cy3 (**sCy3-az**) and azido-sulfo Cy5 (**sCy5-az**) with *Msm* cells (Fig. 2f). Our results showed that cellular fluorescence signals for cells treated with **Fl-az**, **sCy3-az** or **sCy5-az** had comparable signal-to-noise ratios and thus we opted for **Fl-az** for further experiments. To test if the concentration of **FI-az** during the chase step could impact the results observed, we set up an experiment in which **TetD** treated *Msm* cells were incubated with three concentrations of **Fl-az**. Our results showed that cellular response in PAC-MAN using **Nmet0** were nearly identical across all **FI-az** concentrations tested (Fig. 2g), indicating that even at the lowest concentration there was exhaustive installation of the fluorophore. Similarly, we also observed nearly identical cellular response upon testing different incubation times with **FI-az** (Fig. 2h). The incubation time with the test molecule in the pulse step was evaluated next by incubating molecules across three incubation periods (Fig. 2i). Longer incubation times shifted the curve accordingly, a finding that is expected given the nature of the detection upon periplasmic arrival and similar to mammalian Chloroalkane Penetration Assay (CAPA).^51^ We note the second base peptide (**Lin0**) was subjected to similar assay development conditions that mirrored much of the steps involving **Nmet0** and the results were similar (Fig. S5). Together, these conditions established the workflow for the analysis of test peptides in PAC-MAN (Fig. 2j).

### Effect of Backbone *N*-alkylation on Peptide Accumulation

**Figure 3.**
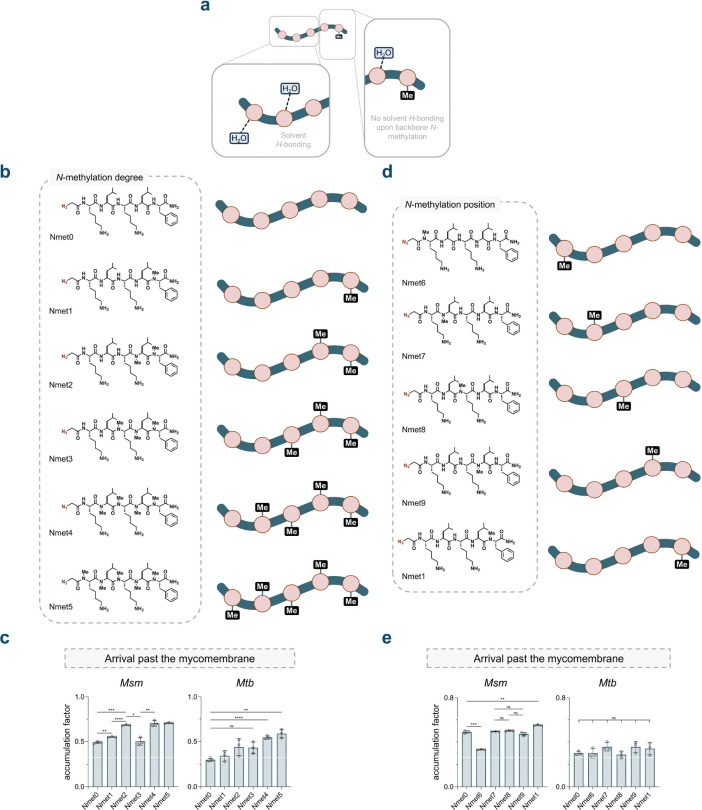
Design of *N*-methyl peptide library and accumulation of the peptides in *Mycobacterium smegmatis* (Msm) and *Mycobacterium tuberculosis* (Mtb). **a**, Schematic illustration of peptide interacting with solvent molecules in aqueous solution. Hydrogen bonds between the amide backbone and water molecules are shown by dashed links. Installation of a methyl group on the backbone amide eliminates the correlated hydrogen bonding for the specific amide. **b**, **d**, Schematic illustration and chemical structure of *N*-methyl peptide library. **c**, **e**, Accumulation of peptides tested in Msm and Mtb. **b**, Increasing degrees of methylation on the peptide backbone from the residues on the C terminus to the N terminus from 1 to 5. **c**, Accumulation of peptides tested in Msm and Mtb with different degrees of *N*-methylation. **d**, Systematic design of mono-methylated peptides with methylation on different backbone amide positions from each amino acid residue. **e**, Accumulation of peptides tested in Msm and Mtb with mono *N*-methylation in different positions. DBCO-labeled mycobacterial cells were incubated with 50 mM of each peptide for 1 h, then treated with 50 mM **Fl-az** for 1 h. Fluorescence intensities were measured by flow cytometry and normalized to the negative control in each experiment (data not shown). Accumulation factors are calculated as the subtraction of normalized intensities from 1. Data points and error bars represent the average and s.d. from three technical repeats, respectively. ns, no significance. *p < 0.1, **p < 0.01, ***p < 0.001, ****p<0.0001.

Methylation of the amide backbone in peptides and peptidic natural products has been previously evaluated for its ability to modulate membrane permeability in mammalian systems.^35,52–55^ For the permeability effect, this structural edit is generally considered to alter the molecule’s hydrogen bonding network with surrounding water, which can reduce the overall hydrophilicity of the molecule and promote its desolvation prior to passive diffusion across the membrane (Fig. 3a). To investigate this feature using PAC-MAN, we constructed libraries of *N*-methylated peptides derived from the base peptide **Nmet0**, which served as the unmethylated control. In the first series, peptides **Nmet1** to **Nmet5** had a variable total number of methylation marks within the peptidic backbone (Fig. 3b). Our data showed a significant increase in apparent accumulation upon a single methylation mark at the C-terminal end of the peptide in **Nmet1**. This increase was further pronounced upon the addition of a second methylation for **Nmet2**. Interestingly, the trimethylated molecule, **Nmet3**, deviated from this trend (Fig. 3c). Notably, previous accounts^56,57^ have suggested that having high levels of *N*-methyl groups may not necessarily correlate with optimum permeability in mammalian systems, potentially due to conformational reasons. In *Mtb*, the trend was markedly different than *Msm* (Fig. 3c). While minimal changes were observed in the first three additions of methylations, the last two methylations indeed led to increases in apparent accumulation.

In the second subseries (**Nmet6** to **Nmet9**), a single methylation was installed in a systematic walk across the peptidic backbone of **Nmet0** (Fig. 3d). In *Msm*, our results showed a surprising decrease in apparent accumulation upon *N*-terminal methylation of **Nmet6** compared to its unmethylated counterpart **Nmet0** (Fig. 3e). Similar apparent accumulation to **Nmet0** was observed when the methylation was present on the central backbone amides, whereas a slight increase in accumulation compared to **Nmet0** was observed when the methylation mark was moved to the *N*-terminal end (Fig. 3e). However, this effect was mitigated in *Mtb* as there was no significant accumulation difference within the mono-methylation series (Fig. 3e). Eric Biron et al. and Oded Ovadia et al. had reports on the differences shown on small cyclic peptides tested with Caco-2 monolayer and liposome model.^58,59^ R. Scott Lokey et al. had also tested a similar concept in mammalian systems using PAMPA and found interesting positional and conformational effects in accumulation.^56,57^ These backbone methylation data suggest an overall trend that backbone *N*-methylation can modulate peptide accumulation across the mycomembrane, with the caveat that the optimal amount may be location and species specific.

Next, we wanted to test if there were reactivity differences within our series of azido-tagged molecules with respect to their engagement with the DBCO landmarks installed during the **TetD** cellular treatment. Previous work from our group investigated the reactivity of azido-tagged small molecules using a bead-based assay.^36^ Briefly, we employed amine-terminated beads that were modified to include terminal DBCO handles, which are also compatible with flow cytometry analyses. This approach mirrors our live cell analysis (pulse with azide-tagged test molecules and chase with azide-tagged fluorophore) but the engagement of the azido group with the DBCO lacks the mycomembrane barrier. In the absence of DBCO modification to the beads, background fluorescence levels were observed upon the bead treatment with the azide tagged fluorophore, **Fl-az** (Fig. S6). Our results showed that using the same concentration of peptides as our PAC-MAN assay, all molecules equally and fully reacted with the beads in the absence of the mycomembrane (Fig. S6). These results strongly suggest that the bead assay can support the analysis of the engagement of the peptides with the DBCO landmarks in the absence of a membrane barrier, and, most critically, that our assay indeed reports on accumulation of the molecules across the mycomembrane. We also set out to investigate the integrity of the critical mycobacterial membrane upon metabolic labeling by **TetD** and subsequent treatment with our library using EtBr accumulation/efflux measurements. No significant differences were detected in the accumulation of EtBr between treatments with different molecules and the vehicle (Fig. S7). These findings suggest that the mycomembrane is fully intact and that the anchoring onto the DBCO landmarks should provide a route to test the accumulation of modified peptides past this critical barrier.

We next constructed a peptoid library with the same sequence as the *N*-methylation library, **Nmet0**. The goal was to consider the contribution of the backbone amides in a different but biologically relevant context. Similar to *N*-methylated peptides, peptoids are devoid of the hydrogen bond donor capacity of the backbone amide, ^60–65^ and, therefore, could potentially possess higher levels of accumulation relative to their peptide counterparts. To systematically sample the different numbers and patterns of backbone *N*-alkylation, **Nalk1** to **Nalk5** were assembled in which the *N* to the *C*-terminus had an increasing number of *N*-substituted glycine units (Fig. S8a). To complement the series, we synthesized **Nalk5** to **Nalk9**, which possessed mono-*N*-substituted glycine units across various positions within the peptidic backbone (Fig. S8b). The peptoid library was then tested with live cell PAC-MAN to determine the accumulation profile for each member of library using the same conditions as the *N*-methylation library (Fig. S8c). Unlike our results for **Nmet1-Nmet9**, our data showed that there was a much greater impact and variability in how the shift from the α-carbon to the backbone amide impacted accumulation. An unexpected decrease in apparent accumulation was observed for the *N*-terminal alkylated peptoid **Nalk1** compared to the base peptide **Nmet0**, similar to the *N*-methylation library (Fig. S8c). Upon increasing number of *N*-alkylation being applied from *C*-terminus to the *N*-terminus, an increase in the apparent permeability of the peptides was observed with the potential maximum reached after having three *N*-substituted glycine units. However, an apparent drop in the accumulation profile was observed for the fully substituted peptoid **Nalk5**. For the mono-*N*-substituted glycine series, while similar apparent accumulation was observed for the *N*-alkylated residue moving its position in the three centered amino acids, a slight increase in their accumulation can be observed. However, contrary to the *N*-methylation library, switching the *C*-terminal phenylalanine residue to an *N*-alkylated glycine unit showed an adverse effect on its apparent accumulation (Fig. S8c). As before, we observed that the peptoid library readily reacted with our **TetD** labelled beads (Fig. S9) and the cell integrity remained unaffected based on EtBr accumulation experiments (Fig. S10). Overall, the data above suggested that backbone alkylation in peptoid like molecules lead to higher permeability across the mycomembrane for the tested peptides.

### Effect of Macrocyclization on Permeability

**Figure 4.**
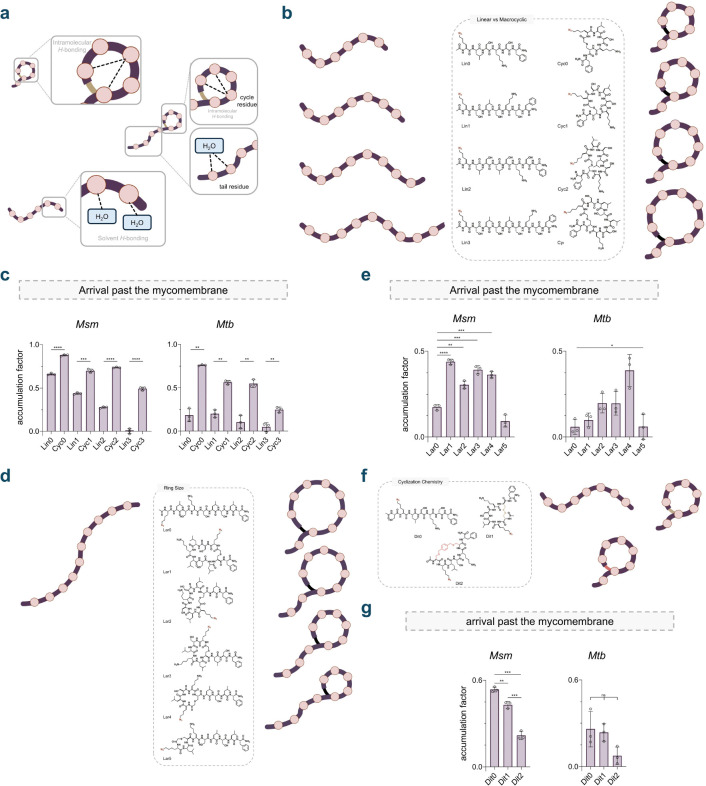
(a) Schematic illustration of macrocyclization of peptides promoting intramolecular hydrogen-bonding. Intramolecular hydrogen-bonding subsequently reduces hydrogen-bonding with the solvent, thereby reducing membrane permeation associated desolvation penalty. (b) Chemical structures of the linear vs macrocyclic sub-library members containing macrocyclic peptides of increasing sizes (Cyc0-Cyc3) and their linear counterparts (**Lin0-Lin3**). **(c)** Apparent accumulation of the linear vs macrocyclic sub-library across the mycomembrane in *Msm* and *Mtb* after 2 h of treatment with 50 μM compound. **(d)** Chemical structures of the ring size sub-library members containing macrocyclic peptides of increasing ring sizes (Lar1-Lar5) and their linear counterpart (**Lar0**). **(e)** Apparent accumulation of the ring size sub-library across the mycomembrane in *Msm* and *Mtb* after 2 h of treatment with 50 μM compound. **(f)** Chemical structures of the cyclization chemistry sub-library members containing a disulfide bonded macrocyclic peptide (**Dit1**), a bis-electrophilic linker based macrocyclic peptide (**Dit2**) and their linear counterpart **Dit0**. **(g)** Apparent accumulation of the cyclization chemistry sub-library across the mycomembrane in *Msm* and *Mtb* after 2 h of treatment with 50 μM compound. Accumulation factors are calculated as the subtraction of normalized intensities from 1. Data points and error bars represent the average and s.d. from three technical repeats, respectively. ns, no significance. *p < 0.1, **p < 0.01, ***p < 0.001, ****p<0.0001.

Cyclization has been widely explored to improve the pharmacokinetic properties of large molecules such as peptides.^66,67^ Cyclic molecules may present a more compact and favorable shape, aiding in the ability to penetrate membrane barriers more easily compared to their acyclic counterparts, which might be more extended or linear.^27,33,68^ The cyclic conformation may promote intramolecular hydrogen bonding and/or also reduce the exposure of polar functional groups to the surrounding environment, decreasing their solubility in water and promoting membrane integration (Fig. 4a). For the assembly of our macrocyclic molecule library, a thioether-based cyclization strategy was chosen given its metabolic stability and facile formation under mild conditions,^69^ providing a reliable chemical route to a range of macrocyclic peptides. Additionally, thioether bonded macrocyclic peptides are being extensively explored as potential drug scaffolds.^70–73^ Specifically, we envisioned a chloroacetyl group coupled to the *N*-terminus of the peptide during the solid phase peptide synthesis (SPPS) steps, and a cysteine residue incorporated into the peptide sequence (Fig. S11). This configuration would enable the formation of a thioether bond between the thiol group of the cysteine residue and the *N*-terminal chloroacetyl group (Fig. S11). Importantly, the *C*-terminal position was deliberately avoided for the cysteine residue due to concerns of complications with potential epimerization^74^ and β-piperidinylalanine formation^75^ during chain extension.

For each macrocyclic molecule, an appropriate linear control molecule was designed, preserving the sequence but replacing the cysteine residues with serine, and acetylating the *N*-terminus to preserve net charge. It is noteworthy that the reduced cysteine versions of the molecules were not considered as appropriate linear controls due to their susceptibility to rapid oxidation under the proposed assay conditions; this would potentially yield a number of variable and difficult to characterize molecules. Based on this macrocyclic library design, we synthesized three distinct sub-libraries using standard solid phase peptide synthesis. First, appreciating that the molecular size of our macrocyclic molecules can influence membrane permeability,^76,77^ we designed a set of macrocyclic molecules (**Cyc0-Cyc3**) and their corresponding linear controls of increasing lengths (**Lin0-Lin3**) (Fig. 4b). Size varied by modulating the number of leucine and serine residues. This sub-library was then tested using our PAC-MAN assay in live mycobacteria. We observed that, generally, for any given size we tested, the macrocyclic molecule demonstrated better permeability across the mycomembrane compared to its linear counterpart (Fig. 4c). Importantly, this pattern was also observed upon testing the library in *Mtb* (Fig. 4c). Additionally, in both organisms, we observed a general trend whereupon permeability diminished with increasing molecular size, across both linear and macrocyclic molecules (Fig. 4c). Notably, **Cyc0**, the shortest macrocyclic molecule in our panel, exhibits better accumulation than any other macrocyclic library member and is comparably permeable to the most permeable molecule we found in our previous work.^36^ Next, inspired by natural products, we then turned our attention towards lariat-shaped molecules. Lariat-shaped molecules have recently been recognized for their utility both as drug scaffolds^78^ as well as membrane permeators.^57^ Their flexible linear tails provide additional functional versatility that can be modulated to specifically target proteins of interest. We synthesized a series of molecules (**Lar1-Lar5**) cyclized from an *N*-chloroacetyl group onto a variably positioned cysteine side chain to generate a collection of lariat-shaped molecules (Fig. 4d). Notably, linear control **Lar0** was designed to not have any cysteine residues and was *N*-acetylated. Following the same trend as before, the macrocyclic lariat-shaped molecules **Lar1-Lar4** were found to be better accumulators than linear **Lar0** in *Msm* (Fig. 4e). Notably, **Lar5** demonstrated poor accumulation in *Msm*, which we attribute to the small size of its ring, leaving a significant portion of the peptide still flexible and more solvent exposed (Fig. 4e). Within the lariat-shaped macrocyclic series (**Lar1-Lar5**), no discernible trend was observed in both *Mtb* and *Msm*. **Lar4** appeared to have a privileged scaffold over the other lariats as well as the linear control **Lar0** for permeability into *Mtb* (Fig. 4e).

We appreciated that the type of cyclization could influence the conformation of the peptide, structural rigidity, and in-turn, its interaction with the mycomembrane. It had been previously shown that in a mammalian system, the structural features of the linker can be an element that alters accumulation of peptides.^79^ To this end, we expanded our thioether peptide library to include a series of peptides cyclized using two cysteine side chains. First, we synthesized the dithiol molecule **Dit1**, which possesses the same sequence as **Cyc0**, except for an additional cysteine residue on the *N*-terminus, which was *N*-acetylated (Fig. 4f). In one configuration, the dithiol peptide was oxidized to generate a disulfide bond between the two cysteine side chains. Notably, disulfide-cyclized peptides are increasingly being examined as drug candidates and therapeutic agents.^80–84^ Additionally, we also synthesized peptide **Dit2** by reacting the linear dithiol **Dit1** scaffold with a bis-electrophilic linker, resulting in a peptide with two thioether bonds (Fig. 4f). These types of linkers are extensively used in the construction of macrocyclic peptide libraries due to their broad sequence compatibility and suitability for late-stage conformational diversification, prompting us to investigate a test case.^85–88^ Lastly, we synthesized a linear control **Dit0**, replacing the cysteine residues with serine (Fig. 4f). Amongst this sub-library, we observed that the linear control **Dit0** exhibited superior permeability, in comparison to its macrocyclic counterparts **Dit1** and **Dit2** (Fig. 4g). For **Dit1**, this may be due in part to interception by thiol/disulfide-displaying proteins in the cell envelope before it has the chance to reach the PG. Thiol-disulfide exchange reactions between disulfide containing molecules and exofacial protein thiols/disulfides have previously been shown to modulate permeability.^89,90^ This observation also crucially highlights the impact that the cyclization chemistry can have on permeation, given that **Cyc0** and **Dit1**, despite differing by only a single amino acid and mode of cyclization, demonstrate significantly different permeabilities. As far as **Dit2** is concerned, we pose that the hydrophobicity of the linker may be a factor in its ability to permeate the mycomembrane. Notably, there is some consideration to be made that the low fluidity and increased thickness of the mycomembrane could impede the diffusion and hence accumulation of more lipophilic molecules.^91,92^ Interestingly, the same peptides in *Mtb* revealed no significant differences in the permeability of **Dit0**, **Dit1** and **Dit2** (Fig. 4g). As before, we ensured that the macrocyclization library readily reacted with our DBCO modified beads (Fig. S12) and the cell integrity remained unaffected upon treatment with the peptides based on EtBr accumulation experiments (Fig. S13). Taking it together, our data suggests a general trend that macrocyclization can improve molecule accumulation past the mycomembrane although peptide chemistry and ring size must be carefully considered as they may play a significant role in influencing permeability.

**Figure 5.**
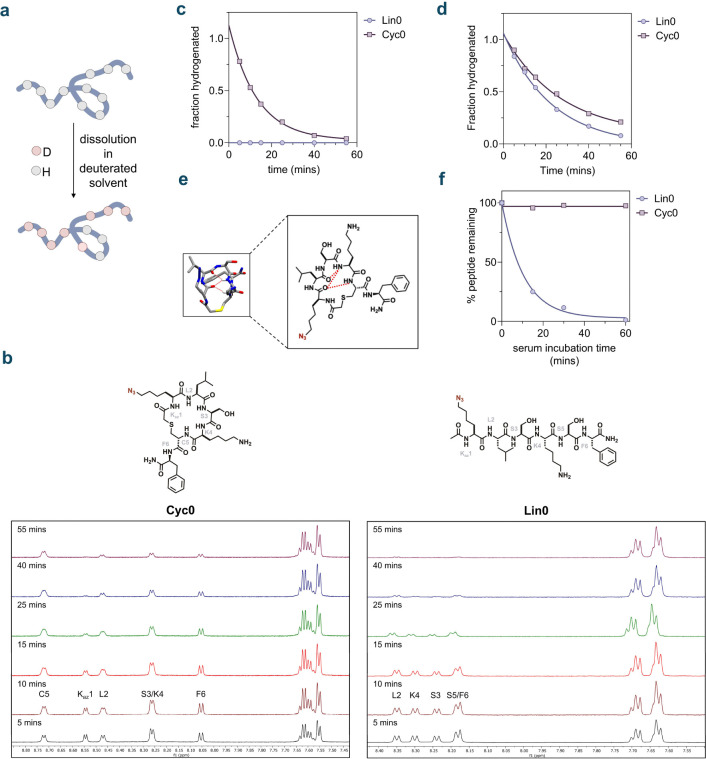
**(a)** Schematic illustration of hydrogen-deuterium exchange (HDX) of peptides. Peptides are labeled in a D_2_O buffer followed by exchange quenching, the rate of which is determined by *N-H* accessibility to the solvent **(b)** ^1^H-NMR spectra illustrating the time-dependent HDX of backbone amides in **Lin0** and **Cyc0** over 1 h **(c)** Fraction of (K_az_1) amide of **Lin0** and **Cyc0** hydrogenated over time upon HDX of the peptides **(d)** Fraction of (L2) amide of **Lin0** and **Cyc0** hydrogenated over time upon HDX of the peptides **(e)** 3-D representation and chemical structure of **Cyc0** showing hydrogen bonding observed through molecular dynamics simulations **(f)** Comparison of the serum stability of **Lin0** and **Cyc0** over 1 h. For serum stability, one-phase decay curves were fitted to the data using GraphPad Prism.

We then sought to decipher the mechanistic basis for the enhanced accumulation of the macrocyclic molecules compared to their linear counterparts. Notably, upon evaluating the physicochemical properties of both our libraries, we found that most library members, including the most effective accumulators were well outside the rule-of-five (Ro5) features established by Lipinski^11^ as well as Veber^93^ (Fig. S14). This finding further underscores a significant gap in the understanding of the molecular characteristics that enable traversing of the mycomembrane. It is well-established that intramolecular hydrogen bonding and the solvent-accessible surface area are critical factors influencing permeability across membrane bilayers.^94,95^ This understanding often underpins the rationale for both macrocyclized^67,96^ and *N*-methylated^97^ peptides to improve membrane permeability by reducing solvent accessibility. Therefore, we aimed to determine if differences in amide *N−H* solvent accessibility were responsible for the variations we observed in mycomembrane permeability between the linear and macrocyclic molecules. To this end, we conducted hydrogen/deuterium exchange (HDX) experiments with molecules **Cyc0** and **Lin0** using ^1^H-NMR (Fig. 5a). Importantly, for this purpose, we characterized the molecules using 1D and 2D-NMR, assigning all *N-H* resonances. Our results showed that, in general, the backbone amides of **Lin0** exchanged much more readily than those of **Cyc0**, showing complete exchange within an hour (Fig. 5b). Of note is the azido-lysine (K_Az_) amide hydrogen of **Lin0** which showed complete exchange within the dead-time (~5 mins) (Fig. 5c). In contrast, the K_Az_ amide hydrogen of **Cyc0** did not fully exchange even at the 25 min mark (Fig. 5c). To further compare these two peptides, we focused on the L2 amide backbone as it was present in both molecules, located within the macrocyclic region of **Cyc0**, and well-resolved in the NMR (Fig. 5d). The L2 amide backbone of cyc1 exhibited a half-life of 18.36 minutes, compared to 15.65 minutes for **Lin0** (Fig. 5d). This observation indicates that macrocyclization is likely to contribute to the shielding of the L2 amide backbone, potentially leading to slower exchange. The **Cyc0** L2 backbone amide could also be participating in intramolecular hydrogen-bonding that could also affect HDX. MD simulation studies also revealed that the backbone amides of the **Cyc0** molecule participate in intramolecular hydrogen-bonding (Fig. 5e). Notably, no intramolecular hydrogen-bonding was observed for its linear counterpart **Lin0**.

Another biochemical consequence of cyclization of peptides that is well appreciated is the potential for greater resistance to proteases.^98–100^ This protection may be due in part to the inability of the macrocyclic peptide to bind the active site of serine proteases in the correct conformation given its limited flexibility.^99,101^ Additionally, the shielding of the backbone upon macrocyclization, as observed in our hydrogen/deuterium exchange experiments, likely contributes to this protection. Upon testing the metabolic stability of both **Cyc0** and **Lin0**, we observed that **Lin0** fully degraded within 1 h of serum incubation while cyc1 remained stable with no significant degradation (Fig. 5f). We also observed this in the case of backbone *N*-methylation, where peptide **Nmet5** was found to be considerably more serum-stable than peptide **Nmet0**, likely owing to the steric hindrance it introduces towards serine proteases (Fig. S15).

### Effect of Structural Edits on a Peptide Antibiotic

Our data provides the first empirical descriptions that the strategic incorporation of amide backbone methylation and cyclization can significantly enhance the accumulation of molecules within mycobacteria. Building on these findings, we next set out to apply these molecular edits to a peptidic antibiotic that initially not known to have activity in mycobacteria. The goal is to determine whether these structural changes can transform its accumulation properties and boost its antibacterial effectiveness, potentially leading to more potent therapeutic agents against mycobacterial infections. We decided to investigate the effect of backbone *N*-methylation and macrocyclization on the peptide antibiotic Tridecaptin A1 with the aim to improve its efficacy against mycobacteria. Tridecaptin A1 is a non-ribosomal lipopeptide with known antimicrobial activity against Gram-negative bacteria; it is theorized that its mode of action is primarily driven by its ability to bind to the bacterial cell wall precursor lipid II in the inner membrane and subsequently disrupt the proton motive force.^102,103^ However, to the best of our knowledge, there are no reports on the activity of Tridecaptin A1 against mycobacteria.

**Figure. 6.**
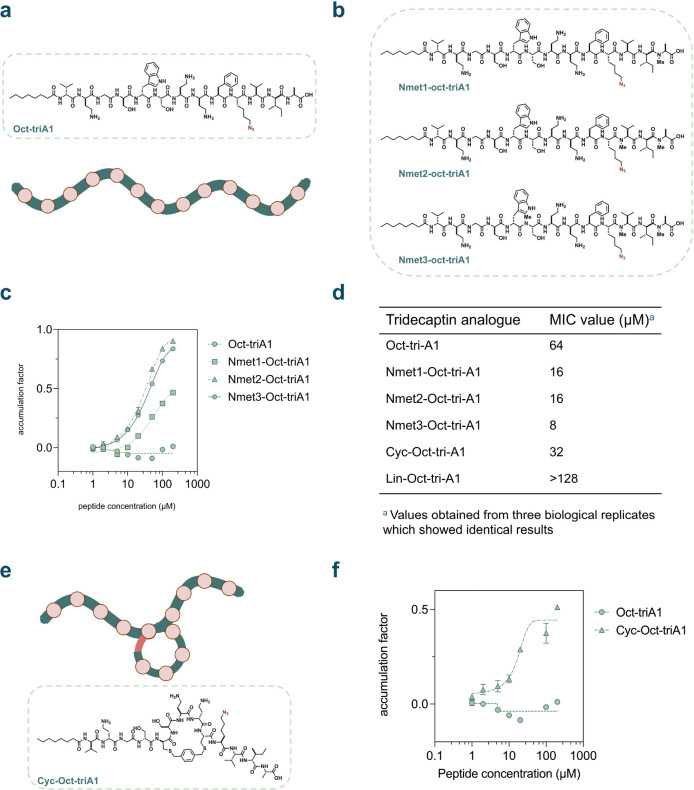
Accumulation and biological activities of tridecaptin derivatives. **a**, Schematic illustration and chemical structure of **Oct-triA1**. The azide group is installed on the peptide on the azido-lysine side chain replacing the glutamate acid residue in position 10. **b**, Chemical structures of *N*-methyl derivatives of **Oct-triA1**. **c**, Concentration dependent accumulation of **Oct-triA1** and its *N*-methyl derivatives. DBCO modified mycobacterial cells were incubated with different concentrations of peptides for 1 h, then treated with 50 mM **Fl-az** for 1 h. **d**, MIC values for the tridecaptin analogues. **e**, Schematic illustration and chemical structure of cyclic derivative of **Oct-triA1**. The peptide is cyclized between two cysteines at positions 5 and 9 linked by phenyl linker. **f**, Concentration dependent accumulation of **Oct-triA1** and its cyclic derivative. DBCO modified mycobacterial cells were incubated with different concentrations of peptides for 1 h, then treated with 50 mM **Fl-az** for 1 h. Fluorescence intensities were measured by flow cytometry and normalized to the negative control in each experiment (data not shown). Accumulation factors are calculated as the subtraction of normalized intensities from 1. Data points and error bars represent the average and s.d. from three technical repeats, respectively.

Considering that the mycobacterial lipid II and the Gram-negative lipid II share structural similarities, we hypothesized that Tridecaptin A1 could potentially be modified with backbone *N*-methylation or macrocyclization to improve its activity against mycobacteria by enhancing its accumulation past the mycomembrane. In the redesign of Tridecaptin, we noted that the addition of an octanoyl lipid on the *N*-terminus of Tridecaptin A1 was found to be well tolerated.^104^ An alanine scan of the Octyl-Tridecaptin A1 analogs revealed that amino acid residues in positions 1, 4, 6, 10, 11 can be substituted to alanine without significantly compromising its activity against Gram-negative pathogens.^105^ Further, it was also reported that the side chain carboxylate of Glu10 can be conjugated with antibiotic warheads to improve its activity *in vitro*.^106^ Relying on these previous efforts elucidating the activity of this peptide, we designed a series of Octyl-Tridecaptin A1 analogues with the Glu10 residue substituted to an azido bearing lysine analog for compatibility with our assay. Based on this substitution, we synthesized **Oct-triA1** to serve as a control for our structural edits (Fig. 6a).

For *N*-methylation, sites were selected by examining the reported solution NMR structure of the Tridecaptin A1-lipid II complex. We, consequently, synthesized peptide variants wherein residues Ser6, Val11 or Ala13 were methylated. Specifically, we generated monomethylated (**Nmet1-Oct-triA1** with meAla13), dimethylated (**Nmet2-Oct-triA1** with meVal11 and meAla13), or trimethylated (**Nmet3-Oct-triA1** with meSer6, meVal11, meAla13) analogs of **Oct-triA1** (Fig. 6b). The dose-response accumulation profiles of the *N*-methylated **Oct-triA1** series peptides were then analyzed *via* live cell PAC-MAN in *Msm* (Fig. 6c and Fig. S16). Notably, we observed that **Nmet1-Oct-triA1** as well as **Nmet2-Oct-triA1** exhibited better accumulation when compared to unmethylated **Oct-triA1**. However, **Nmet3-Oct-triA1** had slightly worse accumulation than **Nmet2-Oct-triA1**, suggesting that the additional methylation on Ser6 has a negative effect on its permeability into mycobacteria. The unmethylated peptide **Oct-triA1** demonstrated fluorescence intensities higher than the vehicle control at certain lower concentrations, resulting in negative accumulation factor values (Fig. S16). We attributed this observation to potential cell envelope disruption by **Oct-triA1**, which promotes the permeability of the fluorophore and higher absolute fluorescence intensities. Indeed, EtBr accumulation experiments showed that incubation with control peptide **Oct-triA1** significantly affected the cell envelope integrity (Fig. S17). No significant effect was observed in the case of Nmet1-Oct-triA1, Nmet2-Oct-triA1 and **Nmet3-Oct-triA1**. Importantly, we also ensured that the peptides readily reacted with our **TetD** labelled beads (Fig. S18). We next studied the effect of *N*-methylation on the antimicrobial activity of **Oct-triA1** against *Msm* via a Minimal Inhibitory Concentration (MIC) assay. Remarkably, we observed a marked decrease in the MIC value of the peptide with increasing degree of methylation with trimethylated **Nmet3-Oct-triA1** exhibiting an 8-fold drop in MIC value compared to unmethylated **Oct-tri-A1** (**Table 1**). Overall, these data indicated that backbone *N*-methylation of **Oct-tri-A1** enhanced its activity against *Msm*.

We next sought to analyze the effect of macrocyclization on the activity of **Oct-tri-A1** against *Msm*. Previously, Ballantine et al. had reported cyclic analogues of Octyl-Tridecaptin A1 wherein d-Trp5 and l-Phe9 were replaced with d- and l- cysteine residues, respectively, and the peptides were subsequently cyclized *via* bis-electrophilic linkers.^107^ This was done with the aim to replace the reported π-stacking interactions between d-Trp5 and l-Phe9 with a covalent linkage as well as to impart resistance against peptidases.^107^ Adopting a similar template, we synthesized **Cyc-Oct-tri-A1** wherein the cysteine residues were cyclized with a bis-bromomethyl-benzene linker and the Glu10 residue was substituted to an azido bearing lysine analog (Fig. 6d). The dose-response accumulation profile of **Cyc-Oct-tri-A1** was then analyzed *via* live cell PAC-MAN in *Msm* (Fig. 6e and Fig. S19). We observed that **Cyc-Oct-tri-A1** exhibited better accumulation across the mycomembrane when compared to the control peptide **Oct-triA1**. To further elucidate t the impact of macrocyclization, we also synthesized **Lin-Oct-tri-A1** where the d-Trp5 and l-Phe9 residues were replaced with d- and l- serine residues, respectively (Fig. S20a), to serve as a linear control. Interestingly, we observed no significant difference in accumulation between **Cyc-Oct-tri-A1** and **Lin-Oct-tri-A1** (Fig. S20b and Fig. S20c). Further, EtBr accumulation experiments revealed that **Cyc-Oct-tri-A1** and **Lin-Oct-tri-A1** had no significant effect on cell envelope integrity (Fig. S21). The peptides also readily reacted with our **TetD** labelled beads (Fig. S22).

Next, we sought to examine the antimicrobial activity of **Cyc-Oct-tri-A1** and **Lin-Oct-tri-A1** against *Msm*. In the case of **Cyc-Oct-tri-A1**, we also observed a 2-fold drop in the MIC value when compared to control peptide **Oct-triA1** (**Table 1**). Remarkably, **Cyc-Oct-tri-A1** exhibited over a 4-fold improvement in antimicrobial activity in comparison to **Lin-Oct-tri-A1**. Taken together, these data suggest that macrocyclization improves the antimicrobial activity of **Oct-triA1** against *Msm*.

## DISCUSSION

Peptides typically exceed Lipinski’s Rule of Five for drug-likeness in terms of their size and the number of hydrogen bond donors and acceptors on their backbone. Our choice to investigate *N*-alkylation as a design principle was prompted by its widespread use to eliminate hydrogen bond donors on the backbone. This elimination reduces the number of hydrogen bonds formed with the solvent, ultimately decreasing the desolvation penalty associated with passive membrane diffusion. Some peptides (or other molecules in general) can also exhibit chameleonic behavior in their passive diffusion through membranes, which can be favored by backbone alkylation.^34,35^ Indeed, *N*-methylation is used as an effective approach to improve peptide permeability through mammalian cell membranes.^35,53–55,108^ A similar approach to reduce the number of backbone hydrogen bond donors involves shifting the peptide side chains from the α-carbon to the backbone nitrogen atom to create peptidomimetics, i.e. peptoids. Peptoids are easier to synthesize from primary amines and allow the possibility of versatile side chain libraries – albeit at the cost of chiral centers on the backbone.^109^ Peptoids are known to have improved permeability through biological membranes, both in mammalian cells^60^ as well as bacterial cells and have promising potential as effective therapeutics against drug-resistant bacterial pathogens.^61–65^ Another well observed structural feature of bioactive large peptidic molecules is their macrocyclization. Macrocyclization can promote passive permeability by encouraging intramolecular hydrogen-bonding and reducing the solvent-exposed surface area of the peptide. As a result, the number of hydrogen-bonds formed with the solvent decreases, thereby mitigating the desolvation penalty associated with membrane permeation. Further, it can also improve other pharmacological properties such as providing increased rigidity^110^ and metabolic stability^16,111^, which may be especially relevant in therapeutic contexts.

Deciphering the principles that govern molecular accumulation in mycobacteria is central for achieving high antimycobacterial efficacy. A comprehensive understanding of how structures drive accumulation will facilitate the identification of high-potential drug candidates and enable the strategic redesigning of existing chemical scaffolds to enhance their accumulation within mycobacterial cells. Our high-throughput assay format in PAC-MAN enables one to make various specific and systematic evaluations of design principles that affect the accumulation of molecules across the mycomembrane. Leveraging this platform, here we report that, in general, macrocyclization and *N*-alkylation generally promoted the accumulation of our model peptides across the mycomembrane. Upon applying these design principles to a peptide antibiotic Tridecaptin A1, we were able to demonstrate improved efficacy against mycobacteria.

## Supplementary Material

Supplement 1
