# Supplementary material for: Systematic Determination of the Impact of Structural Edits on Accumulation into Mycobacteria
Source: bioRxiv. 2025 Jan 18:2025.01.17.633618. Preprint. [Version 1] doi: 10.1101/2025.01.17.633618 (PMC11760776; doi:10.1101/2025.01.17.633618)
Supplement: Supplementary file 1 [file media-1.pdf]

SUPPORTING INFORMATION

Systematic Determination of the Impact of Structural Edits on Accumulation into Mycobacteria

Rachita Dash[1,2#], Zichen Liu[1,2#], Irene Lepori[3,4], Mahendra D. Chordia[1,2], Karl Ocius[1,2], Kadie Holsinger[1,2], Han Zhang[4,5], Ryan Kenyon[1,2], Wonpil Im[4,5], Sloan Siegrist[3,4], and Marcos M. Pires[1,2*]

[1] Department of Chemistry
[2] Department of Microbiology, Immunology, and Cancer
University of Virginia
Charlottesville, VA 22904, USA

[3] Molecular and Cellular Biology Graduate Program
[4] Department of Microbiology
University of Massachusetts, Amherst, MA USA

[5] Department of Chemistry
[6] Departments of Biological Sciences and Bioengineering
Lehigh University
Bethlehem, PA 18015, USA

* corresponding author: mpires@virginia.edu

TABLE OF CONTENTS

**SUPPLEMENTARY FIGURES**	S4-S25
**EXPERIMENTAL PROCEDURES**	S26-S76
**Materials**	S26-S28
**Biological methods**	S28-S31
Mycobacteria cell culture	S28
DBCO-tetrapeptide (**TetD**) based labeling of *Msm*	S28
DBCO-tetrapeptide (**TetD**) based labeling of *Mtb*	S28
Permeability assay (PAC-MAN) across the *Msm* mycomembrane	S28
Permeability assay (PAC-MAN) across the *Mtb* mycomembrane	S28
Isolation of peptidoglycan and muropeptides	S29
Ethidium bromide (EtBr) whole-cell accumulation assay	S29
DBCO-modified polystyrene bead reactivity assay	S30
Calculation of physiochemical properties of test molecules	S30
Hydrogen-deuterium exchange (HDX) studies	S30
Molecular dynamics (MD) simulation studies	S30
Metabolic stability assay	S31
Minimum inhibitory concentration (MIC) assay	S31
**Synthesis and characterization of test molecules**	S32-S76
General procedure for the solid-phase synthesis of peptides	S32
Synthesis of TetD	S32

Synthesis of the macrocyclic peptide library	S32
Synthesis of the *N*-alkylated peptide library	S33
Synthesis of the Tridecaptin series	S33
Characterization of test molecules	S34-S76
**REFERENCES**	S77

SUPPLEMENTARY FIGURES

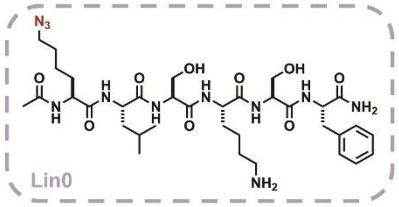

Figure S1. Chemical structure of peptide Lin0

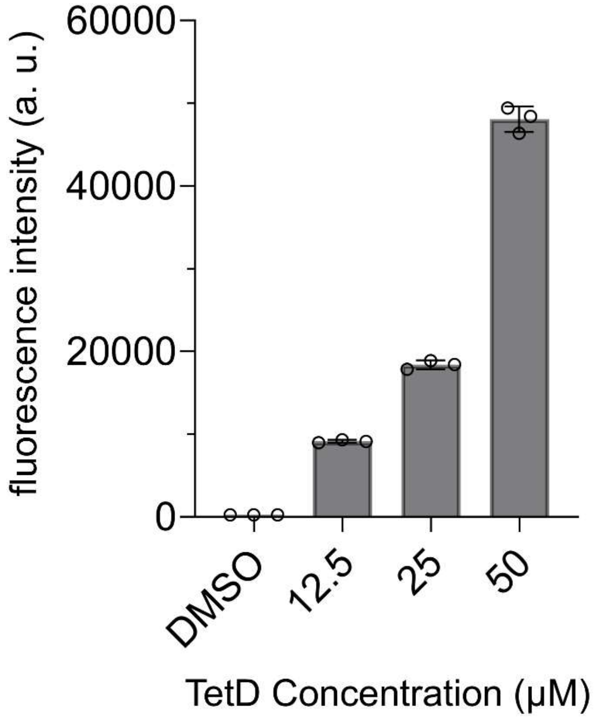

Figure S2. Concentration dependent labelling of *Msm* treated with different concentrations of TetD. Mid-log phase mycobacteria mc2 155 were incubated with different amounts of TetD for 18 h and then treated with **Fl-az.** Data are represented as mean ± SD (n= 3).

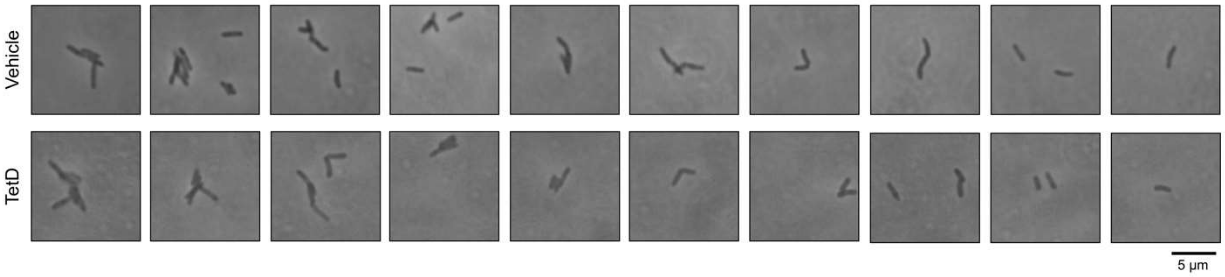

Figure S3. Phase contrast imaging of *Mtb* cells treated with vehicle or TetD.

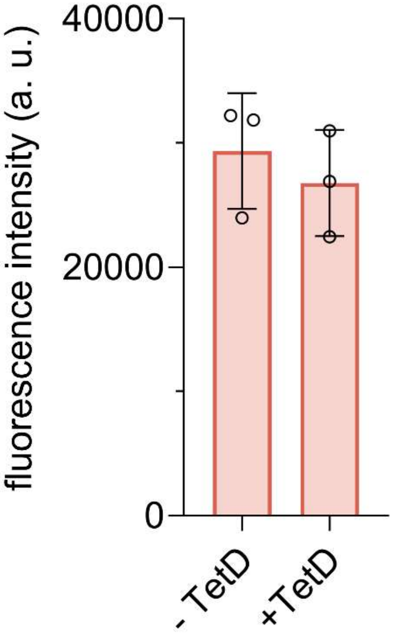

Figure S4. Ethidium Bromide based mycomembrane integrity analysis of *Msm* cells labeled with 25 μM **TetD**. Treatment with DMSO (**-TetD**) served as the control. The fluorescence intensity reported was measured at the end of 60 minutes. Data are represented as mean ± SD (n= 3).

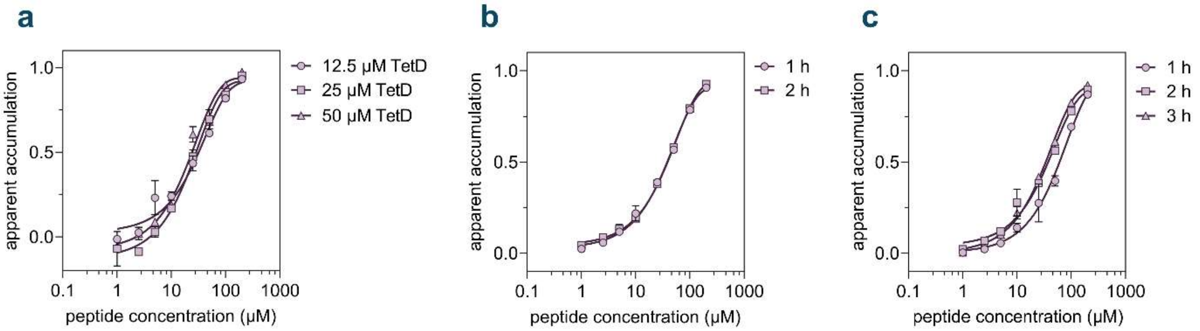

Figure S5. (a) Concentration dependent accumulation of **Lin0** with bacteria cells treated with different concentrations of **TetD**. Mid-log phase mycobacteria mc[2] 155 were incubated with different amounts of **TetD** for 18 h and then used to determine apparent accumulation of **Lin0** across the mycomembrane. **(b)** Dose-response analysis of apparent accumulation of **Lin0** with varying **Fl-az** treatment durations *via* PAC-MAN. **(c)** Dose-response analysis of apparent accumulation of **Lin0** with varying **Lin0** treatment durations *via* PAC-MAN. For dose-response curves, Boltzmann sigmoidal curves were fitted to the data using GraphPad Prism.

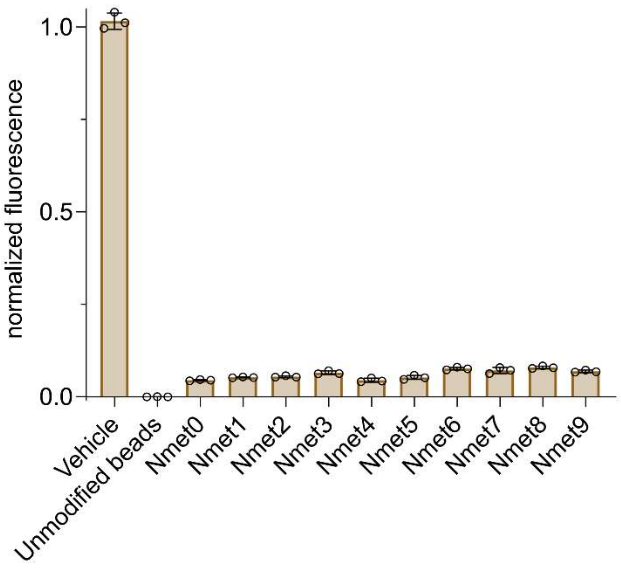

Figure S6. DBCO-modified polystyrene bead reactivity assay with the *N*-methylation library. Treatment of modified with DMSO instead of azido-peptides (vehicle) and unmodified beads, served as controls. Data are represented as mean ± SD (n= 3).

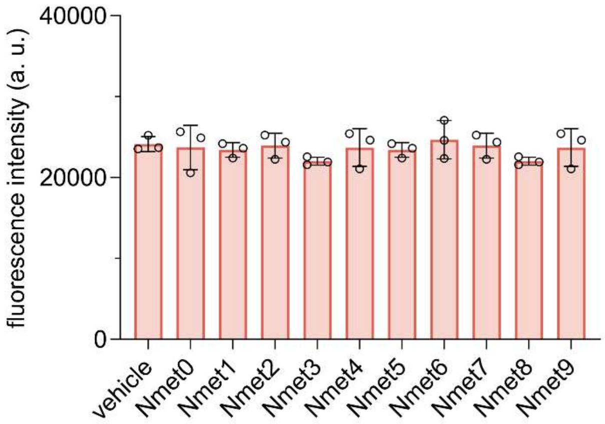

Figure S7. Ethidium Bromide based mycomembrane integrity analysis of **TetD** labeled *Msm* cells treated with the *N*-methylation library**.** Treatment with DMSO (vehicle) served as the control. Data are represented as mean ± SD (n= 3).

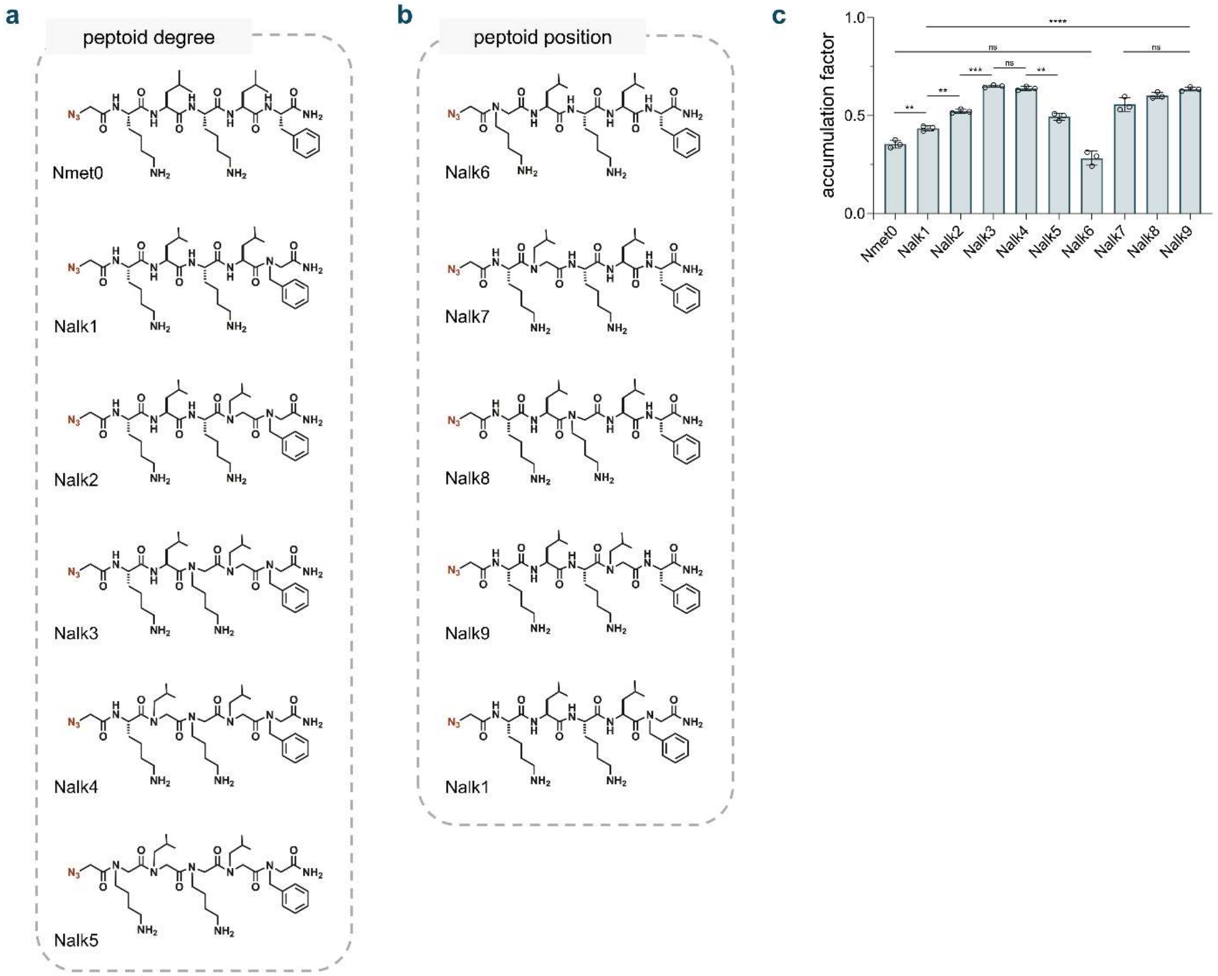

Figure S8. (a) Chemical structures of the *N*-alkylation (peptoid) sub-library members with varying degrees of backbone *N*-alkylation (**Nmet0**-**Nalk5**). **(b)** Chemical structures of the *N*-alkylation sub-library members with varying positions of backbone *N*-alkylation (**Nalk6**-**Nalk9** and **Nalk1**). **(c)** Apparent accumulation of the *N*-alkylation library across the mycomembrane in *Msm* after 1 h of treatment with 50 μM compound. Data are represented as mean ± SD (n= 3). P-values were determined by a two-tailed t-test (ns = non-significant, ** $p < 0.01$, *** $p < 0.001$, **** $p < 0.0001$).

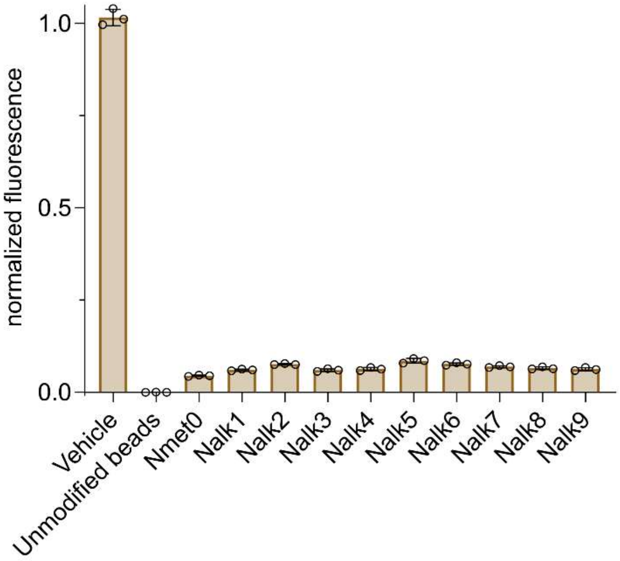

Figure S9. DBCO-modified polystyrene bead reactivity assay with the *N*-alkylation library. Treatment of modified with DMSO instead of azido-peptides (vehicle) and unmodified beads, served as controls. Data are represented as mean ± SD (n= 3).

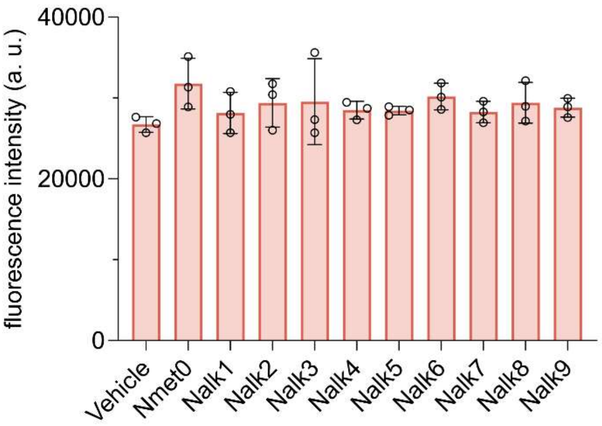

Figure S10. Ethidium Bromide based mycomembrane integrity analysis of **TetD** labeled *Msm* cells treated with the *N*-alkylation library**.** Treatment with DMSO (vehicle) served as the control. Data are represented as mean ± SD (n= 3).

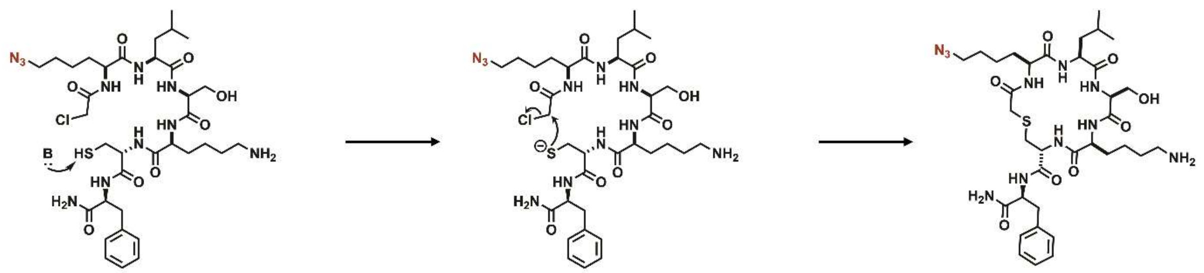

Figure S11. Scheme illustrating the cyclization chemistry adopted for the synthesis of the thioether based macrocyclic peptides.

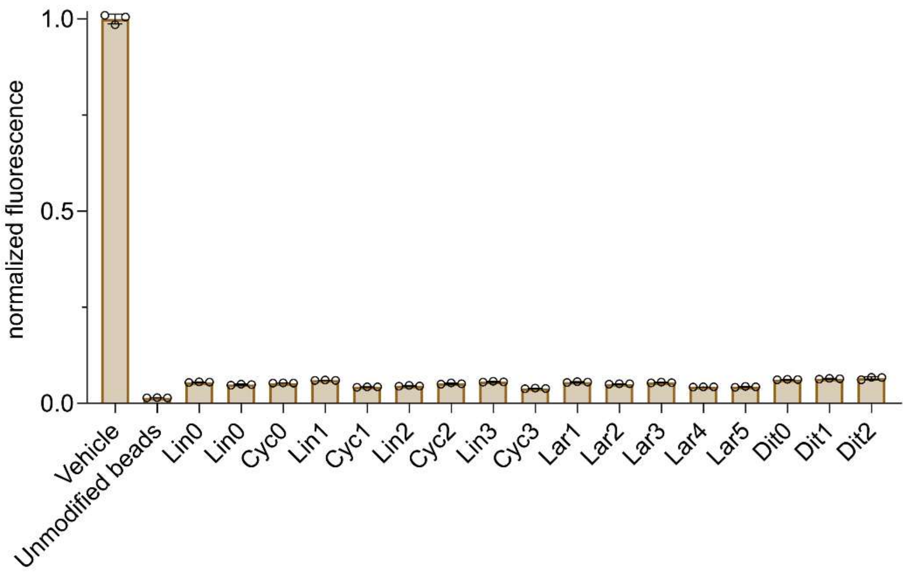

Figure S12. DBCO-modified polystyrene bead reactivity assay with the macrocyclization library. Treatment of modified with DMSO instead of azido-peptides (vehicle) and unmodified beads, served as controls. Data are represented as mean ± SD (n= 3).

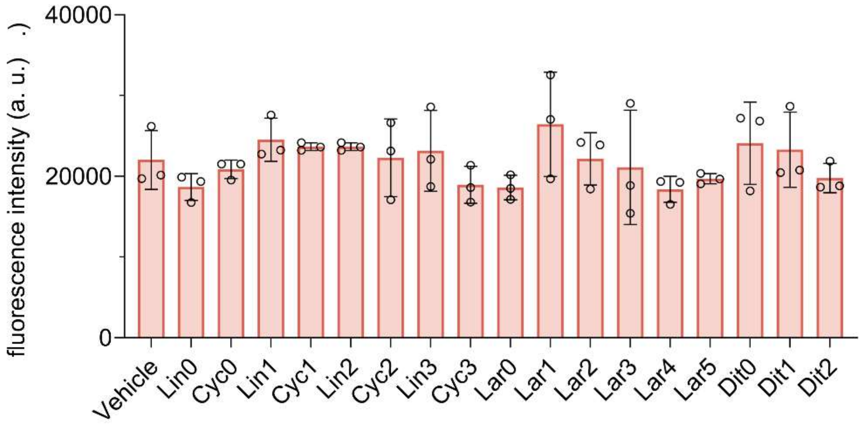

Figure S13. Ethidium Bromide based mycomembrane integrity analysis of **TetD** labeled *Msm* cells treated with the macrocyclization library**.** Treatment with DMSO (vehicle) served as the control. Data are represented as mean ± SD (n= 3).

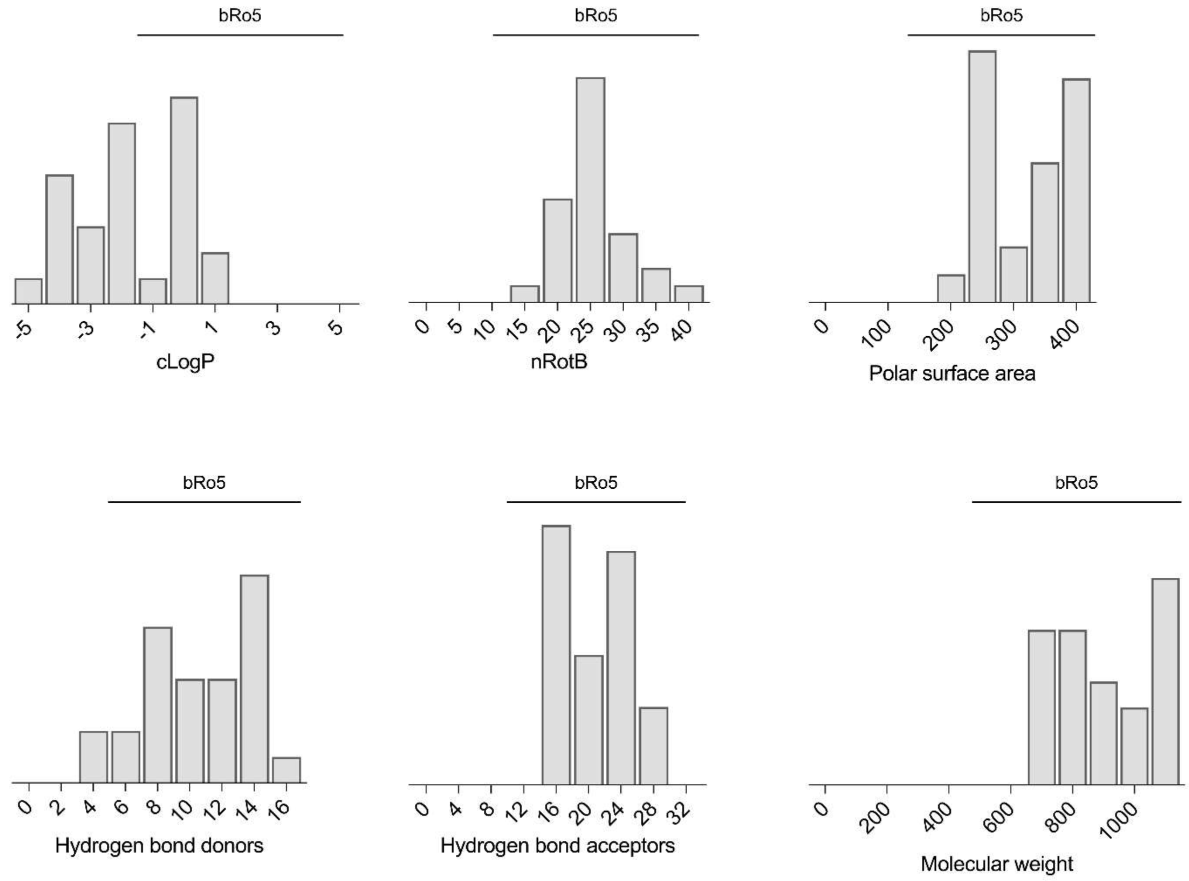

Figure S14. Frequency distributions of cLogP, number of rotatable bonds (nRotBs), polar surface area, number of hydrogen bond donors, number of hydrogen bond acceptors and molecular weights of the azido-peptides in this study. Chemical spaces beyond the rule-of-five (Ro5) are highlighted.

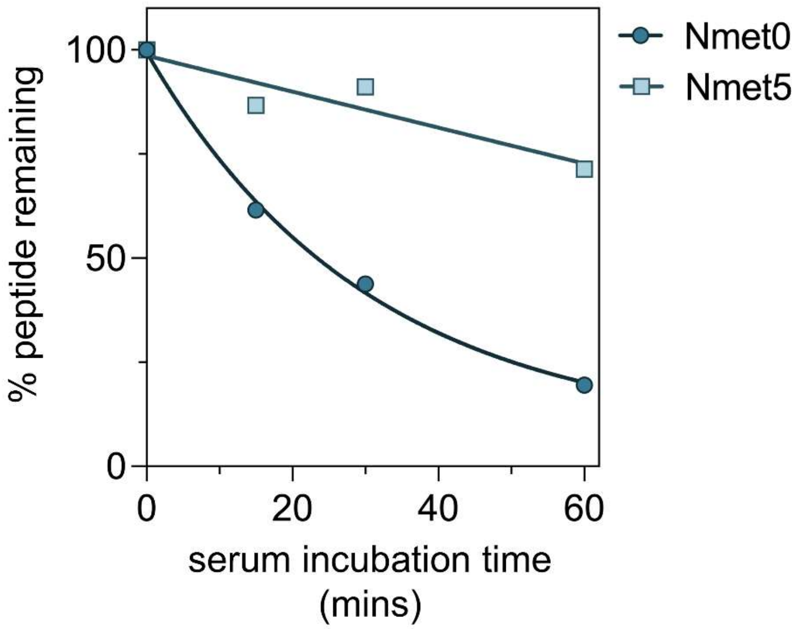

Figure S15. Comparison of the serum stability of **Nmet0** and **Nmet5** over 1 hour. One-phase decay curves were fitted to the data using GraphPad Prism.

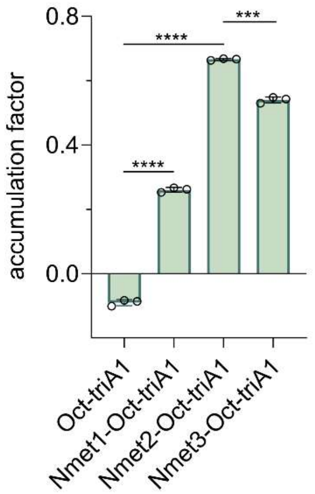

Figure S16. Apparent accumulation of the *N*-methylated tridecaptin series across the mycomembrane in *Msm* after 1 h of treatment with 50 μM compound. Data are represented as mean ± SD (n= 3). P-values were determined by a two-tailed t-test (*** $p < 0.001$, **** $p < 0.0001$).

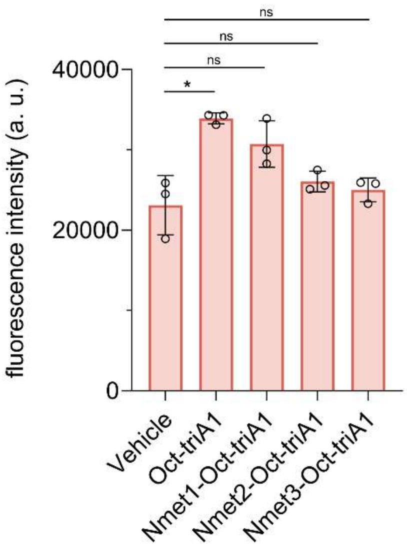

Figure S17. Ethidium Bromide based mycomembrane integrity analysis of **TetD** labeled *Msm* cells treated with the *N*-methylated tridecaptin series**.** Treatment with DMSO (vehicle) served as the control. Data are represented as mean ± SD (n= 3).

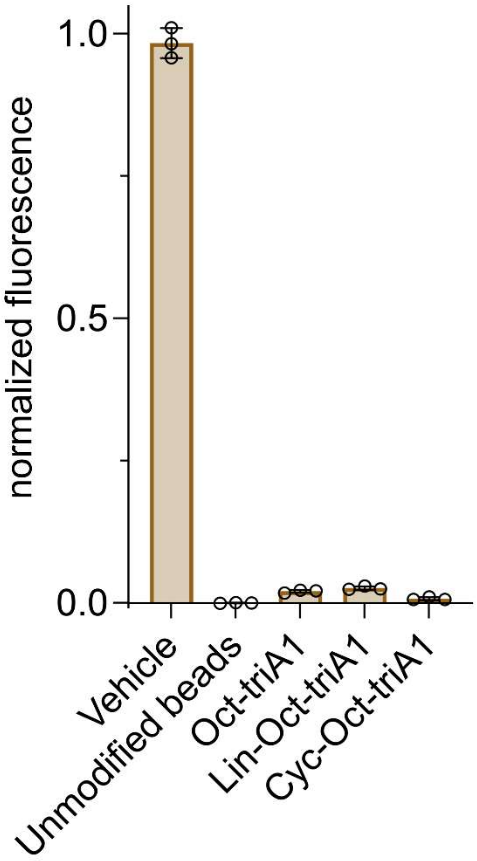

Figure S18. DBCO-modified polystyrene bead reactivity assay with the *N*-methylated tridecaptin series. Treatment of modified with DMSO instead of azido-peptides (vehicle) and unmodified beads, served as controls. Data are represented as mean ± SD (n= 3).

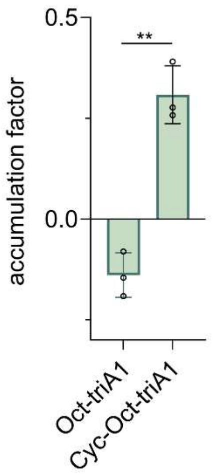

Figure S19. Apparent accumulation of the macrocyclic tridecaptin analogue compared to unmodified tridecaptin across the mycomembrane in *Msm* after 1 h of treatment with 50 µM compound. Data are represented as mean ± SD (n= 3). P-values were determined by a two-tailed t-test (*** p < 0.001, **** p < 0.0001).

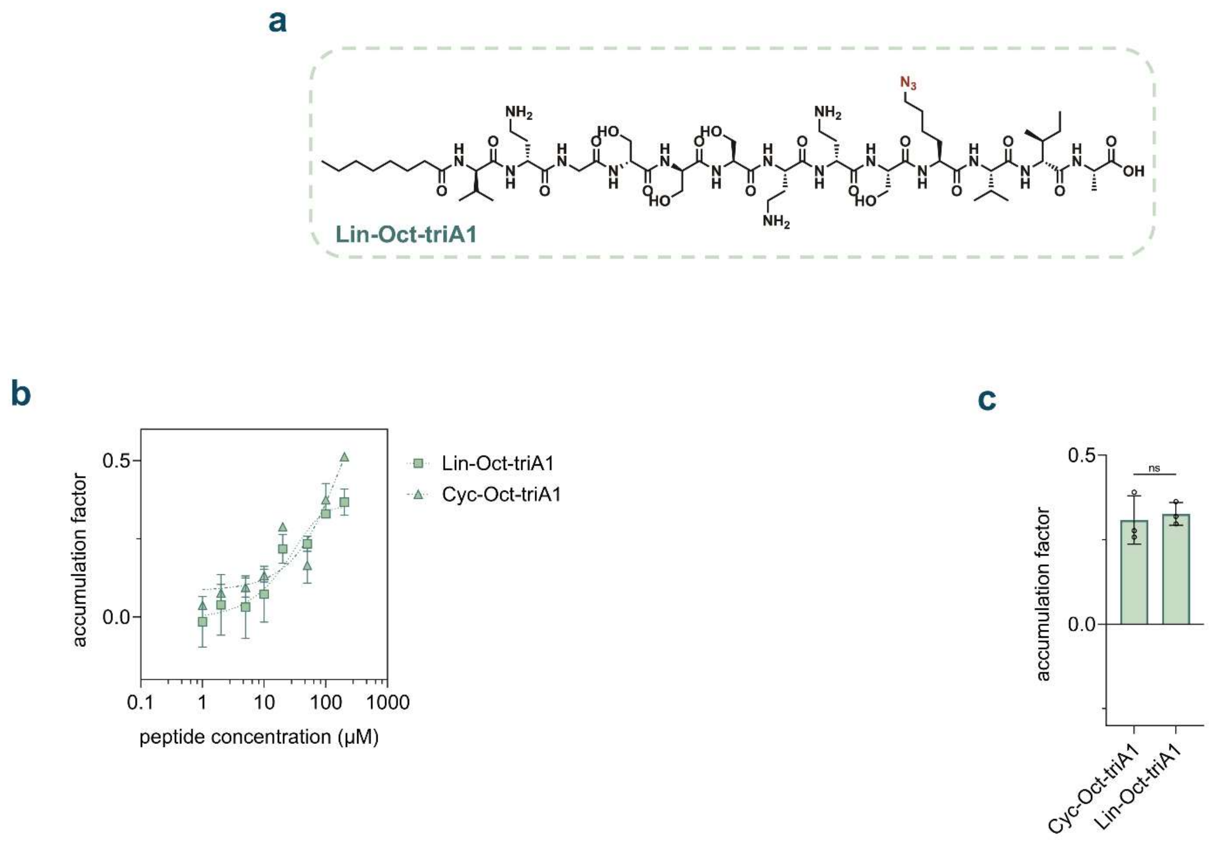

Figure S20. (a) Chemical structure of linear tridecaptin analogue **(b)** Dose-response analysis of apparent accumulation of Lin-Oct-triA1 and Cyc-Oct-triA1. **(c)** Apparent accumulation of the macrocyclic tridecaptin analogue compared to the linear tridecaptin analogue across the mycomembrane in *Msm* after 1 h of treatment with 50 µM compound. Data are represented as mean ± SD (n= 3). P-values were determined by a two-tailed t-test (*** $p < 0.001$, **** $p < 0.0001$). For dose-response curves, Boltzmann sigmoidal curves were fitted to the data using GraphPad Prism.

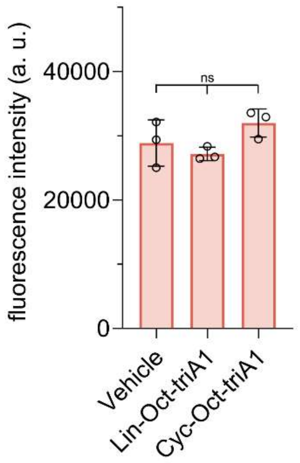

Figure S21. Ethidium Bromide based mycomembrane integrity analysis of **TetD** labeled *Msm* cells treated with the macrocyclic and linear tridecaptin analogues**.** Treatment with DMSO (vehicle) served as the control. Data are represented as mean ± SD (n= 3).

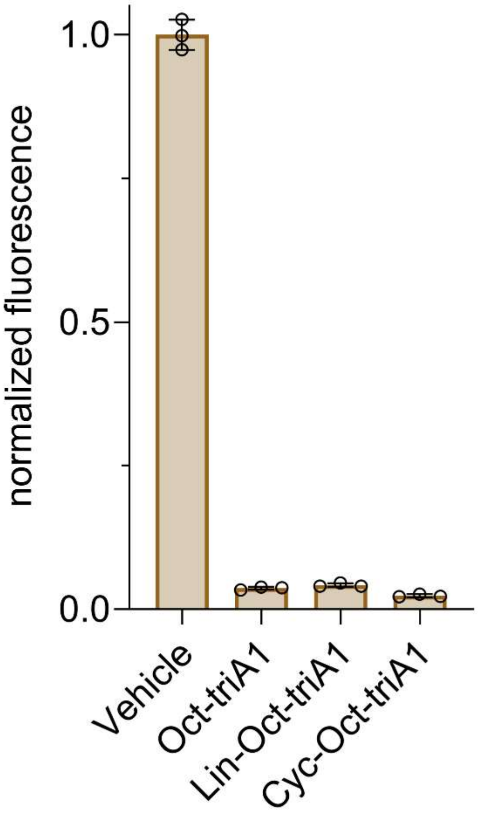

Figure S22. DBCO-modified polystyrene bead reactivity assay with the macrocyclic and linear tridecaptin analogues. Treatment of modified with DMSO instead of azido-peptides (vehicle) and unmodified beads, served as controls. Data are represented as mean ± SD (n= 3).

EXPERIMENTAL PROCEDURES

Materials

REAGENTS	VENDOR SOURCE	CATALOG #
**Reagents for Biological Methods**		
Middlebrook 7H9 media	VWR	90003-876
Catalase from bovine liver	Millipore Sigma	C1345-1G
D(+)-Glucose, anhydrous (Dextrose)	Chem Impex	00805
Bovine serum albumin fraction V	Millipore Sigma	10735078001
Glycerol	Millipore Sigma	G7893
Tween 80	VWR	97061-674
Formaldehyde solution	Millipore Sigma	252549
6-azido-fluorescein **(Fl-az)**	Lumiprobe	D5130
TAMRA azide, 6-isomer **(TMR-az)**	Lumiprobe	D8130
sulfo-Cyanine3 azide **(sCy3-az)**	Lumiprobe	B1330
sulfo-Cyanine5 azide (**sCy5-az)**	Lumiprobe	C3330
3-azido-7-hydroxy coumarin (**Co-az**)	Millipore Sigma	909513-5MG
Amino terminated polystyrene beads	Spherotech	AP-30-10
Trichloroacetic acid solution	Millipore Sigma	T0699
Ethidium bromide solution (10 mg/mL), Biotechnology Grade	VWR	97064-970
Protease inhibitor	Millipore Sigma	11836153001
SDS	Millipore Sigma	436143
Proteinase K	Millipore Sigma	SAE0009-1G
Mutanolysin	Millipore Sigma	M9901-10KU
Lysozyme	MP Biomedicals	100834
**Reagents for Chemicals Synthesis and Characterization of Test Molecules**		
α-*N*-Fmoc-amino acids	Chem Impex	Various
N-methylated amino acids	Chem Impex	Various
2-Chlorotrityl chloride resin (1.0-2.0 meq/g, 100-200 mesh	Chem Impex	03498
Rink amide resin, (0.3-0.6 meq/g, 100-200 mesh)	Chem Impex	12662
N, *N'*-Diisopropylethylamine (DIEA)	Chem Impex	00141
Hexafluorophosphate Benzotriazole Tetramethyl Uronium (HBTU)	Chem Impex	02011
N, *N'*-Diisopropylcarbodiimide (DIC)	Chem Impex	00110
Ethyl Cyano(hydroxyimino)acetate (Oxyma)	TCI Chemicals	E0847
N, N-Dimethylformamide (DMF) ACS-grade	Millipore Sigma	319937
Dichloromethane ACS-grade	Millipore Sigma	D65100
Methanol ACS-grade and HPLC-grade	Millipore Sigma	179337, 34860
Acetonitrile ACS-grade and HPLC-grade	Millipore Sigma	360457, 34851
Piperidine	Millipore Sigma	104094
Trifluoroacetic acid (TFA) HPLC grade	Millipore Sigma	302031
Trifluoroacetic acid (TFA) reagent-grade	Chem Impex	00289
Dibenzocyclooctyne- DBCO-NHS ester	Broadpharm	BP-22231

Ammonium Bicarbonate	Fisher Scientific	A643-500
Ellman’s Reagent (5,5-dithio-bis-(2-nitrobenzoic acid))	Thermo Scientific	22582
1,4-Bis(bromomethyl)benzene	Ambeed	A575067
TIPS		
**General Materials and Equipment**		
96-well clear conical bottom deep well plates	VWR	76210-524
96-well clear untreated round bottom plates	VWR	82050-622
96-well black half area black flat bottom plates	Millipore Sigma	CLS3694
Amicon® Ultra Centrifugal Filter, 3 kDa MWCO	Millipore Sigma	UFC5003

BIOLOGICAL METHODS

Mycobacteria cell culture. *Mycobacterium smegmatis* strain mc^{2}155 *(Msm)* was grown in 7H9 media with 0.5% glycerol, 0.05% tween 80, and 1x ADC enrichment (10x ADC was made with 5 g bovine serum albumin, 2 g dextrose, 3 mg catalase in 100 mL autoclaved milliQ H_2O). Glycerol stocks were made using stationary phase cells in 30% glycerol and aliquots were stored at - 80 ºC. *Mycobacterium tuberculosis (Mtb)…*

DBCO-tetrapeptide (TetD) based labeling of *Msm*. *Msm* cells were inoculated from the glycerol stock into the 7H9 media in standard culture tubes and grown for 24 hours at 37 ºC until they reached an OD of 0.5-0.6. **TetD** probe stock solution was then added to the media to yield a final concentration of 25 µM and the cells were grown overnight until stationary phase. Notably, blank cells (-DBCO) remained untreated in comparison to the labeled cells at this point. The cells were harvested the next day and centrifuged for 2 min at 3000×g, washed with phosphate-buffered saline containing 0.05% tween 80 (PBST) twice and resuspended in PBST to yield **TetD**-modified *Msm*. To a 96-well plate, 90 µL of labeled cells were added in triplicate. For the blank wells, 90 µL of unlabeled cells were added in triplicate. 10 µL of 500 µM **Fl-az** (50 µM final) was then added to each well followed by incubation at 37 ºC for 1 h. For the experiment testing different azido-fluorophores, 10 µL of 500 µM **Fl-az**, 3-azido-7-hydroxy coumarin (**Co-az)**, azido-tetramethyl rhodamine (**TMR-az**), azido-sulfo cy3 (**sCy3-az**) and azido-sulfo cy5 (**sCy5-az**) were added to **TetD**-treated *Msm* cells. The cells were then centrifuged down, and the pellets were washed with 100 µL PBST twice and fixed with 100 µL 4% formaldehyde for 15 min. The samples were then analyzed by AttuneTM NxT Acoustic Focusing Cytometer.

DBCO-tetrapeptide (TetD) based labeling of *Mtb*. Early log-phase *Mtb* (OD600 0.1) was incubated with +/- 25 µM TetD for 72 hours, then washed twice with PBSTrB (PBS supplemented with 0.05% Triton-X-1002, 0.01% BSA) and treated with for 1 h with 50 µM Fl-az in 7H9 medium. Cells were then washed three times with PBSTrB, fixed with fresh prepared 4% PFA for 2 hours at room temperature, washed twice with PBSTrB, and resuspended in PBS for flow cytometry analysis.

Permeability assay (PAC-MAN) across the *Msm* mycomembrane. To a 96-well plate, 90 µL of **TetD**-modified *Msm* cells were added in triplicate. Next, 5 µL of 1 mM peptide stock solutions were added to yield a final concentration of 50 µM. The volume in each well was made up to 100 µL using PBS. To the blank and PBS control wells, 10 µL of PBS was added at this point. For the blank wells, 90 µL of unlabeled cells were added in triplicate. The plates were then incubated at 37 ºC for 2h. The cells were centrifuged for 2 min at 3000×g, and the supernatant was discarded. The pellets were then washed with 100 µL PBST twice and finally resuspended in 90 µL of PBST. 10 µL of 500 µM **Fl-az** (50 µM final) was then added to each well followed by incubation at 37 ºC for 1h. The cells were then centrifuged again, and the pellets were washed with 100 µL PBST twice and fixed with 100 µL 4% formaldehyde for 15 min. The samples were then analyzed by AttuneTM NxT Acoustic Focusing Cytometer.

Permeability assay (PAC-MAN) across the *Mtb* mycomembrane. Early log-phase *Mtb* (OD600 0.1) was incubated with +/- 25 µM TetD for 36 hours, then washed twice with PBSTrB and treated with the azide-modified test molecules 50 µM in 7H9 medium for 2 h in shaking at 37°C. Cells were then centrifuged to remove the test molecules and treated with Fl-az 50 µM in 7H9 medium for 1 h in shaking at 37°C. Cells were then washed three times with PBSTrB, fixed with fresh

prepared 4% PFA for 2 h, washed twice with PBSTrB, and resuspended in PBS for flow cytometry analysis.

Isolation of peptidoglycan and muropeptides. *Msm* cells were inoculated from the glycerol stock into the 7H9 media in standard culture tubes and grown for 24 hours at 37 ºC until they reached an OD of 0.5-0.6. **TetD** probe stock solution was then added to the media to yield a final concentration of 25 µM and the cells were grown overnight until stationary phase. The cells were harvested the next day and centrifuged for 2 min at 3000×g, washed with PBST, phosphate-buffered saline PBST twice and resuspended in PBST to yield **TetD**-modified *Msm*. To a 96-well plate, 90 µL of labeled cells were added in triplicate. 10 µL of 500µM **Fl-az** (50 µM final) was then added to each well followed by incubation at 37 °C for 1 h. The cells were then centrifuged down, and the pellets were washed with 100 µL PBST twice. The cell pellets were then resuspended in 200 µL 10 mM NH_4HCO_3 containing protease inhibitor and sonicated in an ultrasonic bath sonicator for 30 min. 10 µg/mL each of DNase and RNase were then added to each well directly after sonication and the plate was placed in 4 °C for 1 h. The cell wall-enriched fraction was then collected by centrifugation at 2700 g for 10 min. The pellets were treated with 200 µL PBS containing 2% sodium dodecyl sulfate (SDS) at 50 °C for 1 h with shaking at 250 rpm. The suspension was spun down at 2700 g for 10 min. This treatment was repeated twice. Then the resulting pellet was resuspended in 200 µL PBS containing 1% SDS and 0.1 mg/ml proteinase K each well, incubated at 37 °C for 1 h with shaking at 250 rpm. The suspension was then heated in boiling water for 1 h and spun down at 2700 g for 10 min. The supernatant was discarded and the 1% SDS extraction step was repeated twice. The pellet was then washed twice with PBS and four times with deionized water to give the mycolyl-arabinogalactan-peptidoglycan Complex (MAPc). The MAPc samples were then resuspended in 200 µL methanol containing 0.5% KOH and incubated at 37 °C for 4 days with shaking at 250 rpm. The mixture was then washed twice with methanol twice and twice with diethyl ether and air-dried to give arabinogalactan-peptidoglycan (AGPG). The resulting AGPG was resuspended in 200 µL deionized water and digested with 0.05 N H_2SO_4 at 37 °C for 5 days with shaking at 250 rpm. The samples were then washed four times with deionized water to give insoluble peptidoglycan (PG). The isolated sacculi from each well in each group was then combined and lyophilized, respectively. The lyophilized sacculi was resuspended in 0.4 mL 10 mM sodium acetate (pH 5) containing 25 µg/mL mutanolysin and lysozyme and digested at 37 °C over 20 h. The mixture was then spun down, and the supernatant was filtered with 10 kDa MWCO and lyophilized. The lyophilized muropeptide samples were then dissolved in 30 µL MilliQ water and an aliquot was analyzed *via* a C18(2) column (Luna 5 µm 100Å 250 x 4.6 mm) on an Agilent LC-QTOF system (Agilent 1260 Infinity II Prime LC with Agilent 6545B QTOF). Muropeptide samples were eluted with a 2 to 30% linear gradient of H_2O/CH_3CN (0.5% formic acid) at 0.4 mL/min. The muropeptides were analyzed by MS using MassHunter software.

Ethidium bromide (EtBr) whole-cell accumulation assay. To a 96-well plate, 90 µL of **TetD** -modified *M. smegmatis* cells were added in triplicate. Next, 5 µL of 1 mM peptide stock solutions were added to yield a final concentration of 50 µM. The volume in each well was made up to 100 µL using PBS. To the blank and PBS control wells, 10 µL of PBS was added at this point. For the blank wells, 90 µL of unlabeled cells were added in triplicate. The plates were then incubated at 37ºC for 2h. The cells were centrifuged for 2 min at 3000× g, and the supernatant was discarded. The pellets were then washed with 100 µL PBST twice and finally resuspended in 100 µL of PBST. 95 µL of these cells were then transferred to corresponding wells in a 96-well, black, flat-bottomed plate. To each well, 5 µL of 100 µM EtBr (5 µM final) was added. The fluorescent

intensities were measured by a Biotek Synergy H1 microplate reader 60 m post-treatment with EtBr, with continuous orbital shaking. Wavelengths: ethidium bromide, excitation 530 nm/emission 590 nm.

DBCO-modified polystyrene bead reactivity assay. For functionalization with DBCO, 100 µL of amino functionalized polystyrene beads (5% w/v, 5 mg) were centrifuged at 21000 g for 10 min in a 1.7 mL eppendorf tube and washed with 1 mL deionized water. The beads were then resuspended in 1 mL 20 mM sodium borate buffer (pH 9) containing 1 µg/mL DBCO-NHS and incubated at 37 °C for 2 h with shaking. The resulting beads were centrifuged at 21000 g for 10 min and then washed and resuspended in sodium borate buffer. For capping the remaining, unreacted amine sites on the beads, 20 µL of acetic anhydride was added to the bead suspension and the beads were then incubated at 37 °C for 2 h with shaking. The resulting beads were then centrifuged and washed twice with 1 mL PBS and resuspended in 1 mL PBS for further use. For the reactivity assay, the DBCO modified polystyrene beads were diluted 1:1 in PBS prior to the assay. A 96-well plate was loaded with 95 µL of test molecules (for final concentration of 50 µM) to which 5 µL of the beads was added to each well. The plate was incubated in 37 °C for 2 h and centrifuged. The supernatant was decanted and 100 µL 50 µM Fl-az was added to each well. The plate was then incubated at 37 °C for 1h and centrifuged at 2700 g for 10 min. The supernatant was decanted, and the beads were washed with 200 µL PBS and then resuspended in 200 µL PBS. The samples were then analyzed by AttuneTM NxT Acoustic Focusing Cytometer.

Calculation of physiochemical properties of test molecules. DataWarrior software was used to calculate the molecular weight (MW), number of hydrogen bond donors (HBDs), number of hydrogen bond acceptors (HBAs), water/octanol partitioning coefficient (cLogP), polar surface area (PSA), and number of rotatable bonds (nRotBs) for each peptide.

Hydrogen-deuterium exchange (HDX) studies. NMR Data was acquired on either Bruker Ascend 400 MHz or Varian 600 MHz spectrophotometers. For both peptides, fresh solutions were made in deuterated solvents, namely, 70:30 [CD_3CN:D_2O] at ~10 mM concentration, prior to NMR studies. First water suppression 1H-NMR was acquired *via* a pre-saturation experiment using the Bruker Ascend 400 MHz spectrophotometer. Once the parameters for water pre-saturation were set, using the same parameters, all 2D-NMR (COSY, TOCSY, ROSEY) and 1D-NOESY spectra were acquired for the detailed assignment of each proton (especially amide NH) and its correlation with neighboring carbons and protons. NMR data was processed and analyzed using the software MestreNova. Residual solvent signals from deuterated solvents were used as an internal standard with reference to tetramethylsilane (TMS) for defining chemical shifts. Chemical shifts are reported in ppm (δ) and coupling constants (J) are reported in Hertz [Hz]. Time dependent spectra were continuously acquired after inserting sample into the instrument followed by locking and shimming, ~5 minutes past making fresh solutions. Area under the curve (integration) of each individual *NH* was used for determining the exchange rate, the initial integrations of these protons were normalized to non-exchangeable aromatic protons of the phenylalanine residue and were followed with time for reduced intensity by *N-H* to *N-D* exchange.

Molecular dynamics (MD) simulation studies. Three replicates of MD simulations were conducted for each of the lin1 and cyc1 peptide systems to compare their self-interactions. Each peptide was solvated using the TIP3P water model[1] and 0.15 M KCl was added to the system. The systems were prepared using the CHARMM scripts provided by CHARMM-GUI.[2] The CHARMM36m force field[3] was employed for the MD simulations. After energy minimization and 500 ps equilibration, a 100 ns production run was performed. All production MD simulations were performed for the NPT ensemble at 303.15 K and 1 atm using NAMD3. Long-range electrostatic

interactions were treated using the particle mesh Ewald (PME) method[4] while Lennard-Jones (LJ) interactions were managed with a force-based switching function applied between 10 Å and 12 Å. Pressure and temperature were maintained using the Nosé–Hoover Langevin-piston method[5] with a piston period of 50 fs and a piston decay of 25 fs, coupled with Langevin temperature control with a friction coefficient of 1 ps^{-1}. A time step of 1 fs was chosen to capture detailed peptide dynamics. Hydrogen bonds between different residues within the same peptide were analyzed using the last 50 ns of the simulation.

Metabolic stability assay. Time course degradation in mouse serum was evaluated for peptides lin1, cyc1, Nmet1 and Nmet10. Compounds were incubated in mouse serum at a concentration of 1.0 mM to a total of 200 μL at 37 °C. At each time point (0, 15, 30, 60 mins), 40 μL of the solution was aliquoted and diluted in a 1:1 solution of H_2O/CH_3CN (101 μL) containing trichloroacetic acid (9 μL) and 100 μM of internal standard Fmoc-L-phenylalanine. This mixture was incubated in ice for 5 min to precipitate serum proteins. The samples were then centrifuged at 21,000×g for 5 min. 140 μL of the supernatant was then transferred to a filter tube and centrifuged again at 21,000×g for 5 min to filter. 110 μL of the filtrate was then analyzed using Phenomenex Luna 5 μm C8(2) on RP-HPLC; gradient elution in H_2O/CH_3CN with 0.1% TFA at 1 mL/min. Peptide identity was confirmed via peak collection and subsequent matrix-assisted laser desorption ionization time-of-flight (MALDI-TOF) mass spectroscopy (Shimadzu 8020). To eliminate variations in the overall signal intensity, area under the curve (AUC) for the peptides of interest at each time point was normalized to the AUC of the internal standard Fmoc-L-phenylalanine, the concentration of which was predetermined. These normalized AUC values were then further normalized to the "0 min" time point which was set to 100%. Percentage peptide remaining was then plotted as a function of time.

Minimum inhibitory concentration (MIC) assay.

Msm cells were inoculated from the glycerol stock to the according media and grown for 24 hours until 0.5-0.6 OD. Testing peptides were dissolved in the 7H9 growth media mentioned above and made into serial dilutions from 128 μM. 200 μL of each concentration was added to a deep well 96-well plate – with each concentration added in triplicates. The cells were diluted 100 times to OD of around 0.05 with fresh 7H9 growth media. 200 mL of diluted cells were added to the deep well plate each well and mixed well with the peptide solution. The plate was covered by an adhesive porous film and incubated at 37 °C with shaking at 250 rpm for 48 h. The contents from each well were then transferred to a flat-bottom 96-well plate for MIC value determination. MIC values were determined both visually and readings of OD from a Biotek Synergy H1 microplate reader at 600 nm.

Synthesis and Characterization of test molecules

General procedure for the solid-phase synthesis of all peptides. All peptides were prepared by standard Fmoc-based solid-phase chemistry using the appropriate resins. Briefly, to a 25 mL peptide synthesis vessel, appropriate amount of resin was added, followed by 20% piperidine in *N, N*-Dimethylformamide (DMF, 15 mL). This was followed by shaking at room temperature for 30 min. The resin was then washed with methanol (CH_3OH) and dichloromethane (DCM) three times. After the last wash, 4 eqiv. of amino acid was added along with 4 eqiv. of ethyl cyanohydroxyiminoacetate (Oxyma) and 4 eqiv. of *N, N'*-Diisopropylcarbodiimide (DIC). The resin was shaken at room temperature for 2 h, then washed with CH_3OH/DCM. The remainder of the amino acids were coupled in the same manner. Peptides were cleaved from the resin using a TFA/TIPS/H_2O mixture (95:2.5:2.5, v/v/v) (unless otherwise mentioned) with shaking at room temperature for 2 h. The solution was filtered and concentrated prior to precipitation by the addition of cold diethyl ether to yield crude peptide. Crude peptides were purified by reverse-phased preparative high-performance liquid chromatography (RP-HPLC) equipped with Waters 1525 with 2489 UV/Visible Detector on a Phenomenex Luna 10 µm C8(2) 100 Å (250 x 21.2 mm) column using gradient elution with H_2O/MeOH with 0.1% TFA at 10 mL/min. The HPLC fractions of the desired purified compounds were first concentrated under reduced pressure using a rotary evaporator. The final concentrated aqueous solutions were lyophilized to dryness using Labconco Freezone 4.5 L (-84°C) lyophilizer. The peptides were analyzed for purity using Phenomenex Luna 5 µm C8(2) on the same RP-HPLC; gradient elution in H_2O/MeOH with 0.1% TFA at 1 mL/min. Peptide identities were confirmed via high resolution electrospray ionization mass spectrometry (HRMS, ESI/MS). Analyses were obtained on an Agilent 6545B Q-TOF LC/MS equipped with 1260 infinity II LC system with auto sampler. Samples were dissolved in CH_3CN and eluted with a CH_3CN/H_2O solution containing 0.1% formic acid

Synthesis of TetD. To prepare **TetD**, 2-Chlorotrityl chloride resin (0.142 mmol) was used such that the *C*-terminus would be a carboxylic acid upon cleavage. Synthesis was performed in accordance with established procedures for that resin. For the coupling of DBCO to the *N*-terminus of the peptide, 0.9 equiv. of DBCO-NHS was dissolved in 1 mL of dry DMF and added to the *N*-terminally deprotected peptide on resin resuspended in 2 mL of DMF. The coupling was carried out overnight at room temperature. Cleavage from the resin was then carried by the addition of 20% trifluoroacetic acid (TFA) in dichloromethane with subsequent shaking in room temperature for 1 h. The liquid phase was then filtered, concentrated and precipitated to yield crude peptide as described. The crude peptide was purified via HPLC as described in the general procedure. The **TetD** stock was characterized using UV-Vis absorbance at 309 nm (ε = 12,000 $cm^{-1}M^{-1}$).

Synthesis of the macrocyclic peptide library. To prepare peptides in this library, Rink-amide resin (0.48 mmol/g of loading capacity) was used such that the *C*-terminus would be amidated upon cleavage. Synthesis was performed in accordance with established procedures for the resin. For all linear analogs within the library, the *N*-terminus was acetylated by combining the peptide with a mixture of acetic anhydride, DIEA, and DMF (5:8.5:86.5, v/v/v) for 1 hour at room temperature. For the cyclization chemistry of the Cyc (0-4) and Lar (1-5) series, 10 equiv. of chloroacetic chloride and 15 equiv. of DIEA were dissolved in anhydrous DCM and coupled to the *N*-terminally deprotected peptide by shaking it for 2 hours at room temperature. Cleavage from resin was performed as described above. For the linear analogs, purification was then subsequently carried out as described above. For the macrocyclic peptides Cyc (0-4) and Lar (1-

5), the cleaved, crude peptide scaffolds were then dissolved in a mixture of H_2O/CH_3CN (1:1, v/v) to an approximate concentration of ~1.5 mM. This was followed by the addition of 0.5 M NH_4HCO_3 (pH 8.5) to a final concentration of 50 mM to form the thioether bonds for macrocyclization. The cyclization reaction mixture was then stirred overnight at room temperature (~16h). Cyclization was monitored using matrix-assisted laser desorption ionization time-of-flight (MALDI-TOF) mass spectroscopy (Shimadzu 8020). For the macrocyclization of peptide Dit1, the crude peptide scaffold was dissolved in a mixture of H_2O/CH_3CN (1:1, v/v) to an approximate concentration of ~1.5 mM. This was followed by the addition of 0.5 M NH_4HCO_3 (pH 8.5) to a final concentration of 50 mM to form the disulfide bond required for macrocyclization. The cyclization reaction mixture was then stirred overnight at room temperature (~16h). Cyclization was assessed by monitoring the presence of free thiols using Ellman's reagent as per the standard protocol provided by the manufacturer. For the macrocyclization Dit2, the crude peptide scaffold was dissolved in a mixture of H_2O/CH_3CN (1:1, v/v) to an approximate concentration of ~1.5 mM. Further, the bis-electrophilic cyclization linker 1,4-Bis(bromomethyl)benzene was also dissolved into this mixture to an approximate concentration of ~1.5 mM (1 equiv.). Thioether bond formation between the Dit2 peptide scaffold and the linker was brought about by the addition of 0.5 M NH_4HCO_3 (pH 8.5) to a final concentration of 50 mM. The cyclization reaction mixture was then stirred overnight at room temperature (~16h). Cyclization was monitored using matrix-assisted laser desorption ionization time-of-flight (MALDI-TOF) mass spectroscopy (Shimadzu 8020). For all the above-mentioned macrocyclic peptides, upon confirmation of macrocyclization, the samples were then concentrated under reduced pressure using a rotary evaporator. The concentrated aqueous solutions were then lyophilized to dryness using a Labconco Freezone 4.5 L (-84°C) lyophilizer. Purification was subsequently carried out as described above. All peptides in this library were characterized using UV-Vis absorbance of the phenylalanine residue in their sequence at 257 nm (ε = 195 $cm^{-1}M^{-1}$). For Dit2, the extinction coefficient at 257 nm of 1,4-Bis(bromomethyl)benzene was also determined separately using a calibration curve and accounted for during concentration determination of Dit2.

Synthesis of the *N*-alkylated peptide library. To prepare peptides in this library, Rink-amide resin (0.48 mmol/g of loading capacity) was used such that the *C*-terminus would be amidated upon cleavage. Synthesis was performed in accordance with established procedures for the resin. *N*-methyl amino acids were used for the *N*-methyl analogues in the peptide library. Azido-acetic acid was coupled on the *N*-terminus of each peptide on resin. For the peptoid library, the *N*-alkyl substituted glycine residues in the peptoids were added as per reported protocols. Briefly, to a 25 mL peptide synthesis vessel containing 100 mg of Rink amide resin (0.048 mmol), conjugated with or without peptide residues, was added 20% piperidine in DMF (15 mL), with shaking at room temperature for 30 min. The resin was then washed with CH_3OH/DCM. After the last wash, to the resin was added 1 mL 0.6 M 2-bromo acetic acid with 200 μL of 50% DIC in DMF. The resin was shaken at room temperature for 20 min, then washed with DMF for 5 times. 1 mL 1.5 M primary amine building blocks were then added to the resin and the resin was shaken at room temperature for 1-2 h. The rest of the amino acids are coupled in the same manner according to the sequence of the peptide. As before, azido-acetic acid was coupled on the *N*-terminus of each peptide/peptoids on resin. Cleavage and purification were carried out as described before. All peptides in this library were characterized using UV-Vis absorbance of the phenylalanine residue in their sequence at 257 nm (ε = 195 $cm^{-1}M^{-1}$).

Synthesis of the tridecaptin series. To prepare peptides in this library, 2-Chlorotrityl chloride resin (0.142 mmol) was used such that the *C*-terminus would remain as a carboxylic acid upon

cleavage. Synthesis was performed in accordance with established procedures for the resin. Notably, n-Octanoic acid was coupled on the *N* terminus of the Tridecaptin peptide derivatives on resin. n-Octanoic acid, (67.5 μL, 3 eq, 0.43 mmol) was dissolved in 5 mL DMF with Oxyma (3.0 eq, 60 mg, 0.43 mmol) and DIC (3.0 eq, 66 μL, 0.43 mmol), and added to the resin. For the macrocyclization Cyc-Oct-tri-A1, the crude peptide scaffold was dissolved in a mixture of H_2O/CH_3CN (1:1, v/v) to an approximate concentration of ~1.5 mM. Further, the bis-electrophilic cyclization linker 1,4-Bis(bromomethyl)benzene was also dissolved into this mixture to an approximate concentration of ~1.5 mM (1 equiv.). Thioether bond formation between the Dit2 peptide scaffold and the linker was brought about by the addition of 0.5 M NH_4HCO_3 (pH 8.5) to a final concentration of 50 mM. The cyclization reaction mixture was then stirred overnight at room temperature (~16h). Cyclization was monitored using matrix-assisted laser desorption ionization time-of-flight (MALDI-TOF) mass spectroscopy (Shimadzu 8020). Upon confirmation of macrocyclization, the sample was then concentrated under reduced pressure using a rotary evaporator. The concentrated aqueous solution was then lyophilized to dryness using a Labconco Freezone 4.5 L (-84°C) lyophilizer. Purification was subsequently carried out as described above.

Characterization of test molecules. The peptides were analyzed for purity using Phenomenex Luna 5 μm C8(2) on the same RP-HPLC; gradient elution in H_2O/MeOH with 0.1% TFA at 1 mL/min. Peptide identities were confirmed via high resolution electrospray ionization mass spectrometry (HRMS, ESI/MS). Analyses were obtained on an Agilent 6545B Q-TOF LC/MS equipped with 1260 infinity II LC system with auto sampler. Samples were dissolved in CH_3CN and eluted with a CH_3CN/H_2O solution containing 0.1% formic acid. Characterization data are provided below.

Lin0

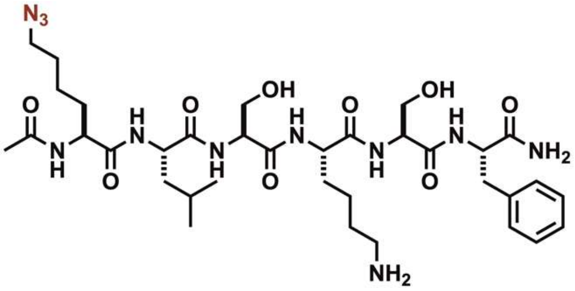

Analytical HPLC Chromatogram of Lin0

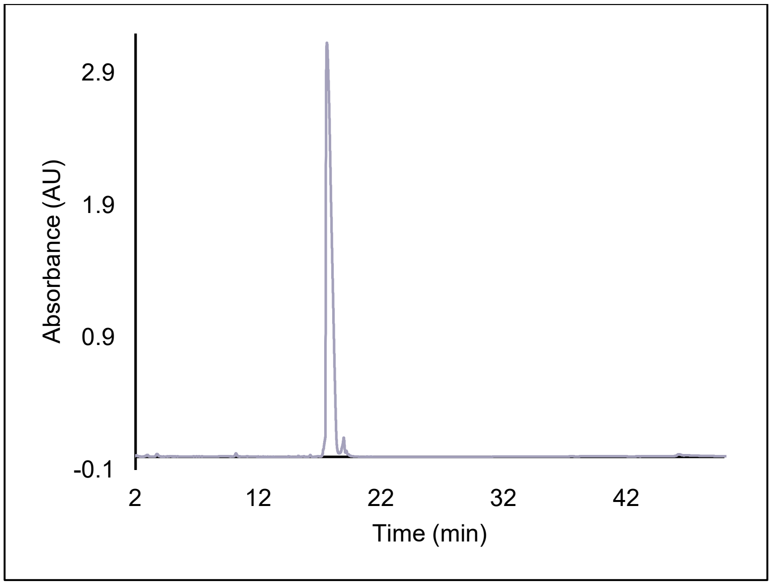

QTOF High Resolution Mass Spectrum for Lin0 (m/z 776.4341 for [M+H]+)

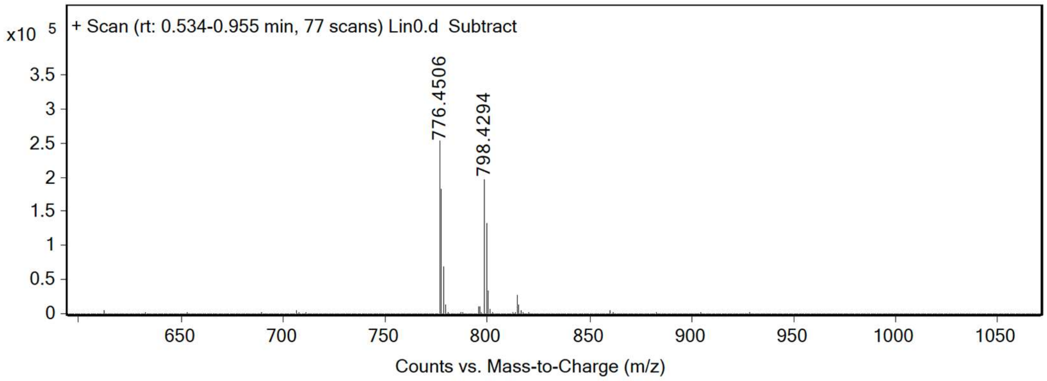

Lin1

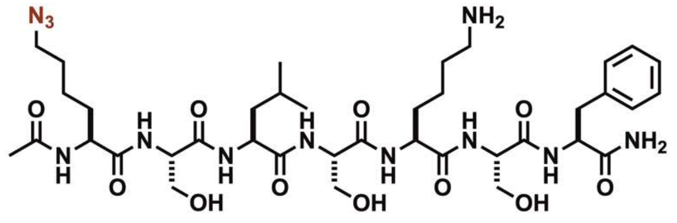

Analytical HPLC Chromatogram of Lin1

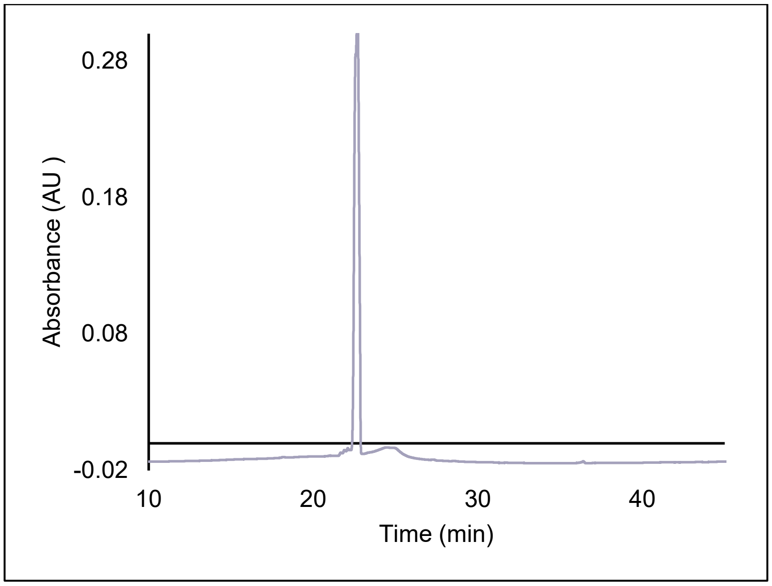

QTOF High Resolution Mass Spectrum for Lin1 (m/z 863.4661 for [M+H]+)

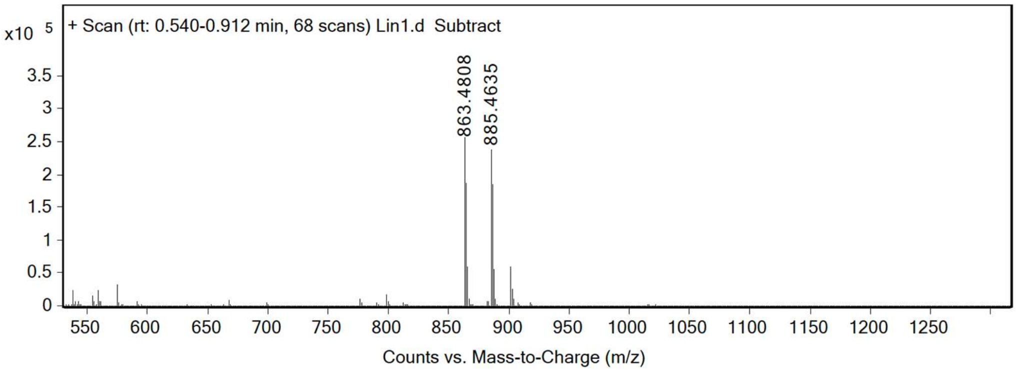

Lin2

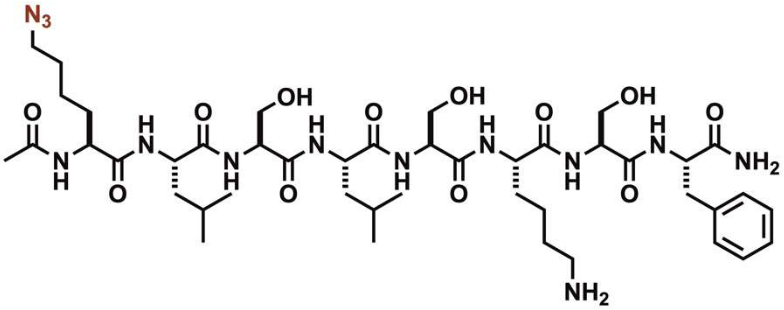

Analytical HPLC Chromatogram of Lin2

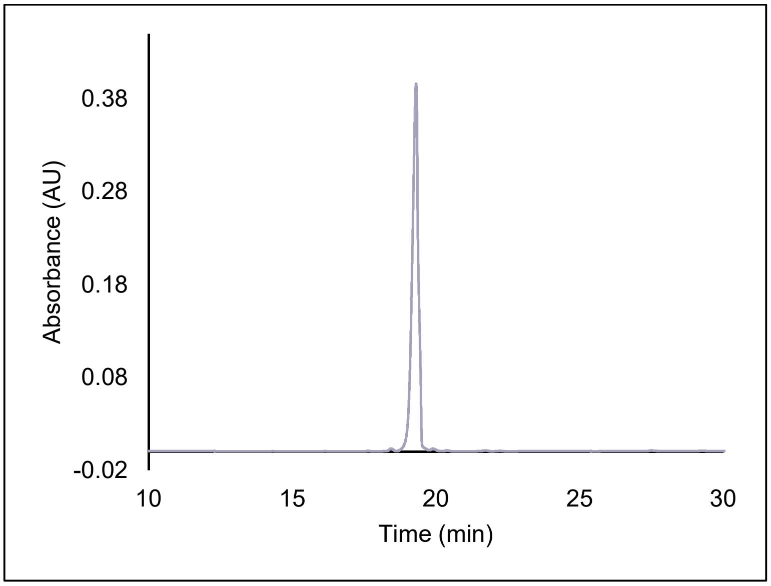

QTOF High Resolution Mass Spectrum for Lin2 (m/z 976.5502 for [M+H]+)

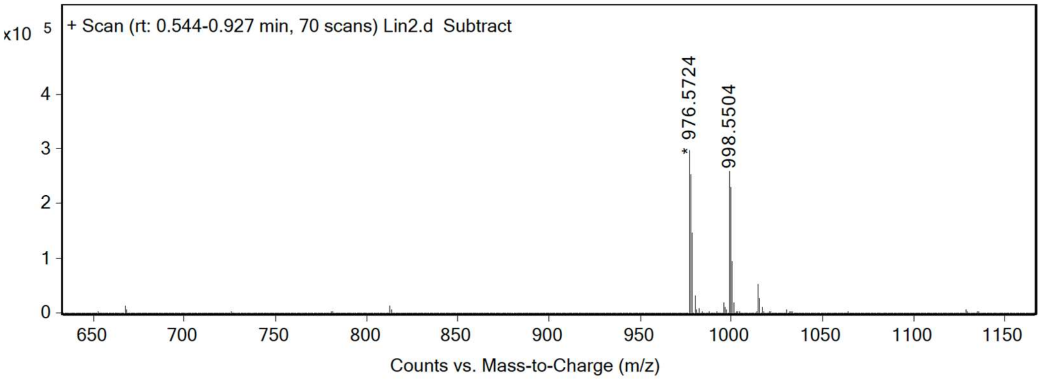

Lin3

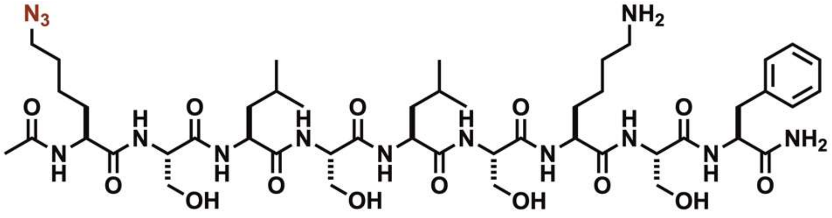

Analytical HPLC Chromatogram of Lin3

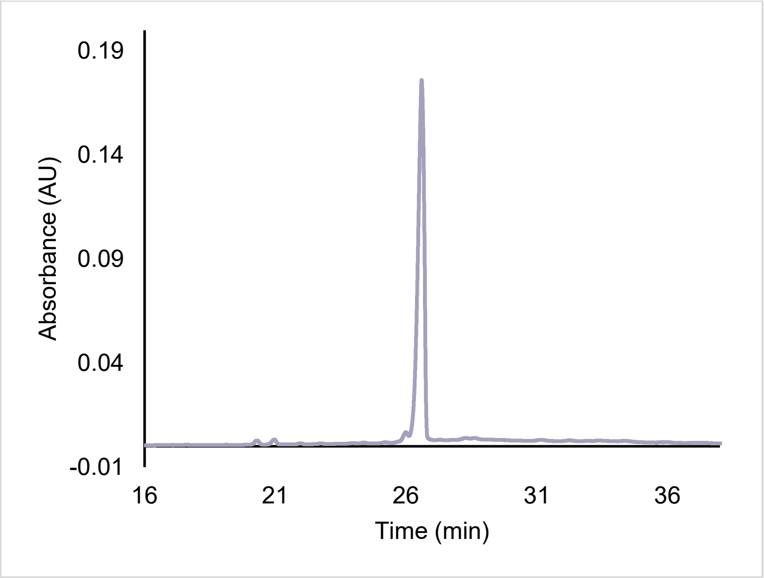

QTOF High Resolution Mass Spectrum for Lin3 (m/z 1063.5822 for [M+H]+)

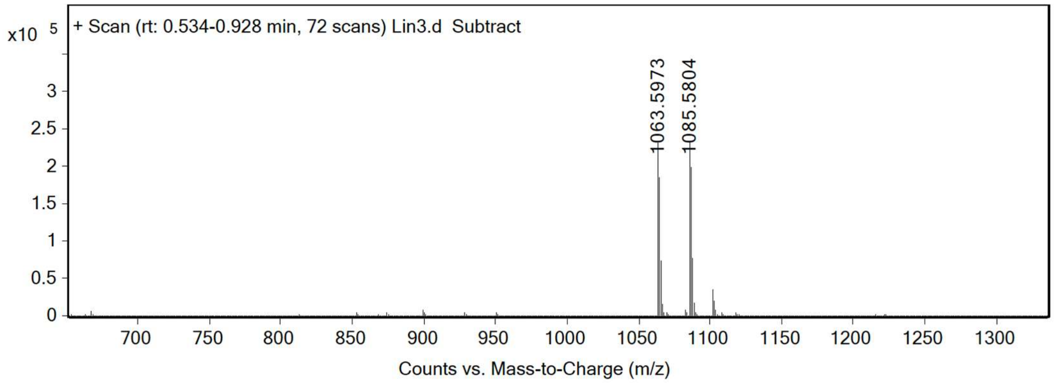

Cyc0

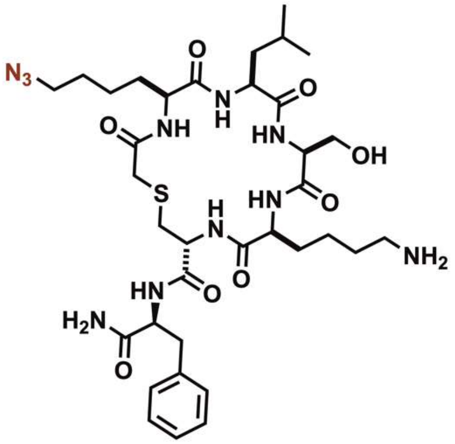

Analytical HPLC Chromatogram of Cyc0

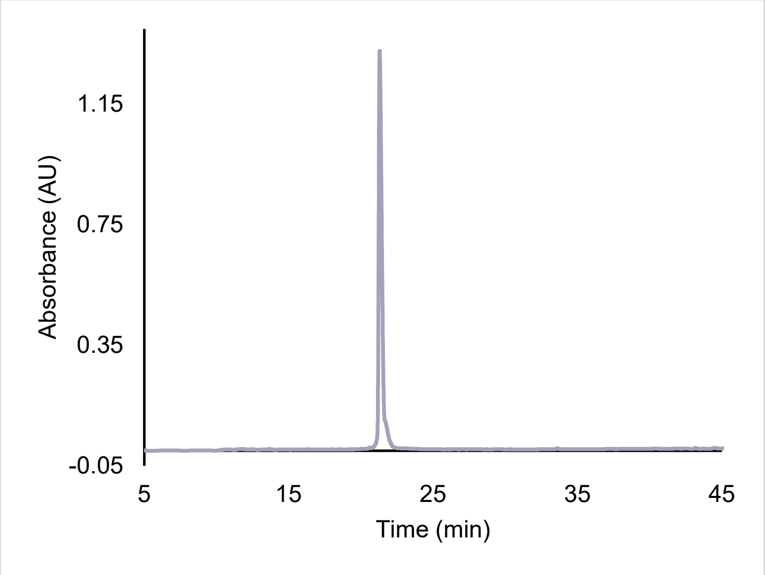

QTOF High Resolution Mass Spectrum for Cyc0 (m/z 790.3956 for [M+H]+)

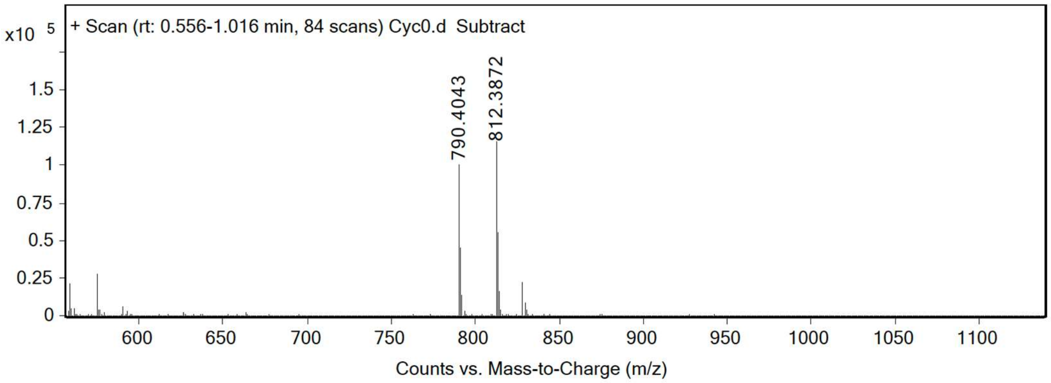

Cyc1

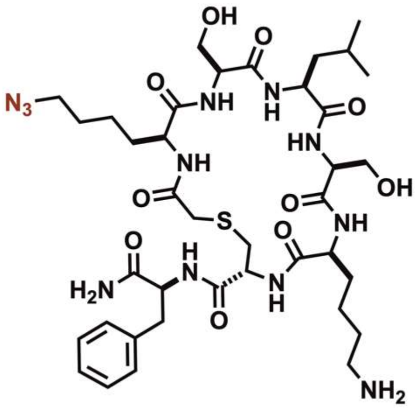

Analytical HPLC Chromatogram of Cyc1

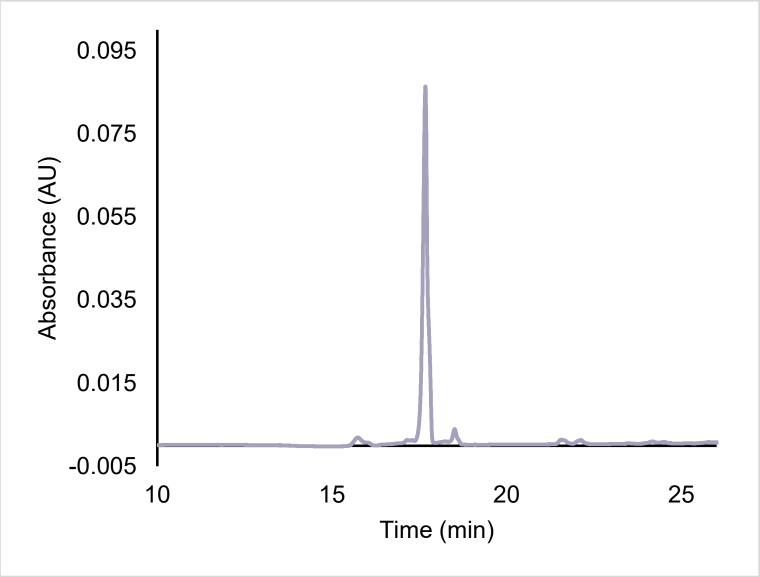

QTOF High Resolution Mass Spectrum for Cyc1 (m/z 877.4276 for [M+H]+)

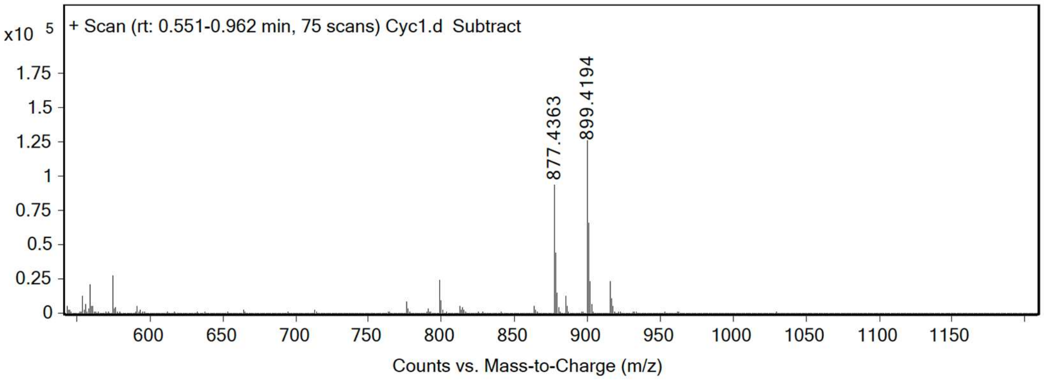

Cyc2

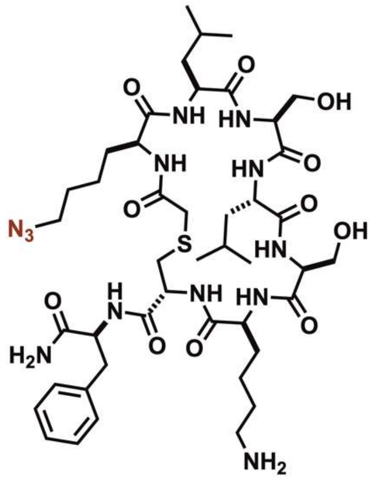

Analytical HPLC Chromatogram of Cyc2

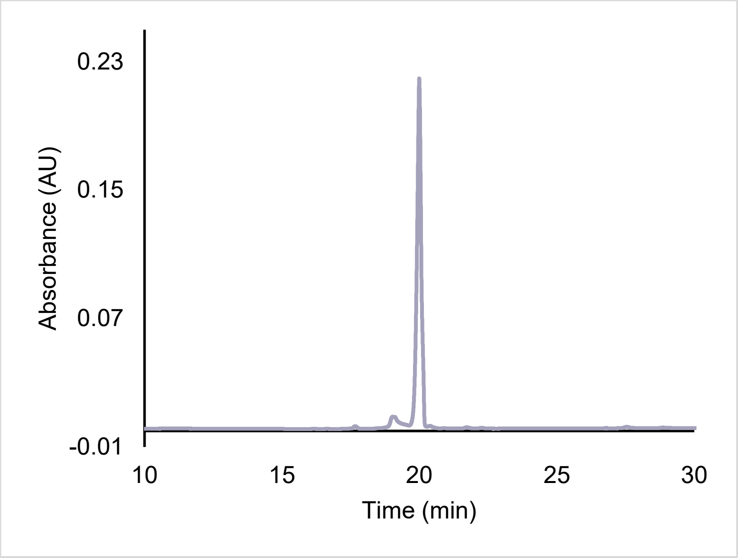

QTOF High Resolution Mass Spectrum for Cyc2 (m/z 990.5117 for [M+H]+)

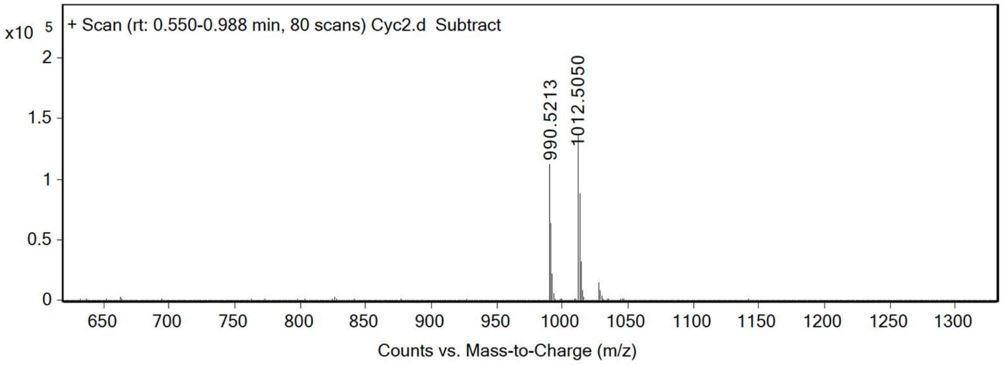

Cyc3

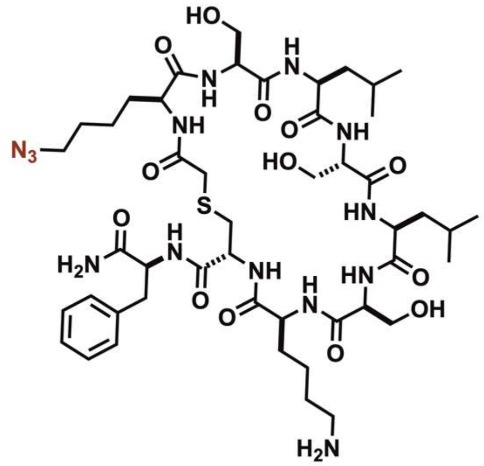

Analytical HPLC Chromatogram of Cyc3

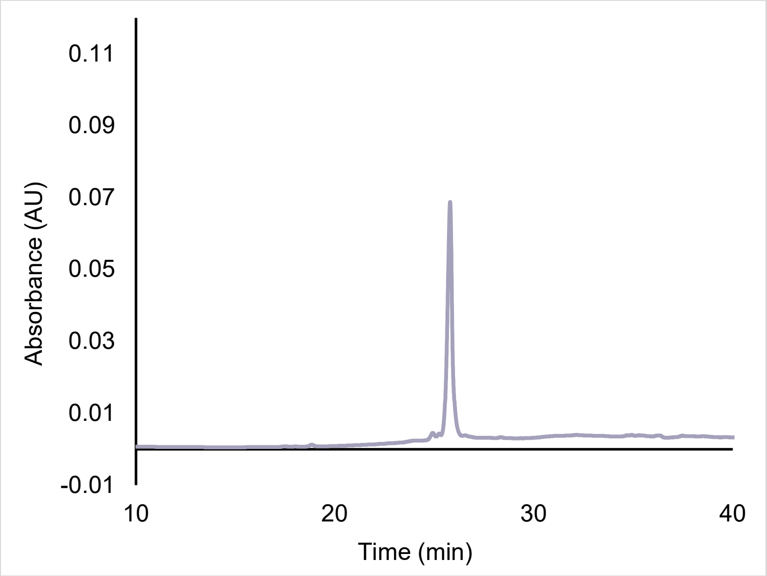

QTOF High Resolution Mass Spectrum for Cyc3 (m/z 1077.5437 for [M+H]+)

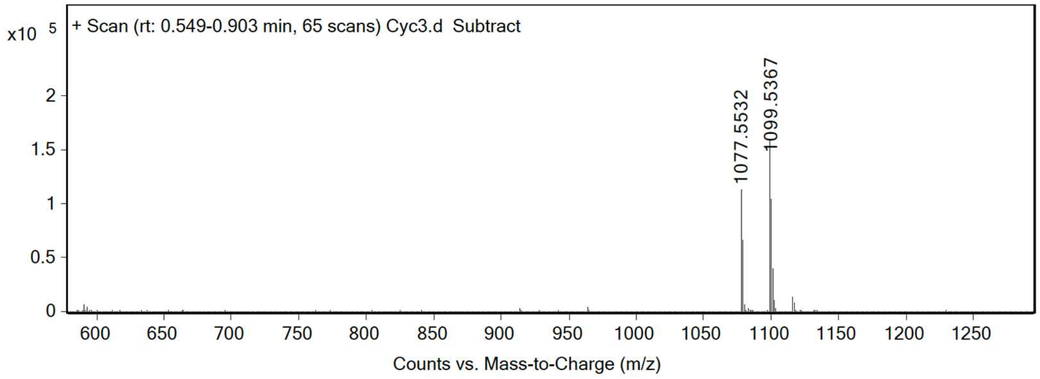

Lar0

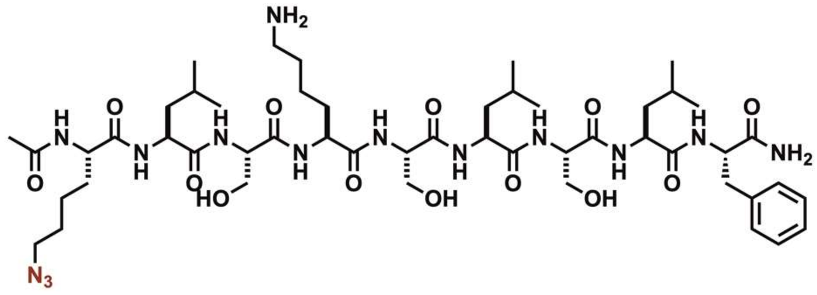

Analytical HPLC Chromatogram of Lar0

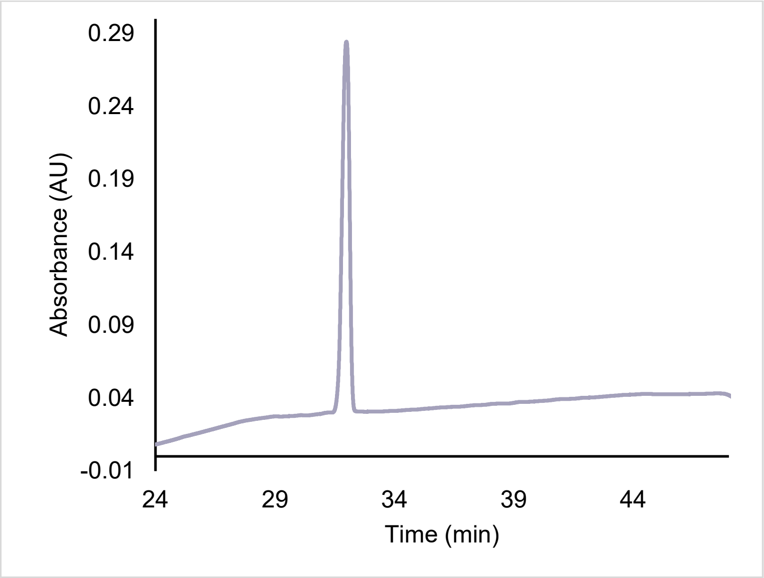

QTOF High Resolution Mass Spectrum for Lar0 (m/z 1089.6342 for [M+H]+)

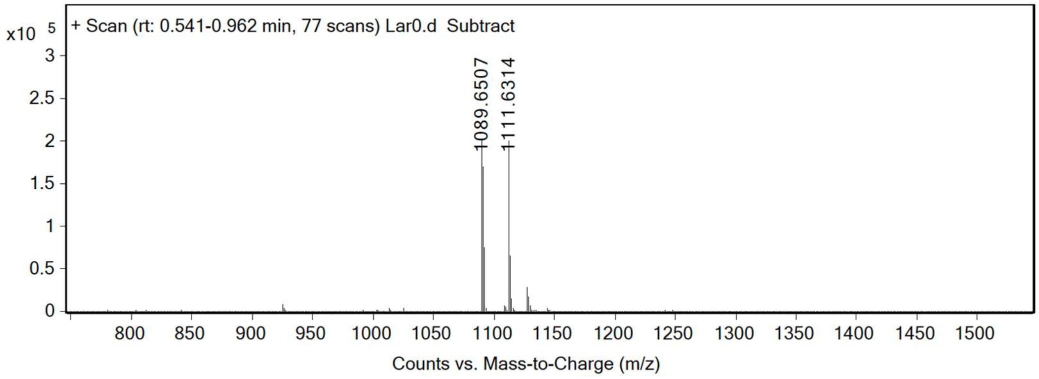

Lar1

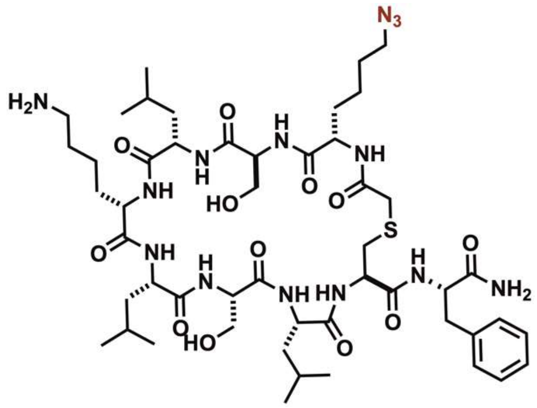

Analytical HPLC Chromatogram of Lar1

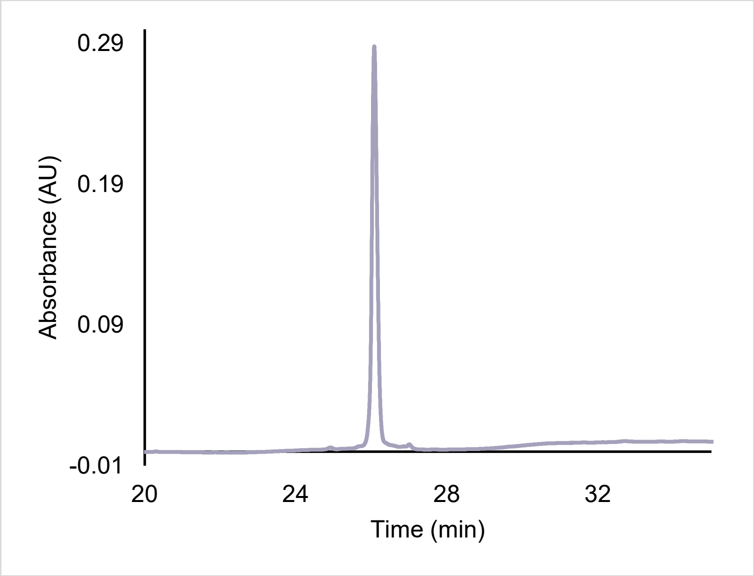

QTOF High Resolution Mass Spectrum for Lar1 (m/z 1089.6342 for [M+H]+)

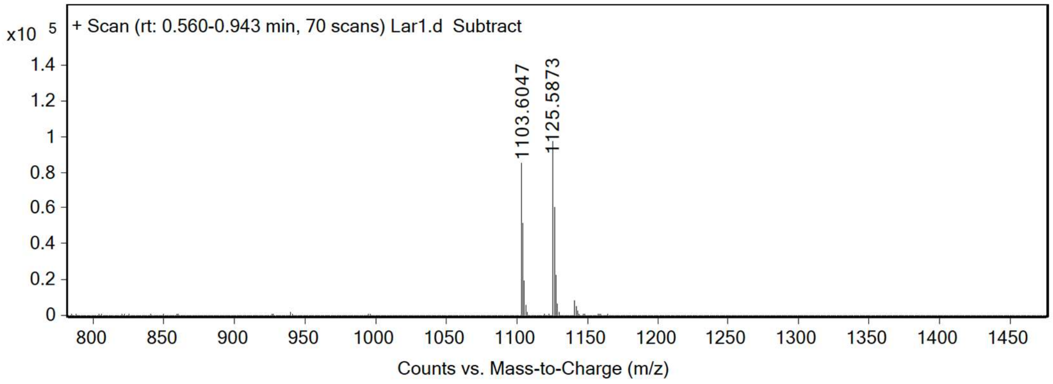

Lar2

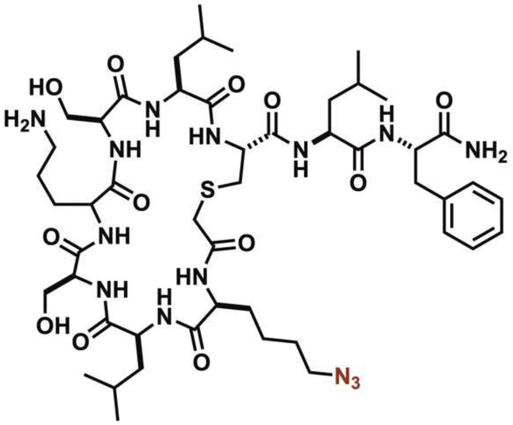

Analytical HPLC Chromatogram of Lar2

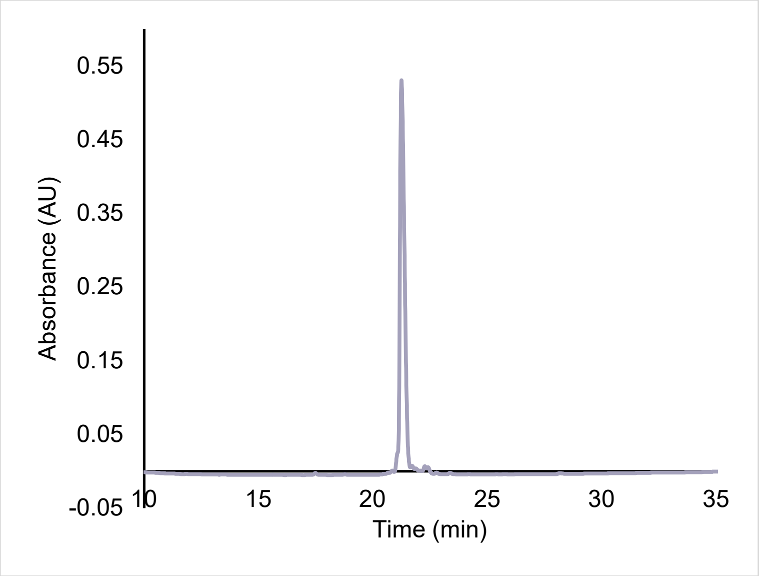

QTOF High Resolution Mass Spectrum for Lar2 (m/z 1103.5957 for [M+H]+)

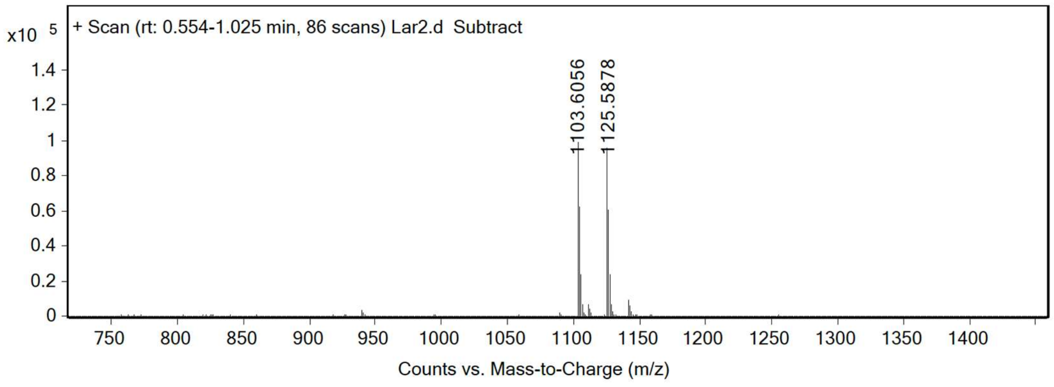

Lar3

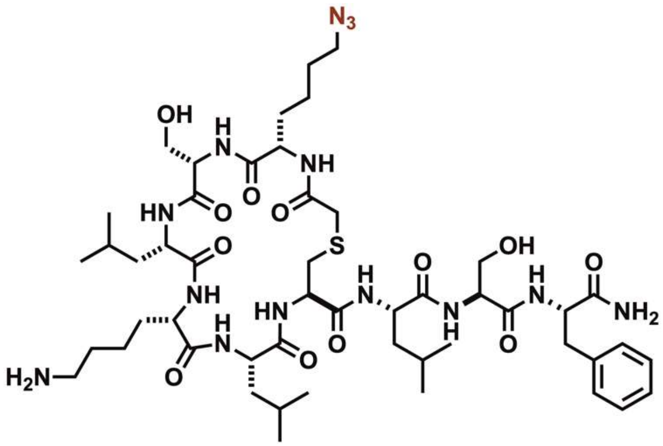

Analytical HPLC Chromatogram of Lar3

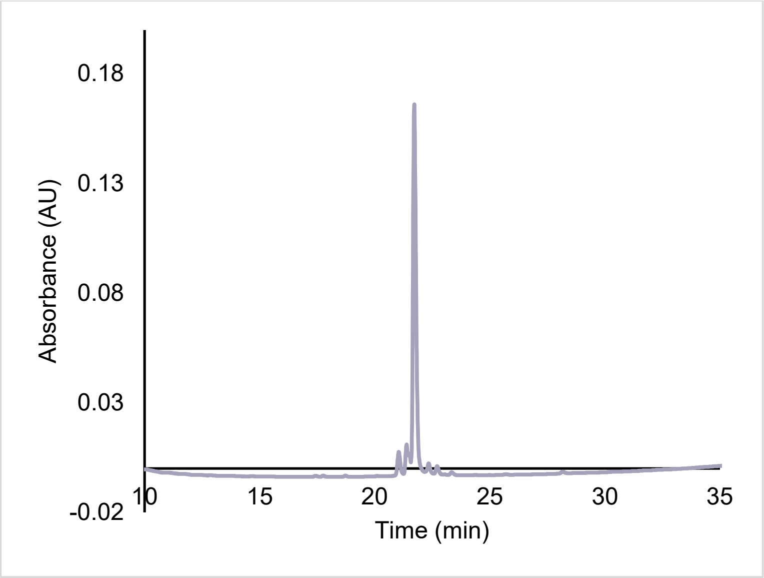

QTOF High Resolution Mass Spectrum for Lar3 (m/z 1103.5957 for [M+H]+)

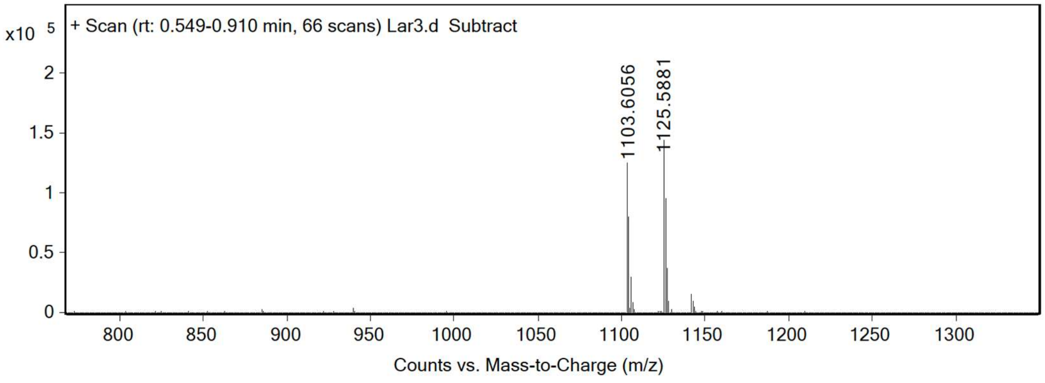

Lar4

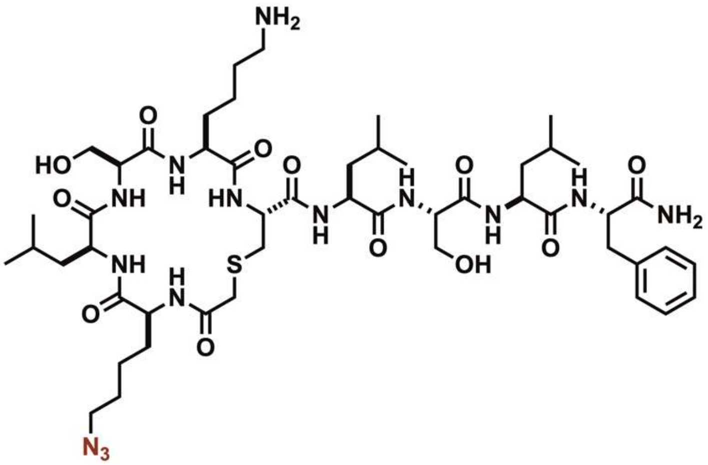

Analytical HPLC Chromatogram of Lar4

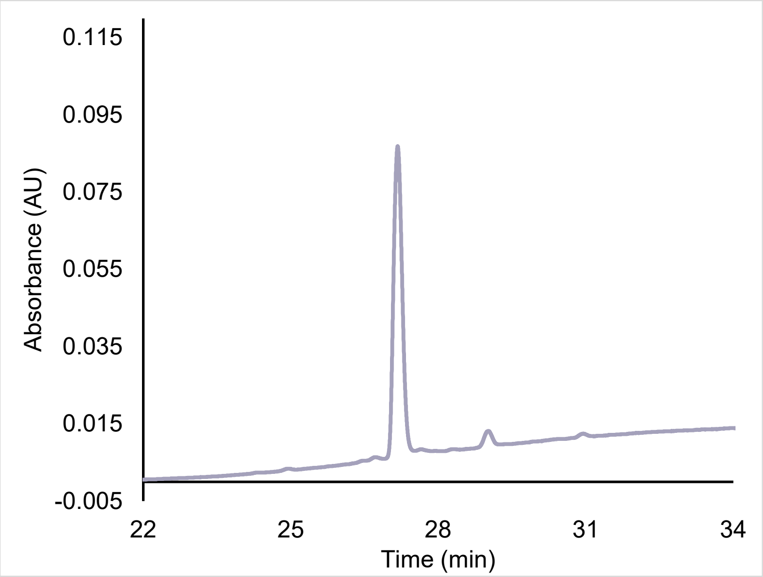

QTOF High Resolution Mass Spectrum for Lar4 (m/z 1103.5957 for [M+H]+)

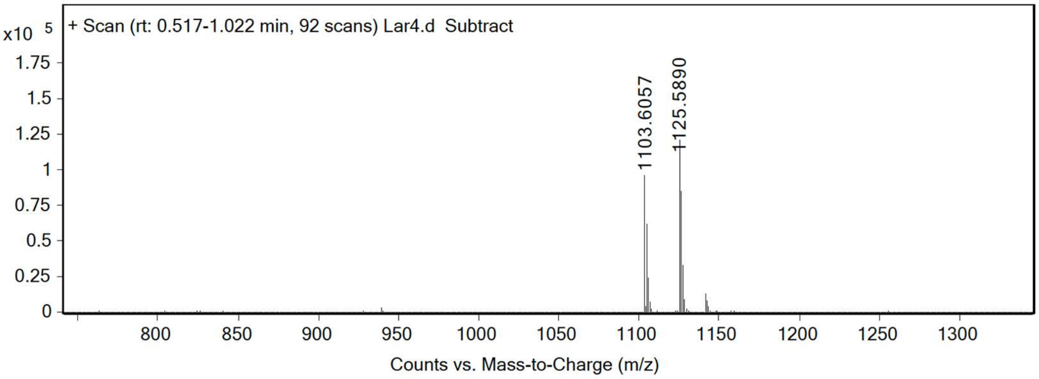

Lar5

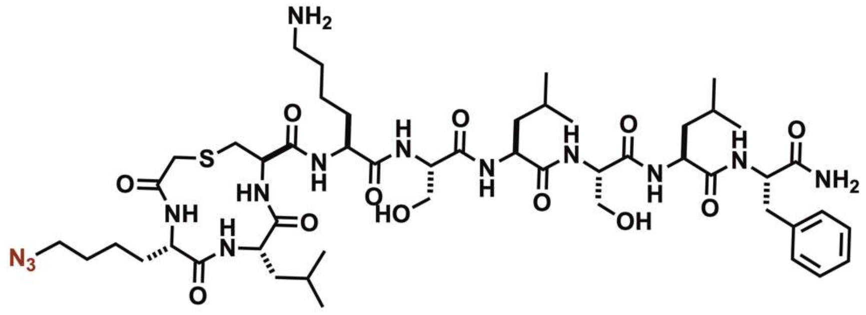

Analytical HPLC Chromatogram of Lar5

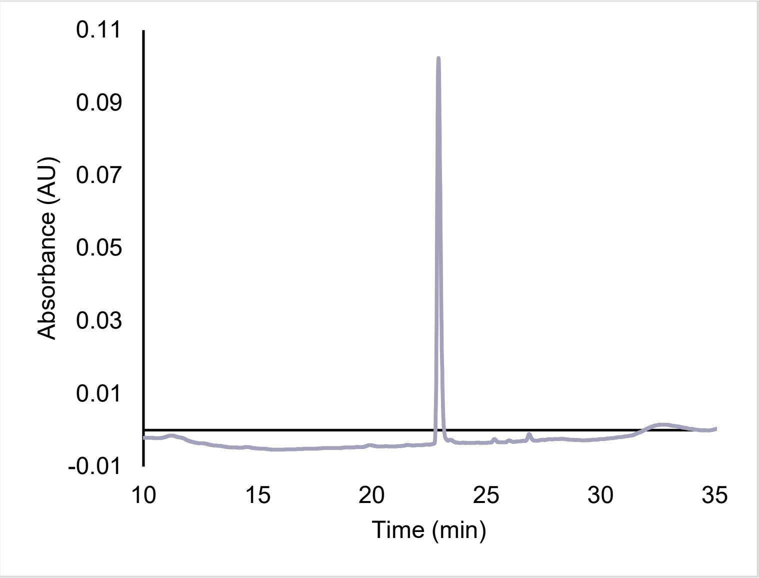

QTOF High Resolution Mass Spectrum for Lar5 (m/z 1103.5957 for [M+H]+)

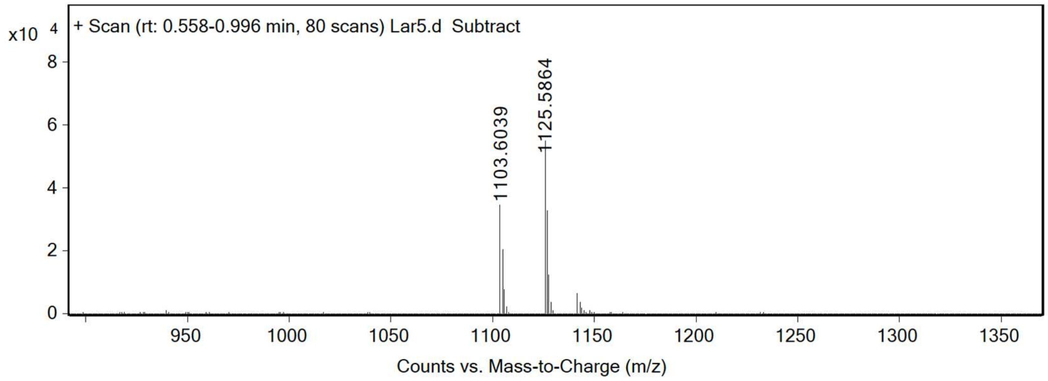

Dit0

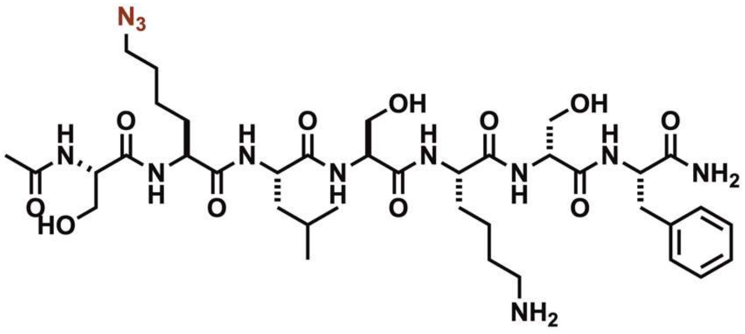

Analytical HPLC Chromatogram of Dit0

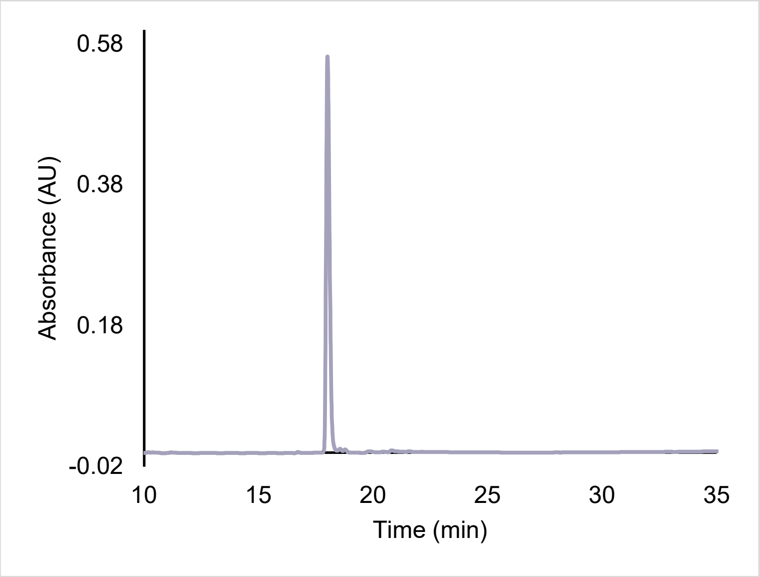

QTOF High Resolution Mass Spectrum for Dit0 (m/z 863.4661 for [M+H]+)

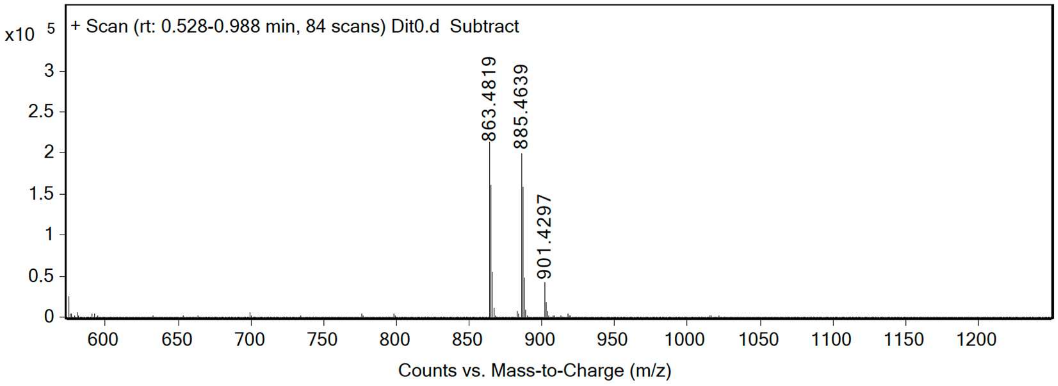

Dit1

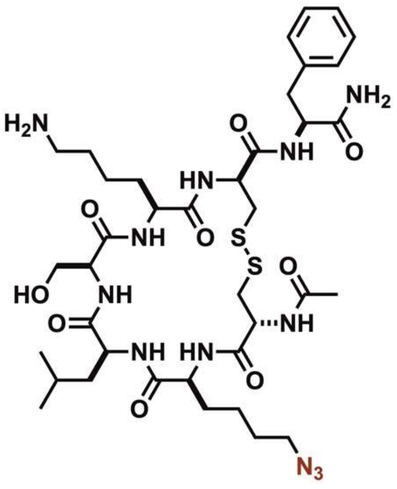

Analytical HPLC Chromatogram of Dit1

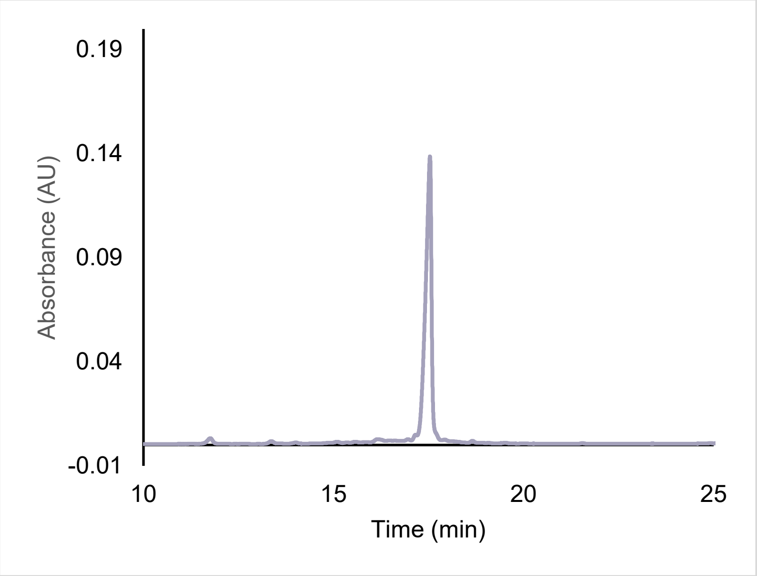

QTOF High Resolution Mass Spectrum for Dit1 (m/z 893.4048 for [M+H]+)

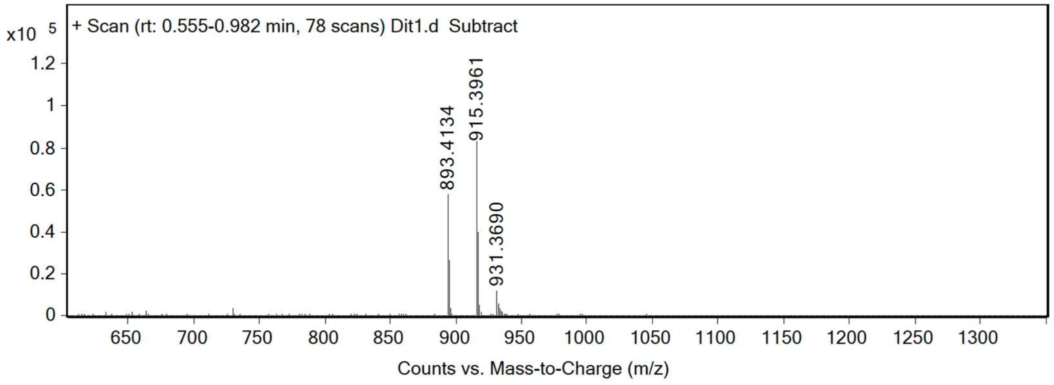

Dit2

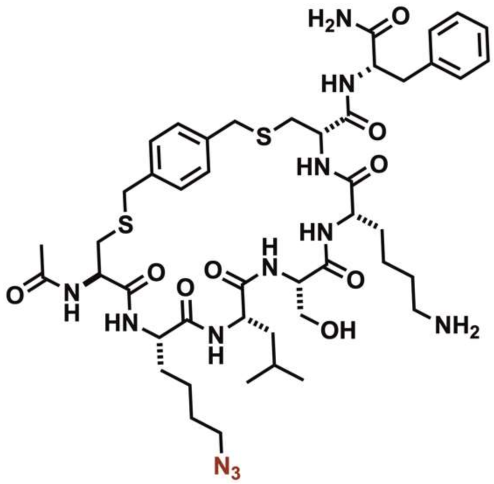

Analytical HPLC Chromatogram of Dit2

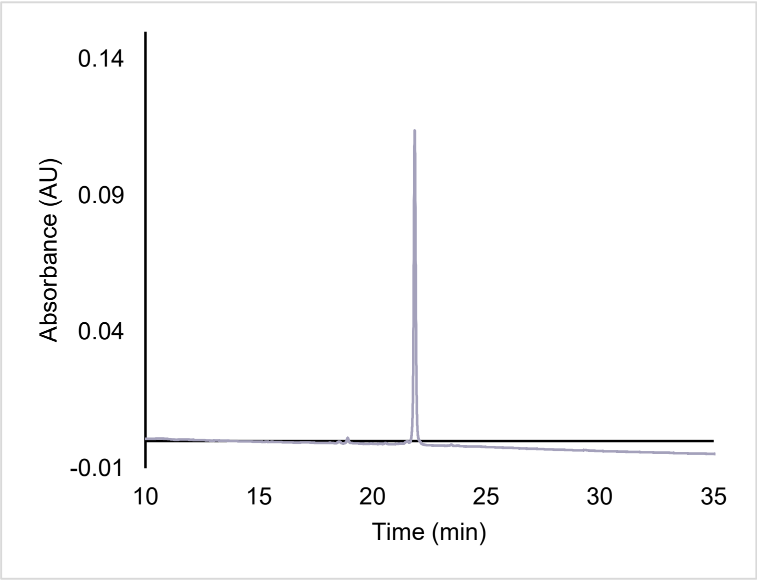

QTOF High Resolution Mass Spectrum for Dit1 (m/z 997.4674 for [M+H]+)

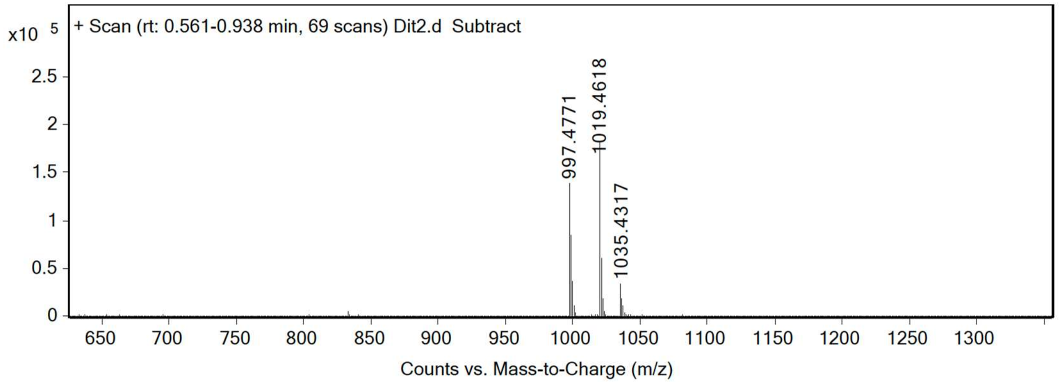

Nmet0

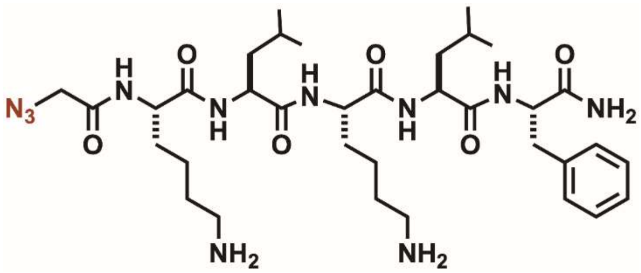

Analytical HPLC Chromatogram of **Nmet0**

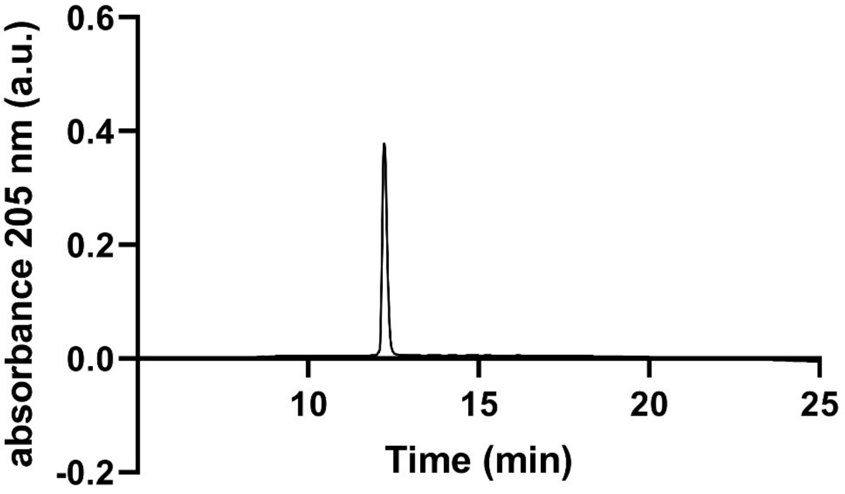

QTOF High Resolution Mass Spectrum for **Nmet0** (m/z 730.4723 for [M+H]+)

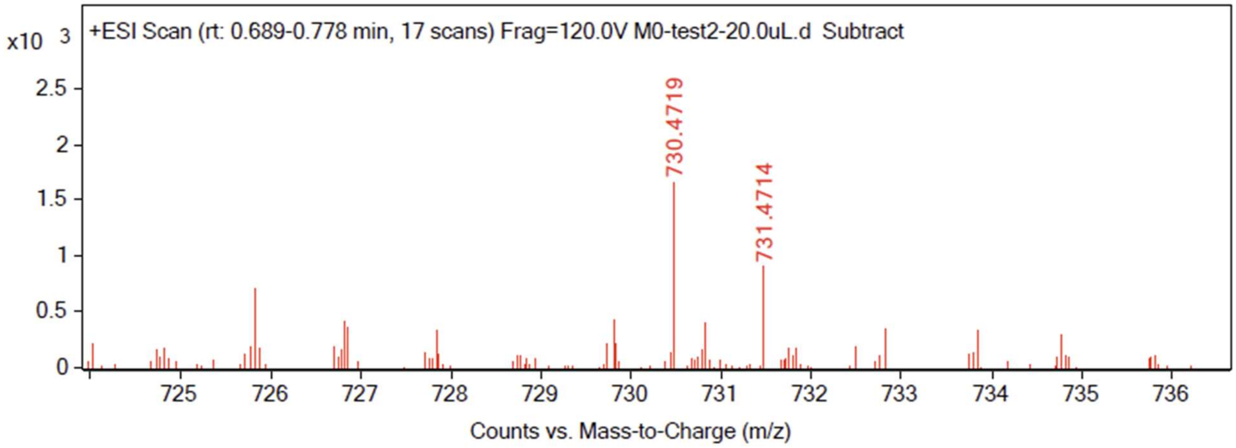

Nmet1

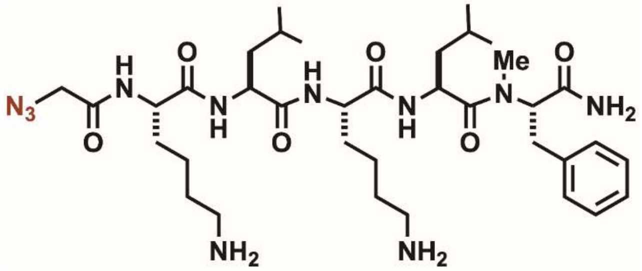

Analytical HPLC Chromatogram of **Nmet1**

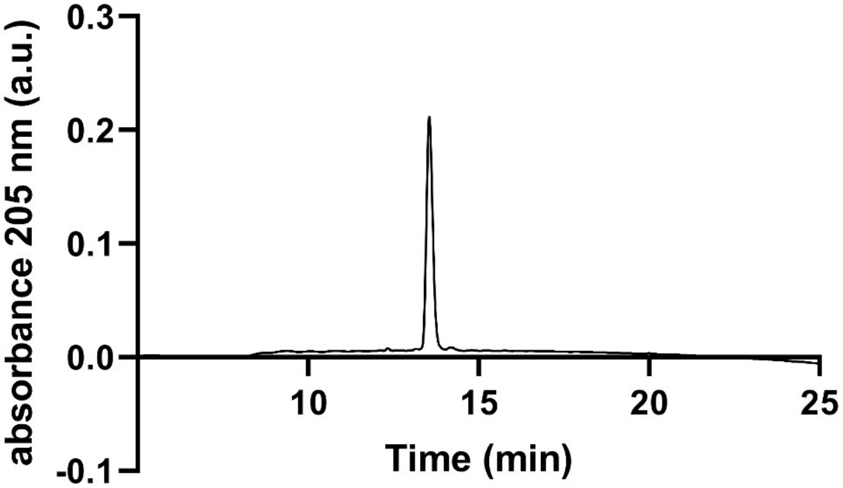

QTOF High Resolution Mass Spectrum for **Nmet1** (m/z 744.4879 for [M+H]+)

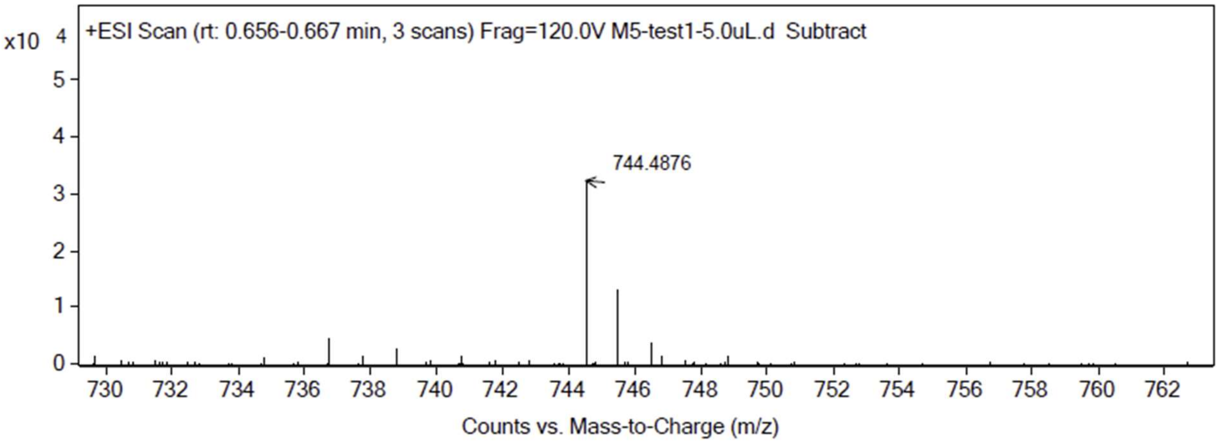

Nmet2

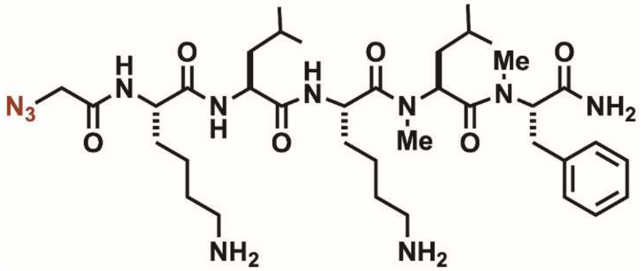

Analytical HPLC Chromatogram of **Nmet2**

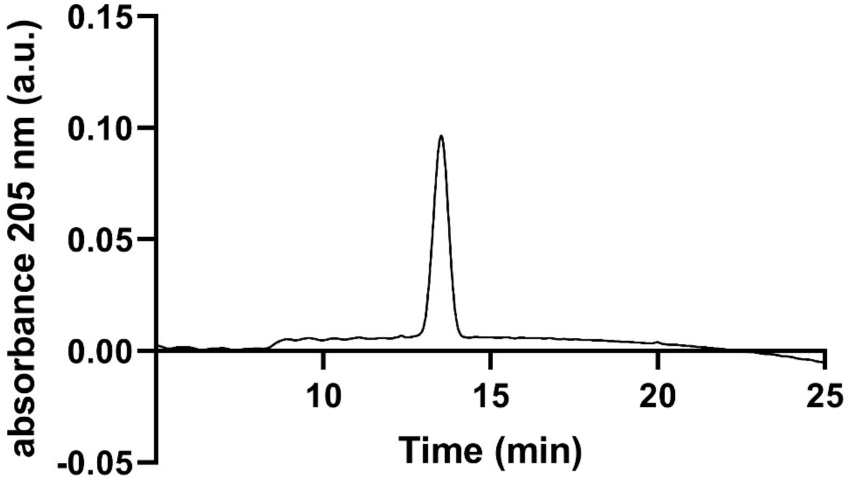

QTOF High Resolution Mass Spectrum for **Nmet2** (m/z 758.5036 for [M+H]+)

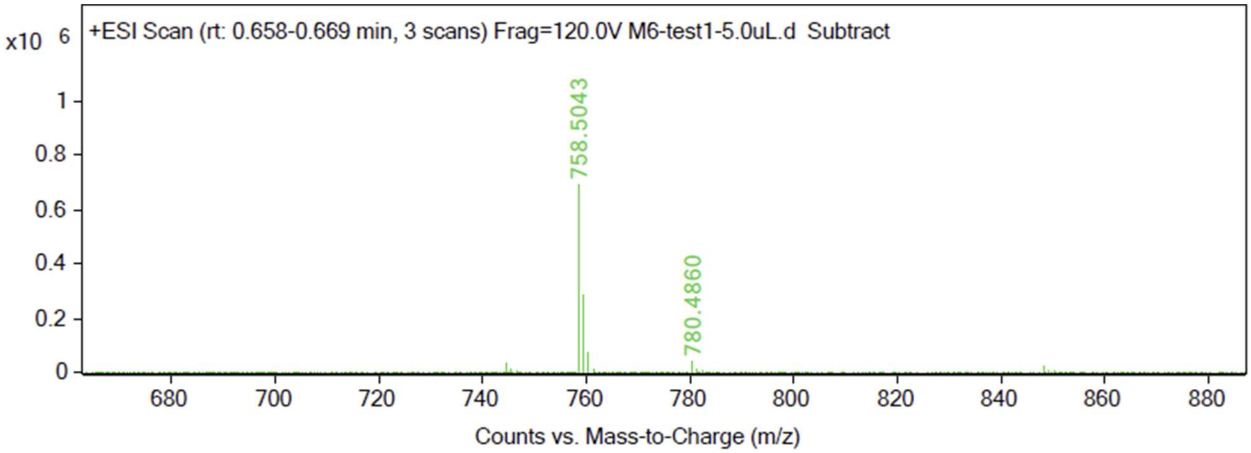

Nmet3

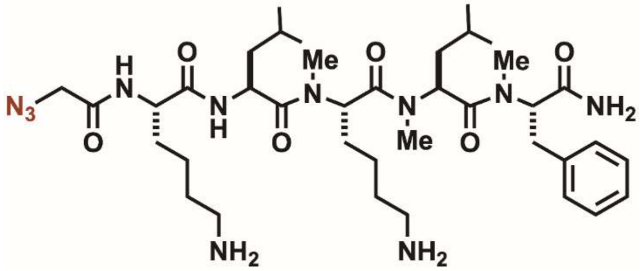

Analytical HPLC Chromatogram of **Nmet3**

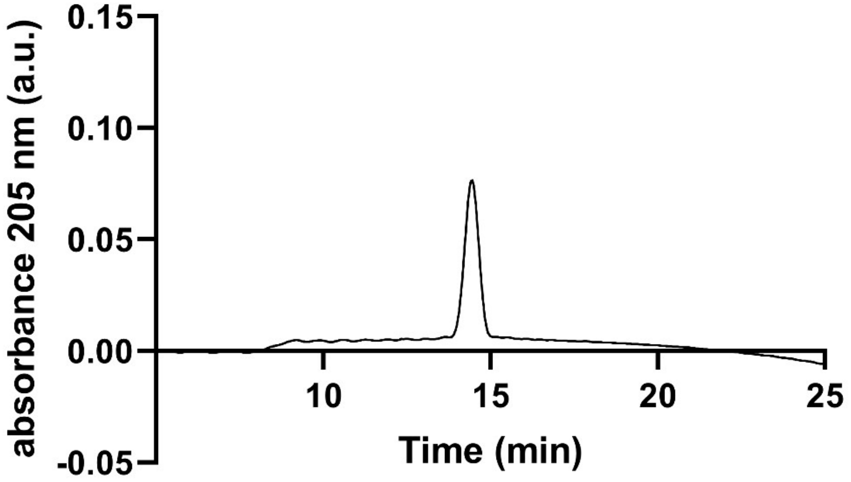

QTOF High Resolution Mass Spectrum for **Nmet3** (m/z 772.5192 for [M+H]+)

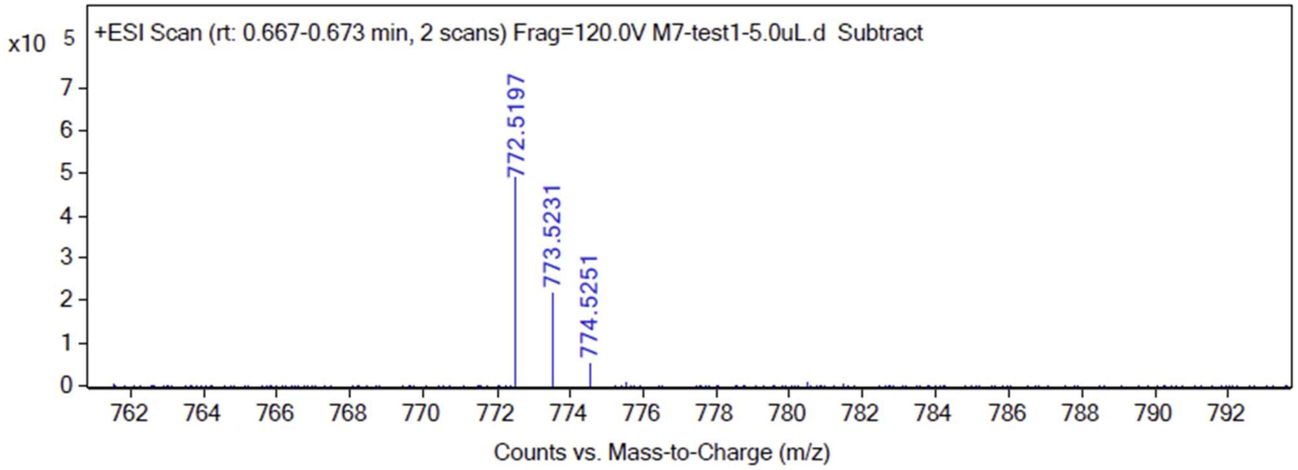

Nmet4

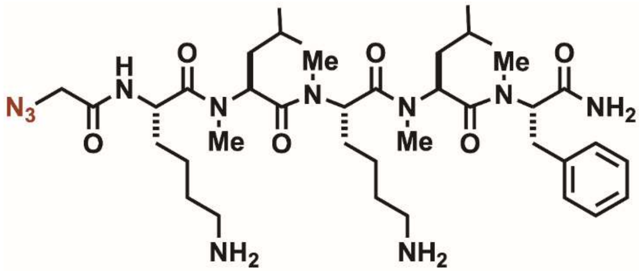

Analytical HPLC Chromatogram of **Nmet4**

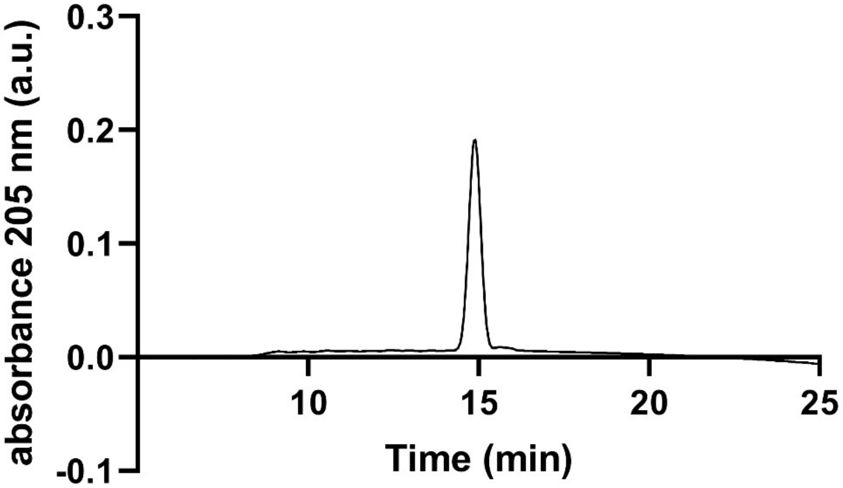

QTOF High Resolution Mass Spectrum for **Nmet4** (m/z 786.5349 for [M+H]+)

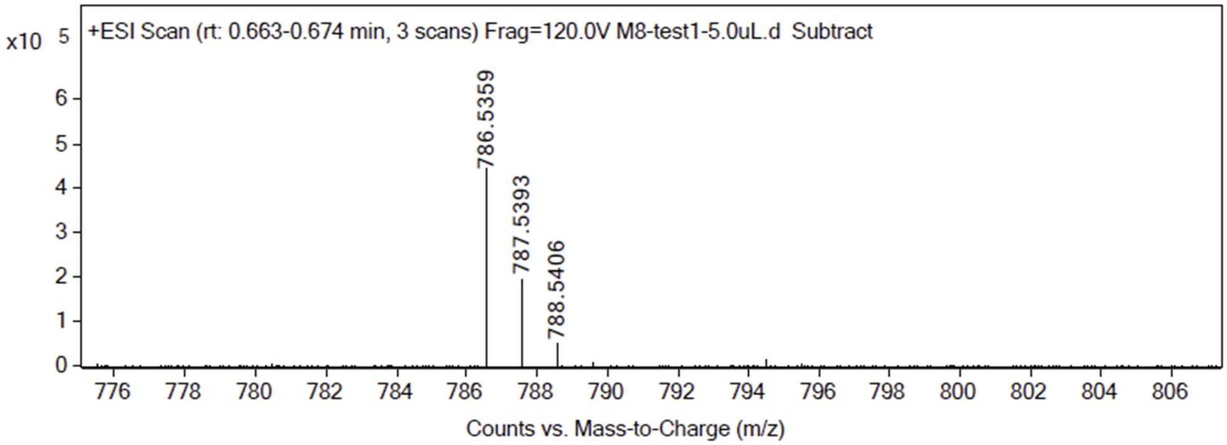

Nmet5

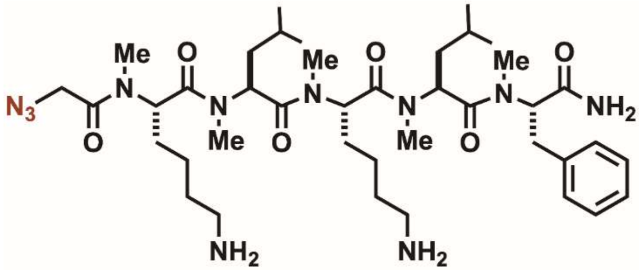

Analytical HPLC Chromatogram of **Nmet5**

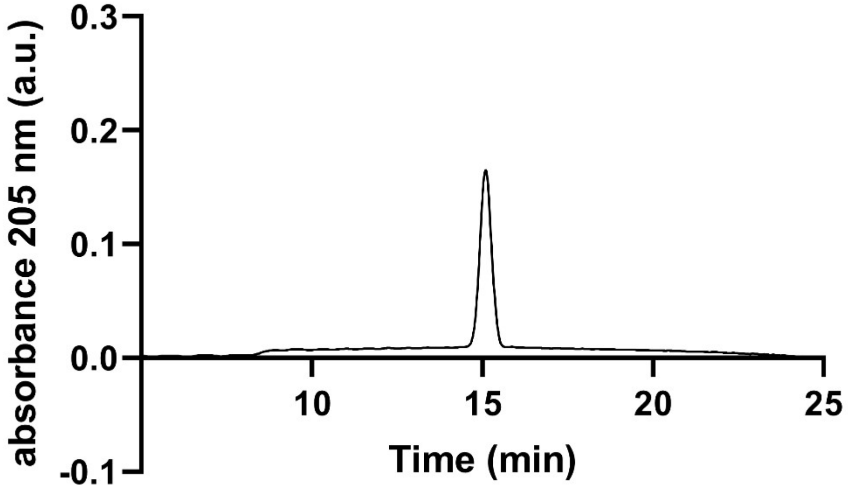

QTOF High Resolution Mass Spectrum for **Nmet5** (m/z 800.5505 for [M+H]+)

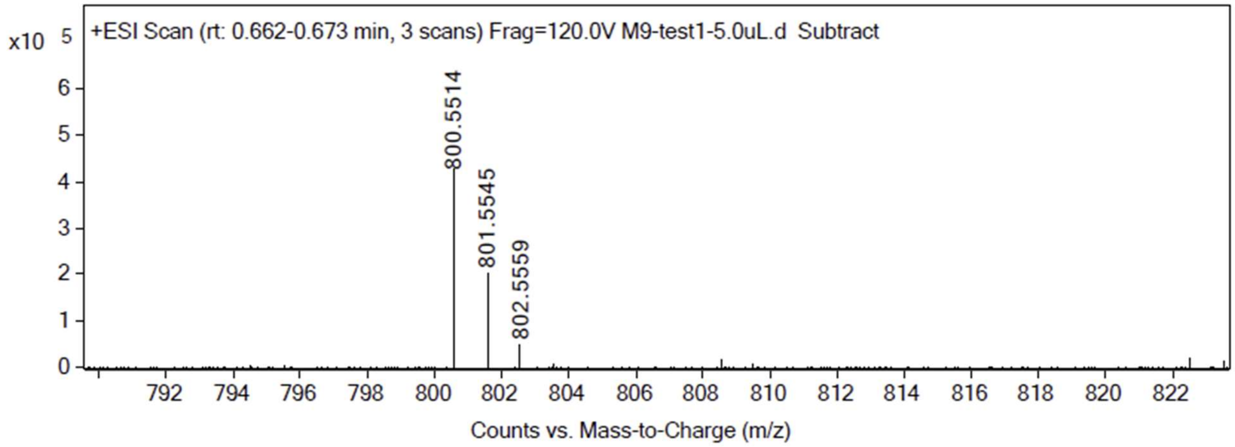

Nmet6

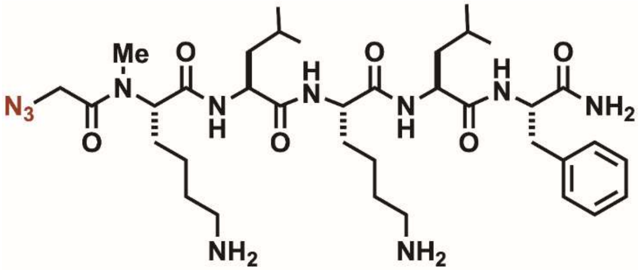

Analytical HPLC Chromatogram of **Nmet6**

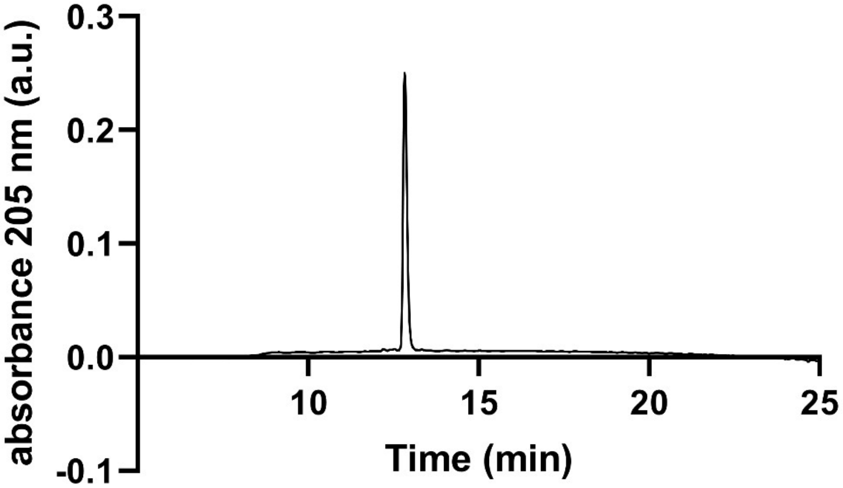

QTOF High Resolution Mass Spectrum for **Nmet6** (m/z 744.4879 for [M+H]+)

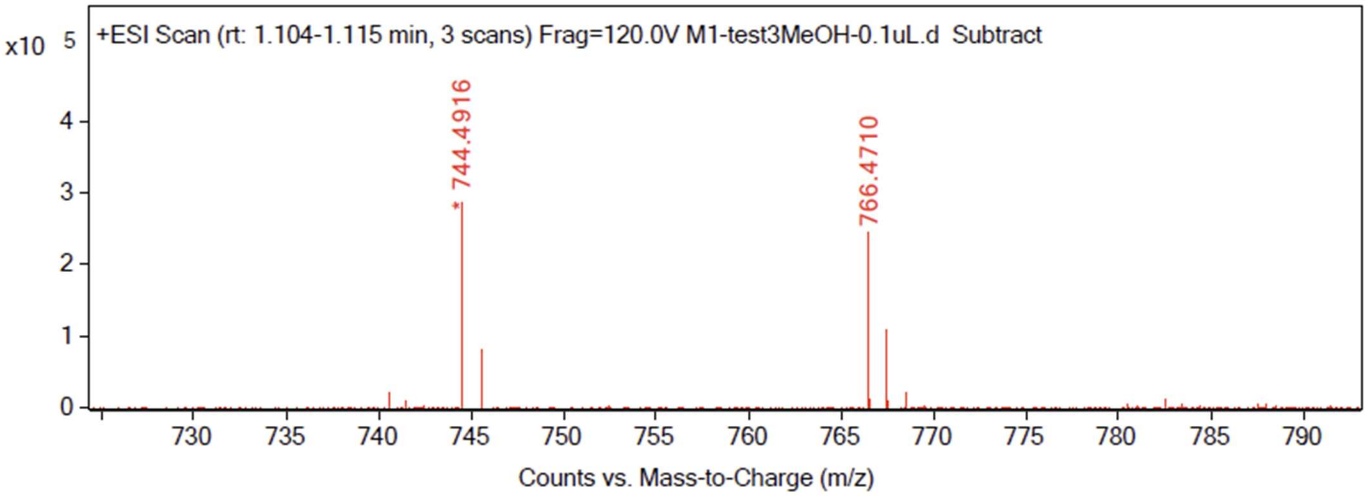

Nmet7

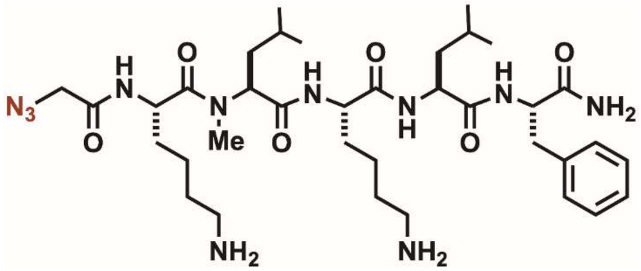

Analytical HPLC Chromatogram of **Nmet7**

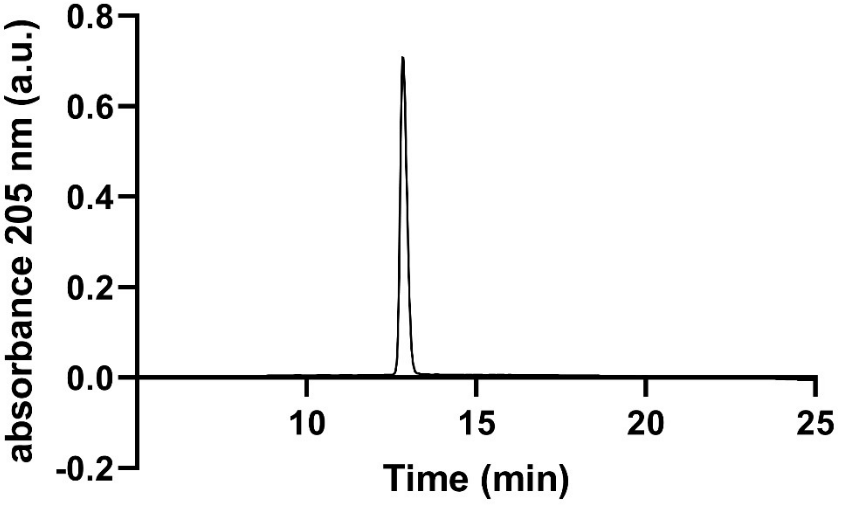

QTOF High Resolution Mass Spectrum for **Nmet7** (m/z 744.4879 for [M+H]+)

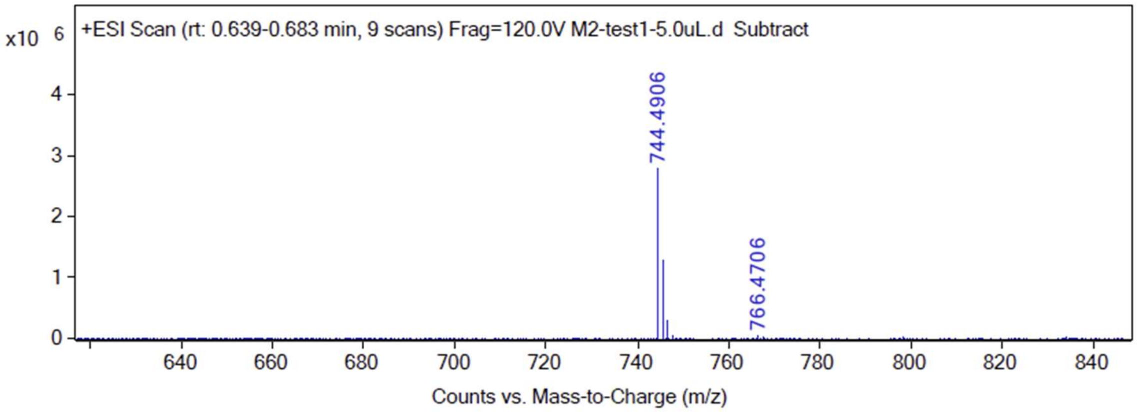

Nmet8

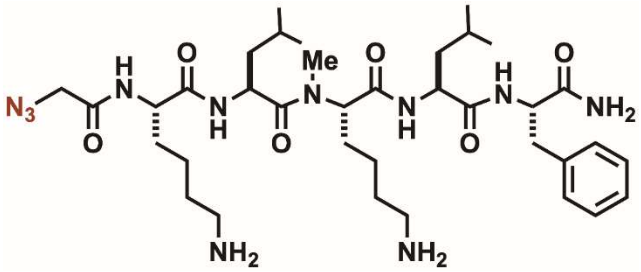

Analytical HPLC Chromatogram of **Nmet8**

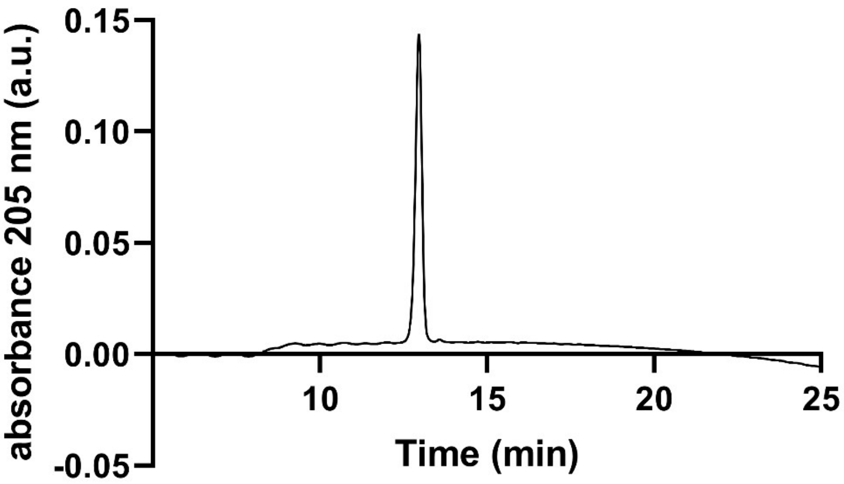

QTOF High Resolution Mass Spectrum for **Nmet8** (m/z 744.4879 for [M+H]+)

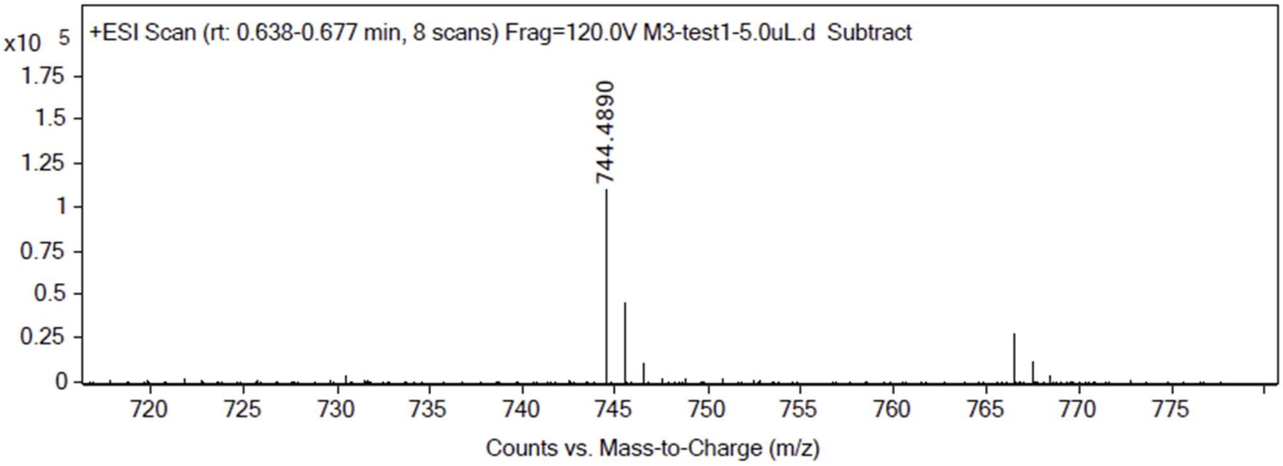

Nmet9

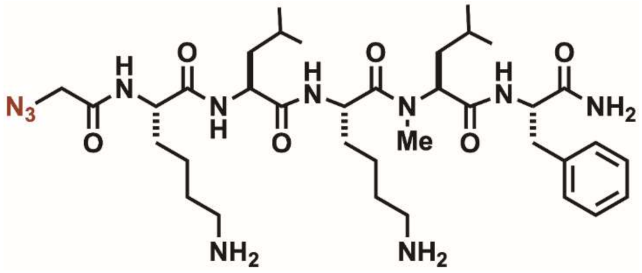

Analytical HPLC Chromatogram of **Nmet9**

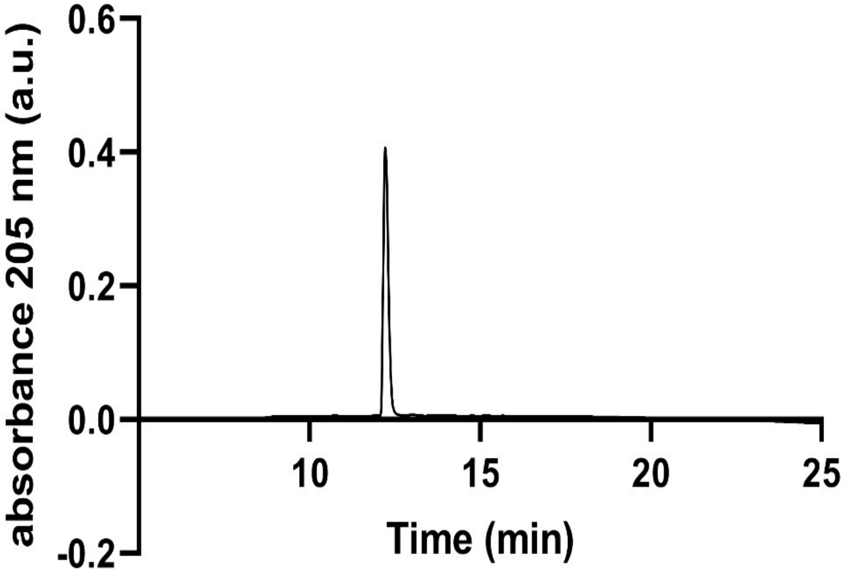

QTOF High Resolution Mass Spectrum for **Nmet9** (m/z 744.4879 for [M+H]+)

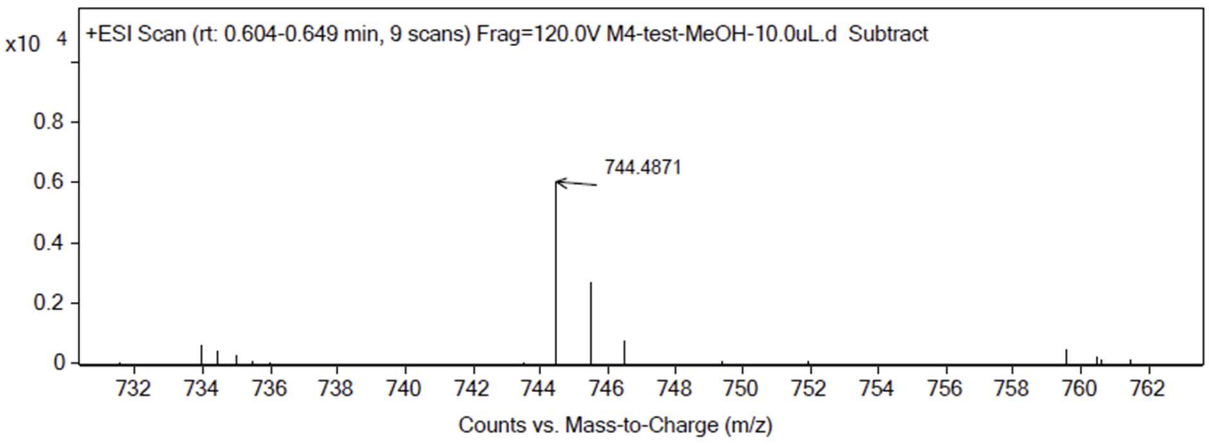

Nalk1

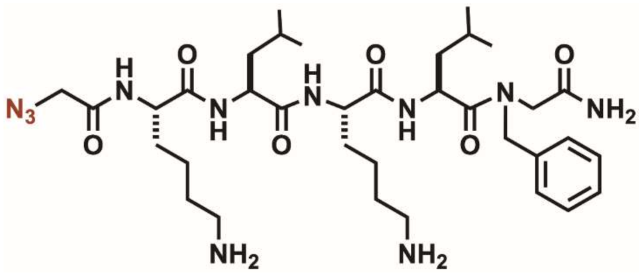

Analytical HPLC Chromatogram of **Nalk1**

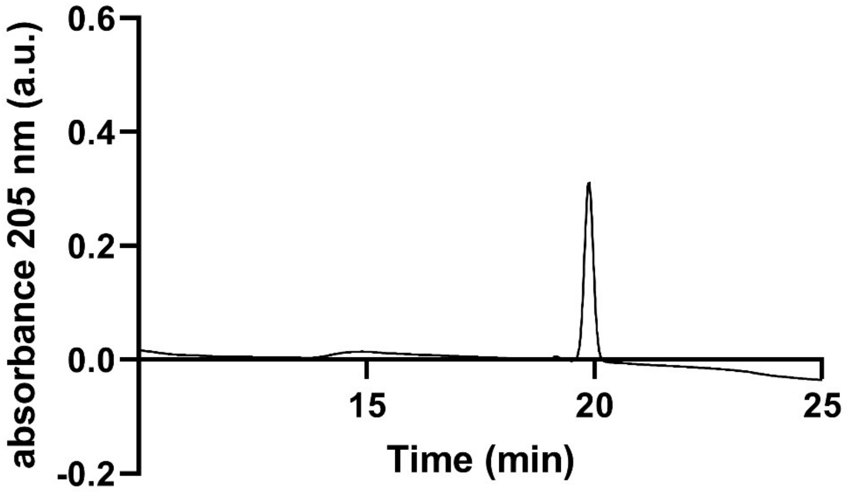

QTOF High Resolution Mass Spectrum for **Nalk1** (m/z 730.4723 for [M+H]+)

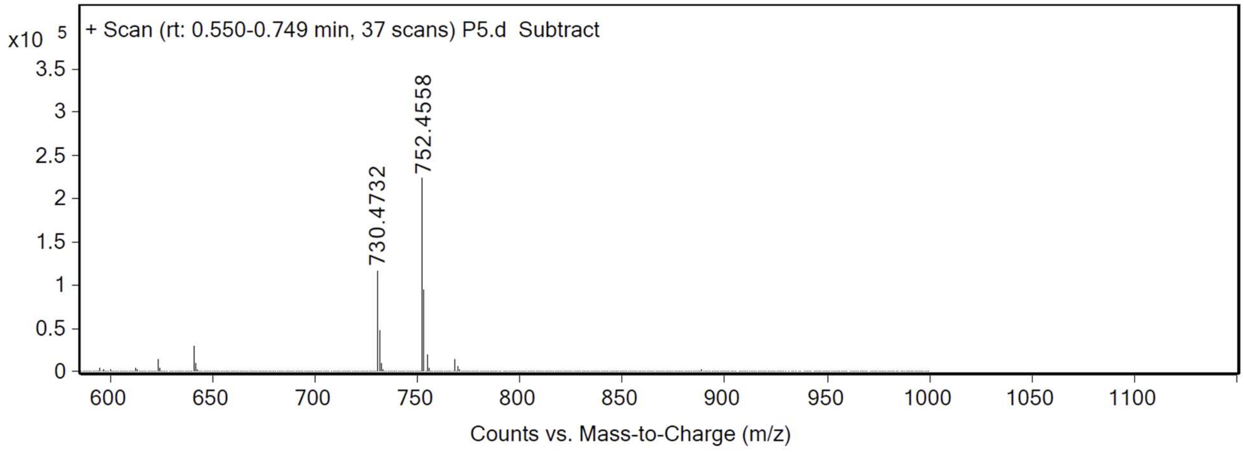

Nalk2

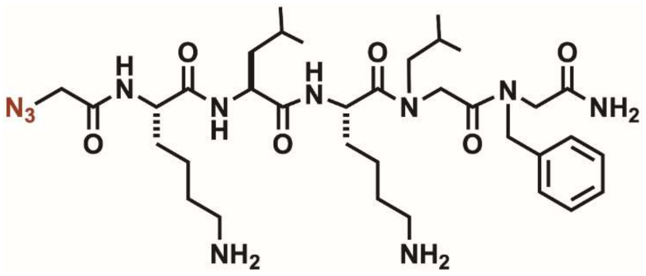

Analytical HPLC Chromatogram of **Nalk2**

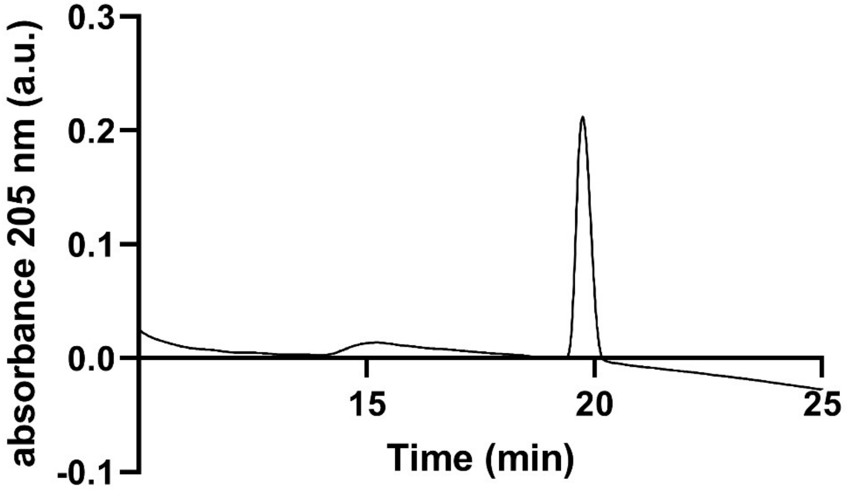

QTOF High Resolution Mass Spectrum for **Nalk2** (m/z 730.4723 for [M+H]+)

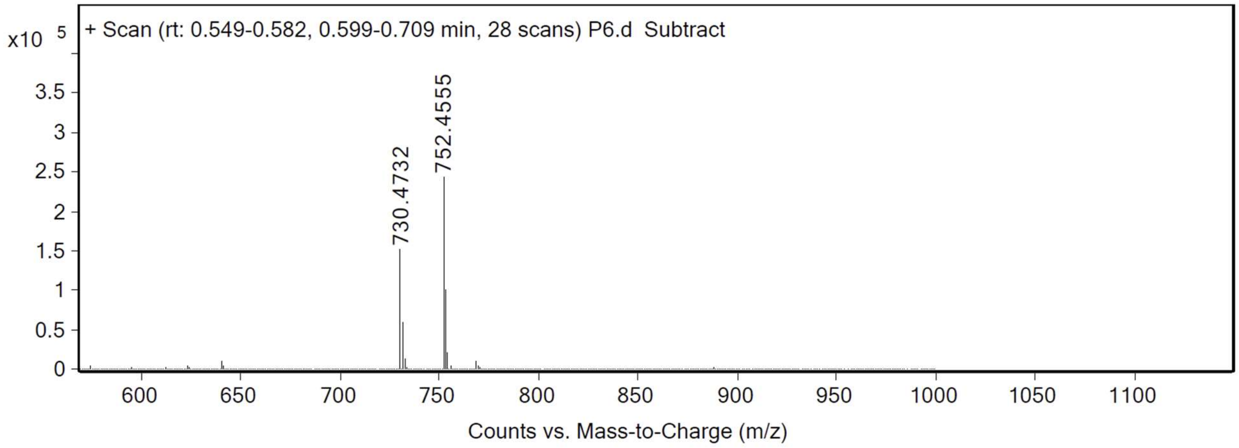

Nalk3

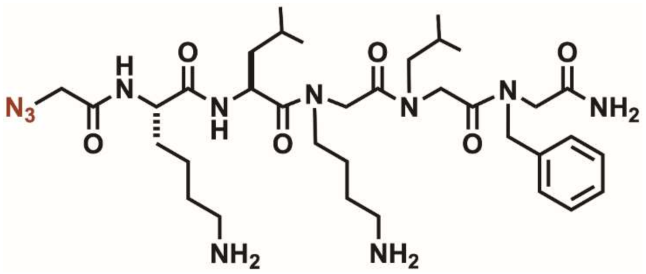

Analytical HPLC Chromatogram of **Nalk3**

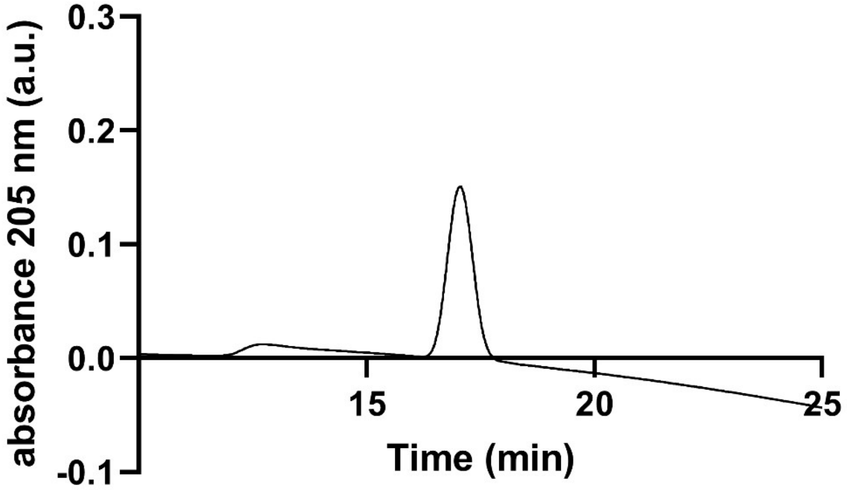

QTOF High Resolution Mass Spectrum for **Nalk3** (m/z 730.4723 for [M+H]+)

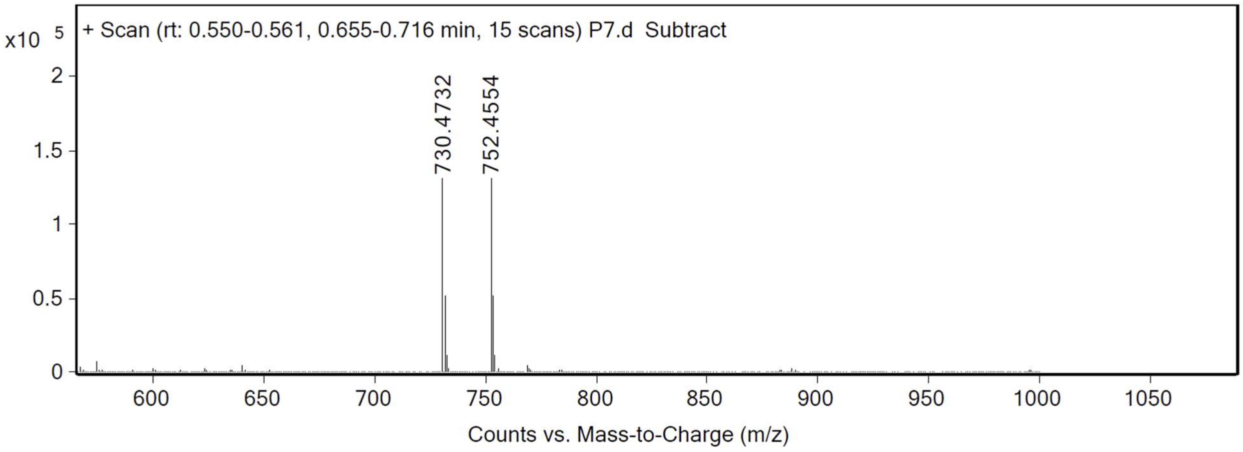

Nalk4

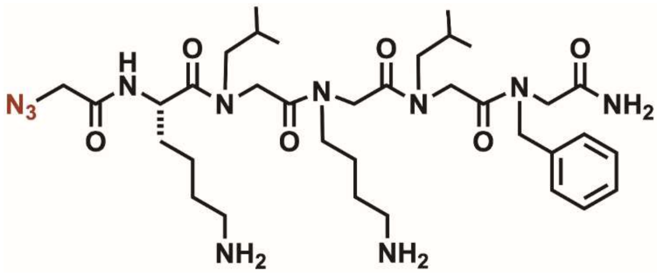

Analytical HPLC Chromatogram of **Nalk4**

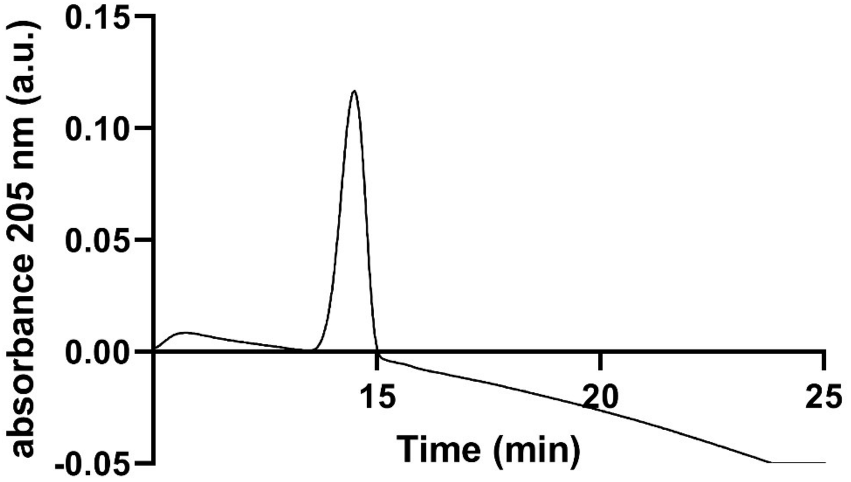

QTOF High Resolution Mass Spectrum for **Nalk4** (m/z 730.4723 for [M+H]+)

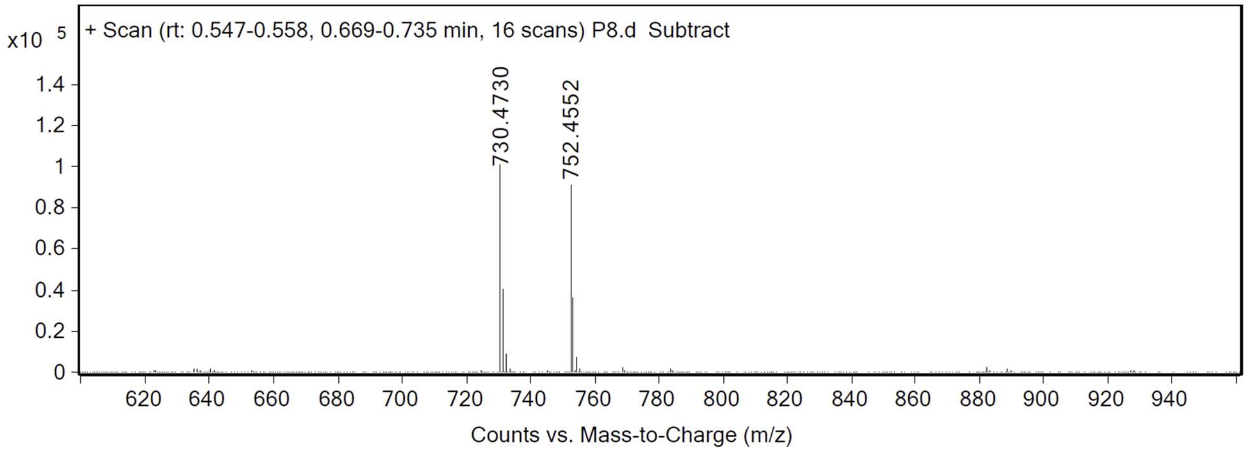

Nalk5

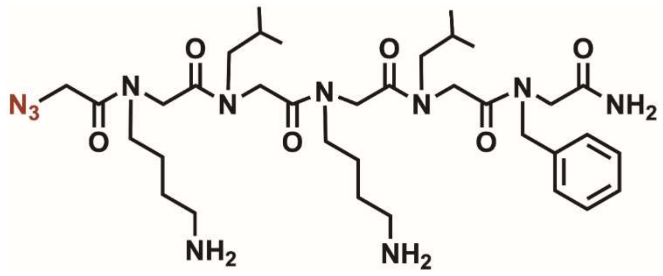

Analytical HPLC Chromatogram of **Nalk5**

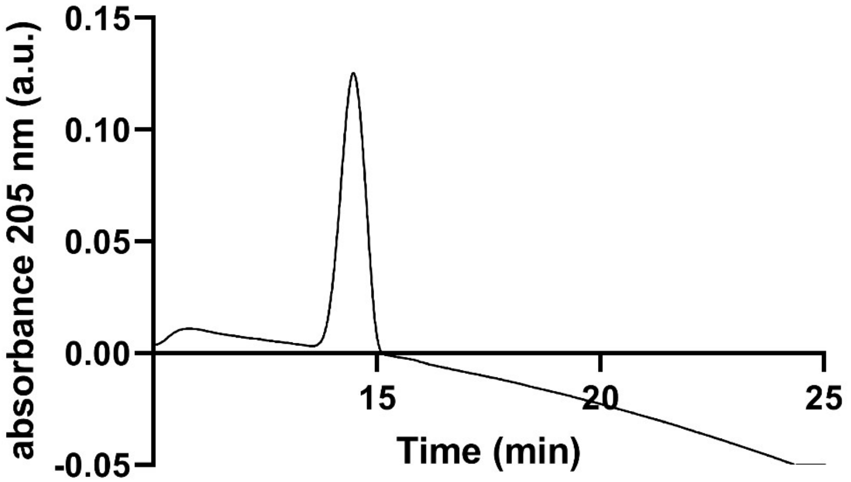

QTOF High Resolution Mass Spectrum for **Nalk5** (m/z 730.4723 for [M+H]+)

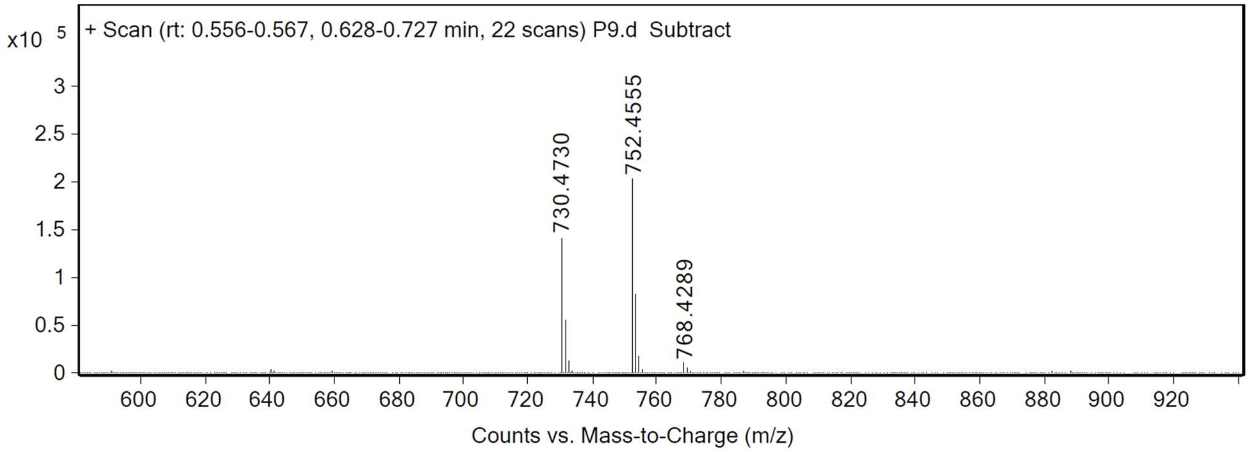

Nalk6

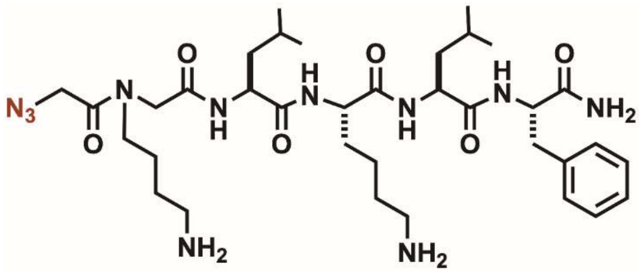

Analytical HPLC Chromatogram of **Nalk6**

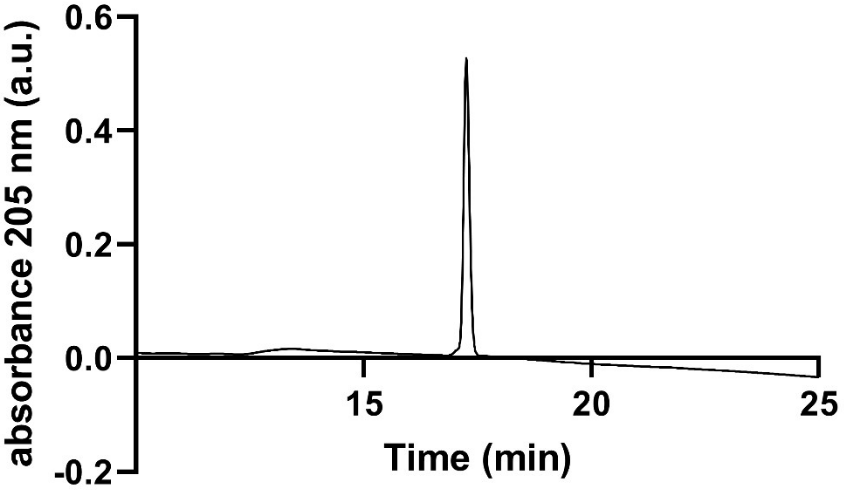

QTOF High Resolution Mass Spectrum for **Nalk6** (m/z 730.4723 for [M+H]+)

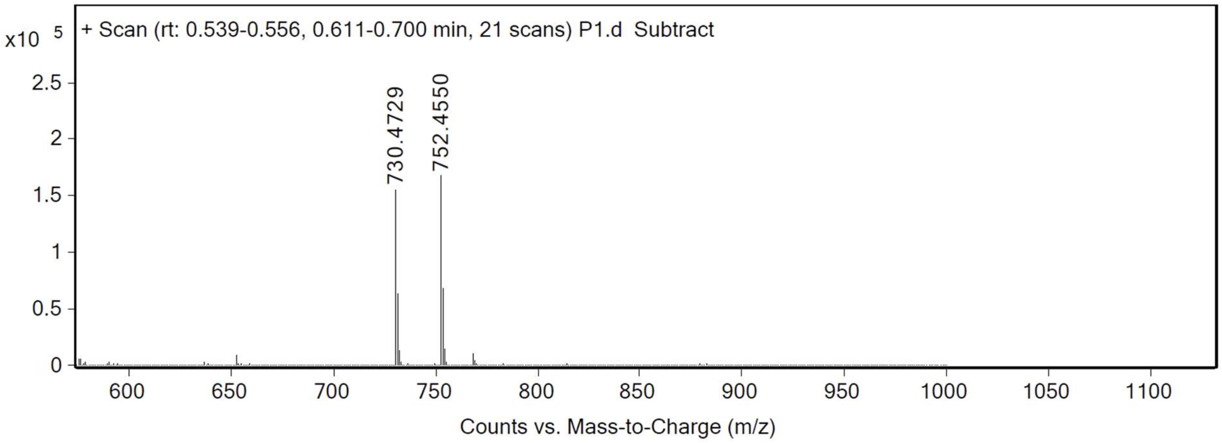

Nalk7

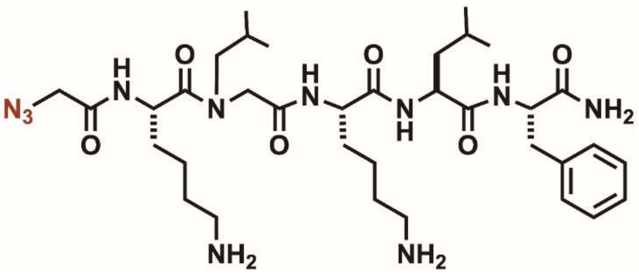

Analytical HPLC Chromatogram of **Nalk7**

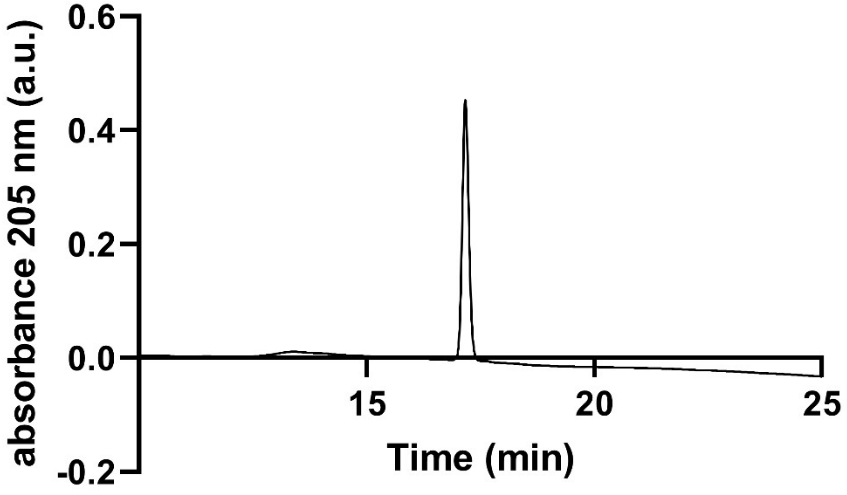

QTOF High Resolution Mass Spectrum for **Nalk7** (m/z 730.4723 for [M+H]+)

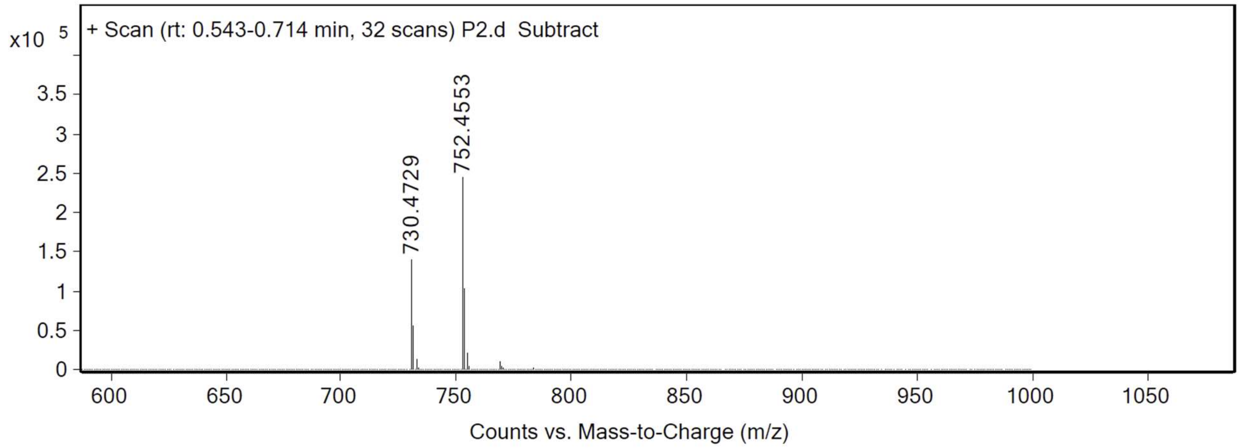

Nalk8

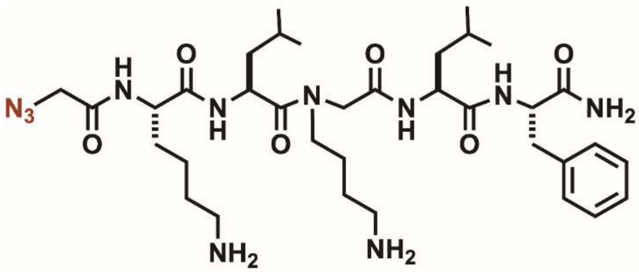

Analytical HPLC Chromatogram of **Nalk8**

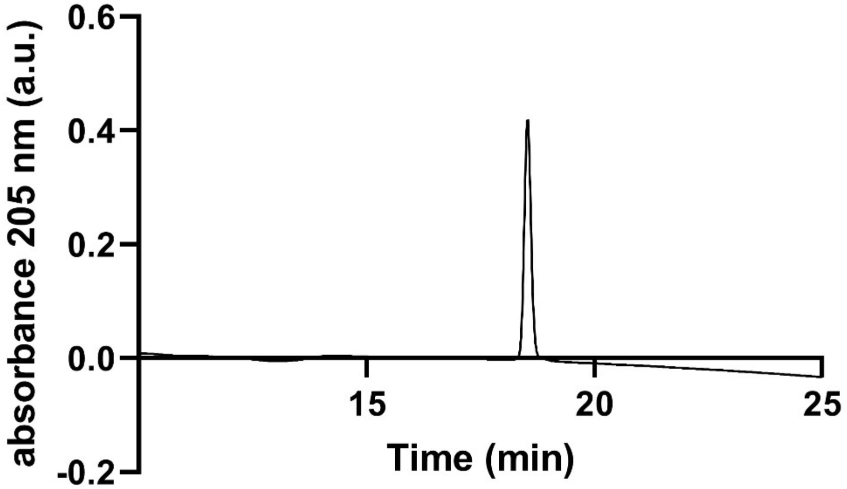

QTOF High Resolution Mass Spectrum for **Nalk8** (m/z 730.4723 for [M+H]+)

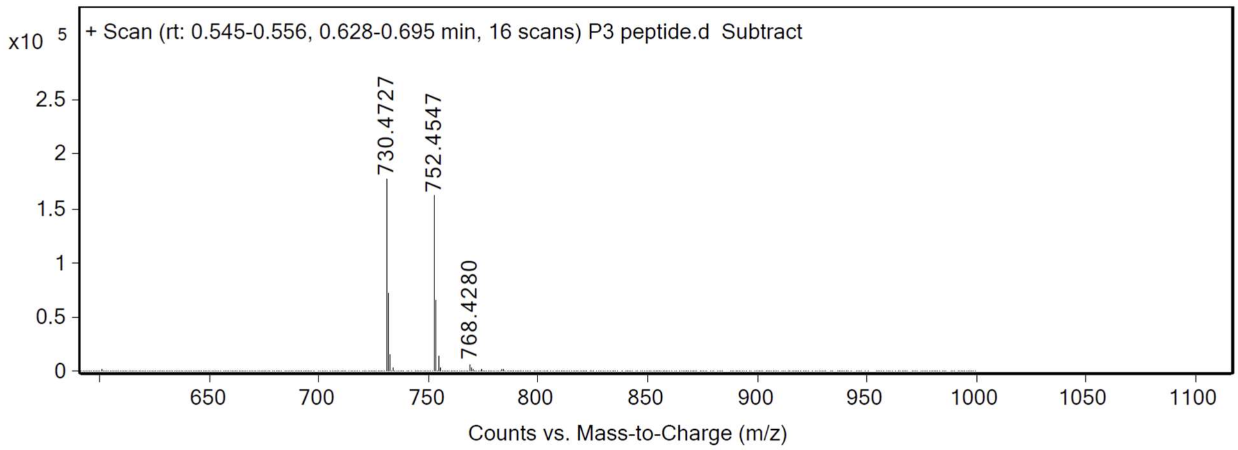

Nalk9

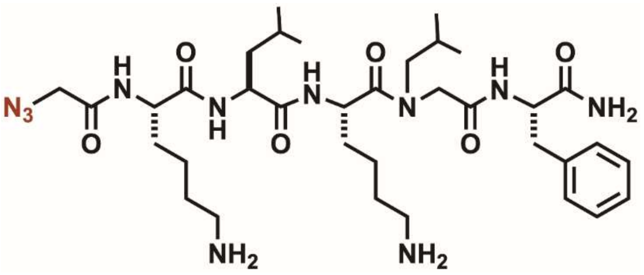

Analytical HPLC Chromatogram of **Nalk9**

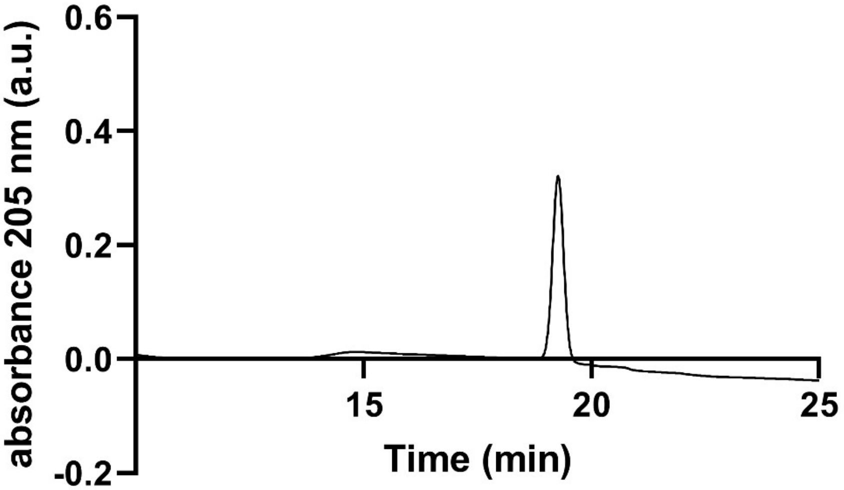

QTOF High Resolution Mass Spectrum for **Nalk9** (m/z 730.4723 for [M+H]+)

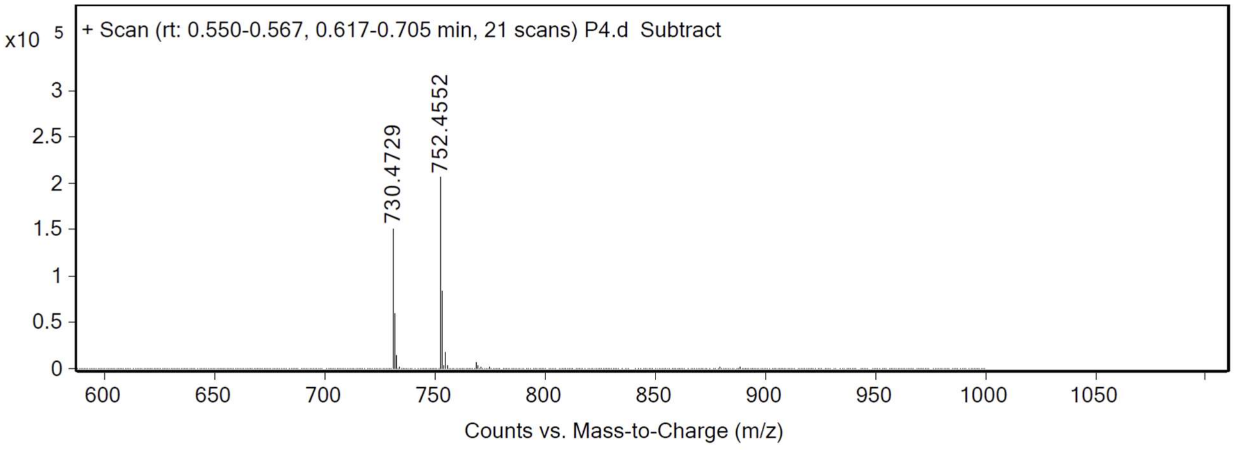

Oct-triA1

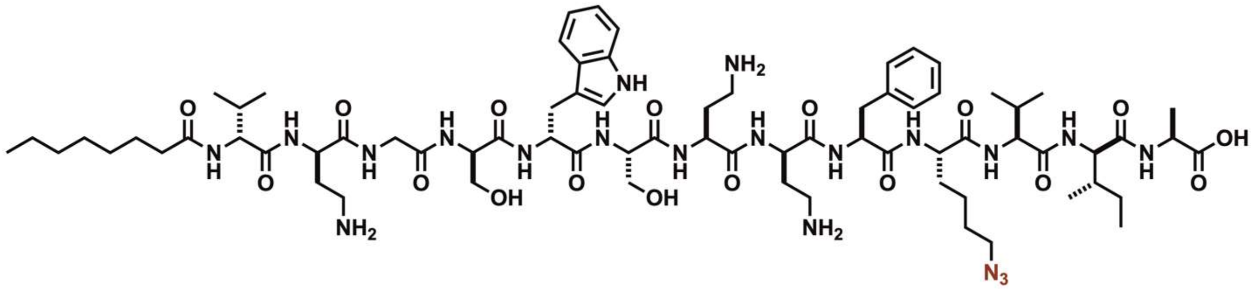

Analytical HPLC Chromatogram of **Oct-triA1**

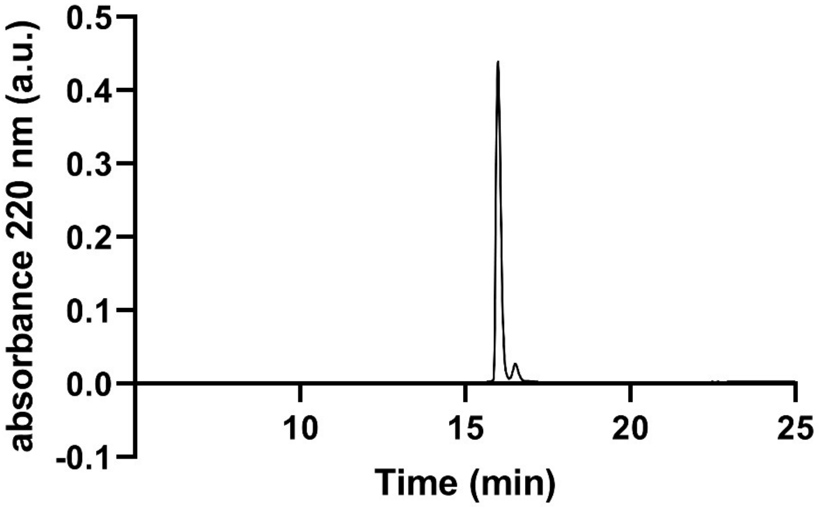

QTOF High Resolution Mass Spectrum for **Oct-triA1** (m/z 1545.8900 for [M+H]+)

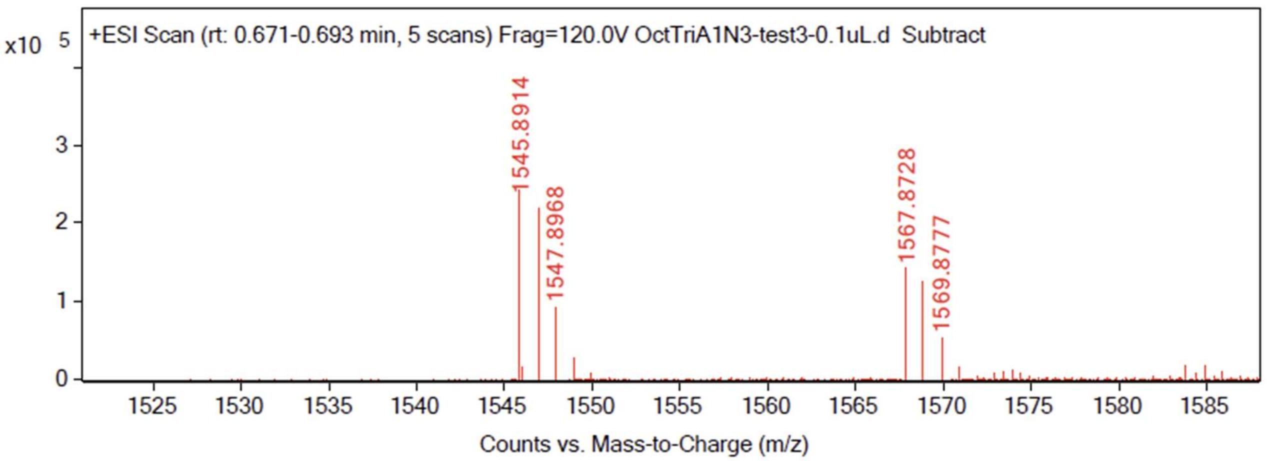

Nmet1-Oct-triA1

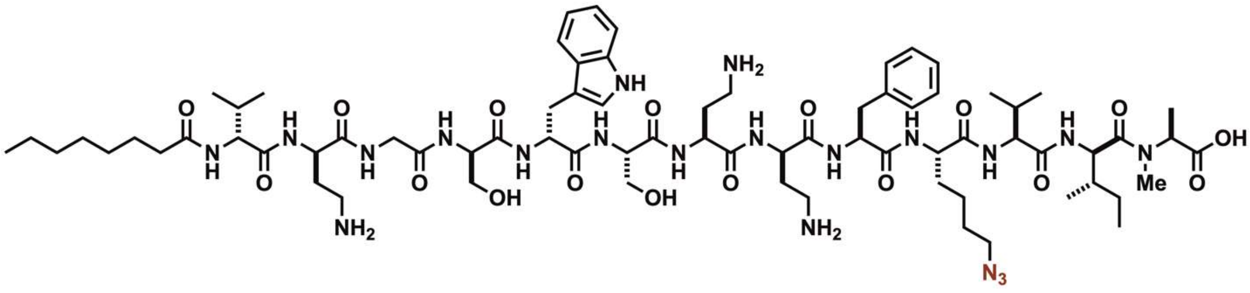

Analytical HPLC Chromatogram of **Nmet1-Oct-triA1**

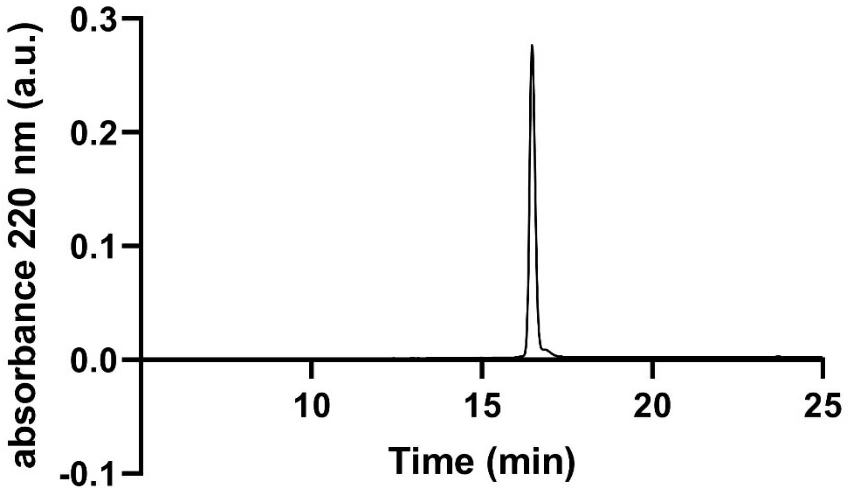

QTOF High Resolution Mass Spectrum for **Nmet1-Oct-triA1** (m/z 1559.9057 for [M+H]+)

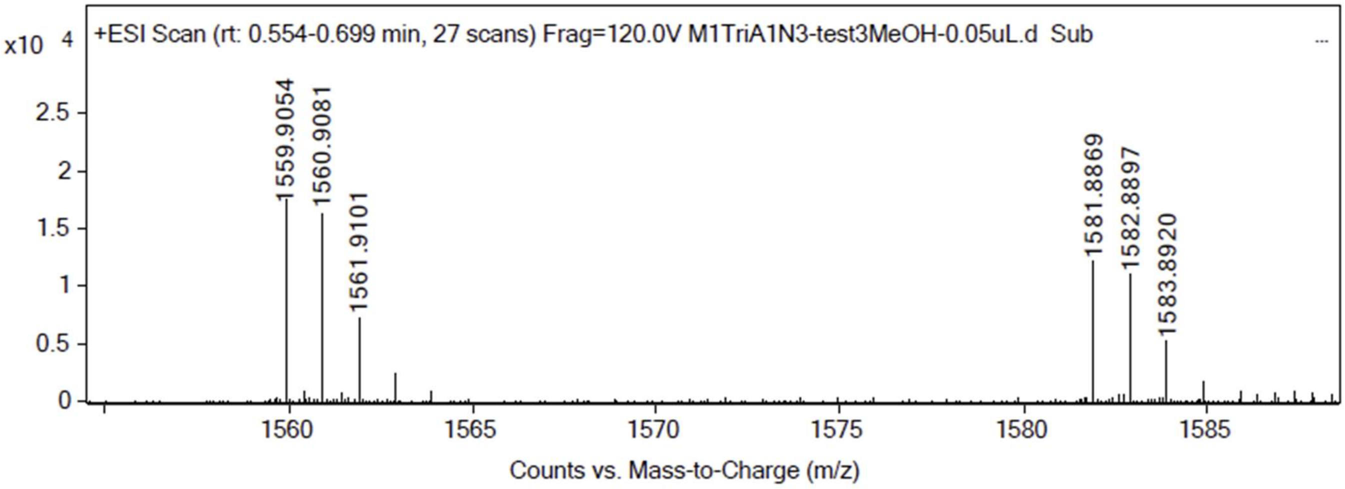

Nmet2-Oct-triA1

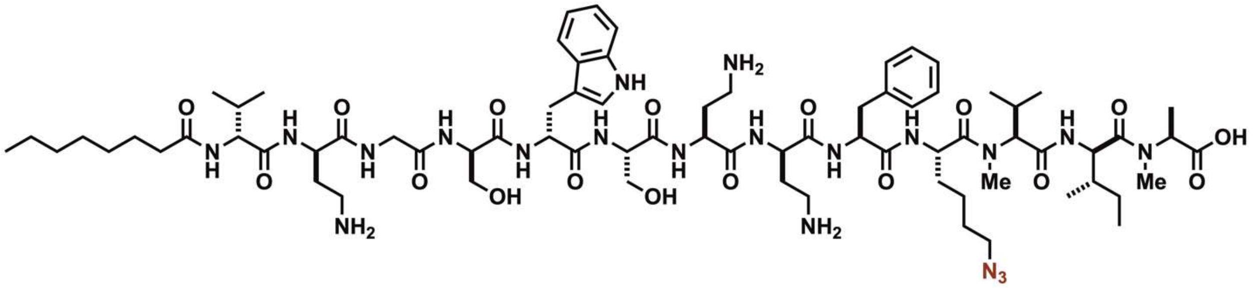

Analytical HPLC Chromatogram of **Nmet2-Oct-triA1**

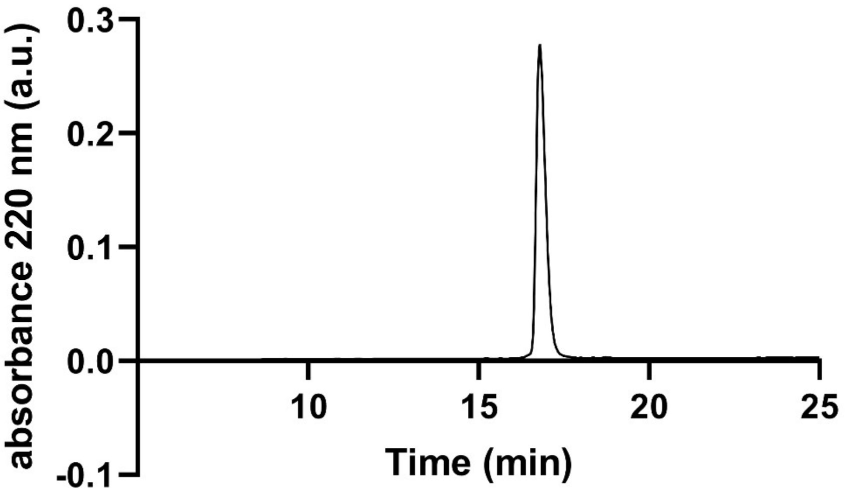

QTOF High Resolution Mass Spectrum for **Nmet2-Oct-triA1** (m/z 1573.9213 for [M+H]+)

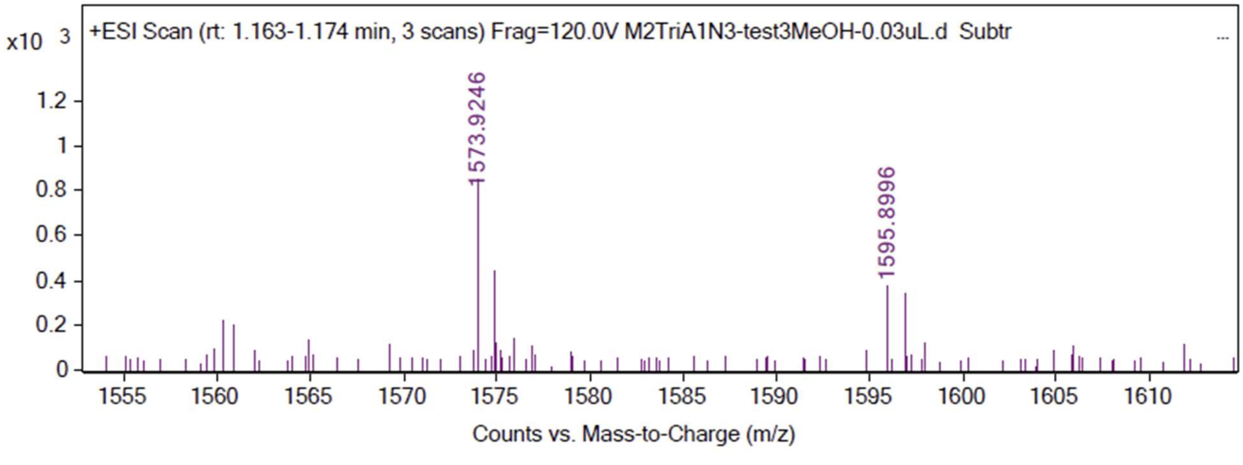

Nmet3-Oct-triA1

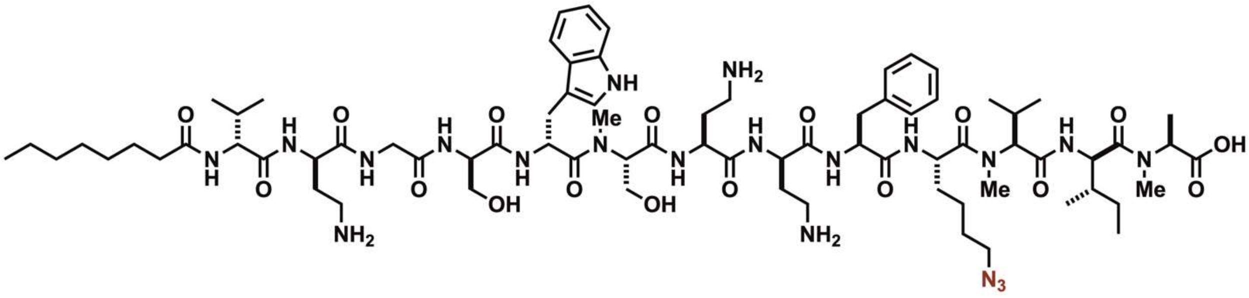

Analytical HPLC Chromatogram of **Nmet3-Oct-triA1**

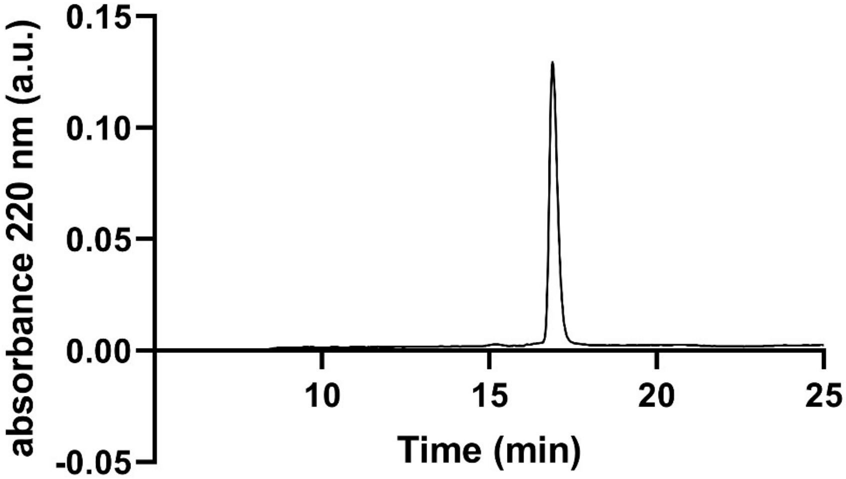

QTOF High Resolution Mass Spectrum for **Nmet3-Oct-triA1** (m/z 1587.9370 for [M+H]+)

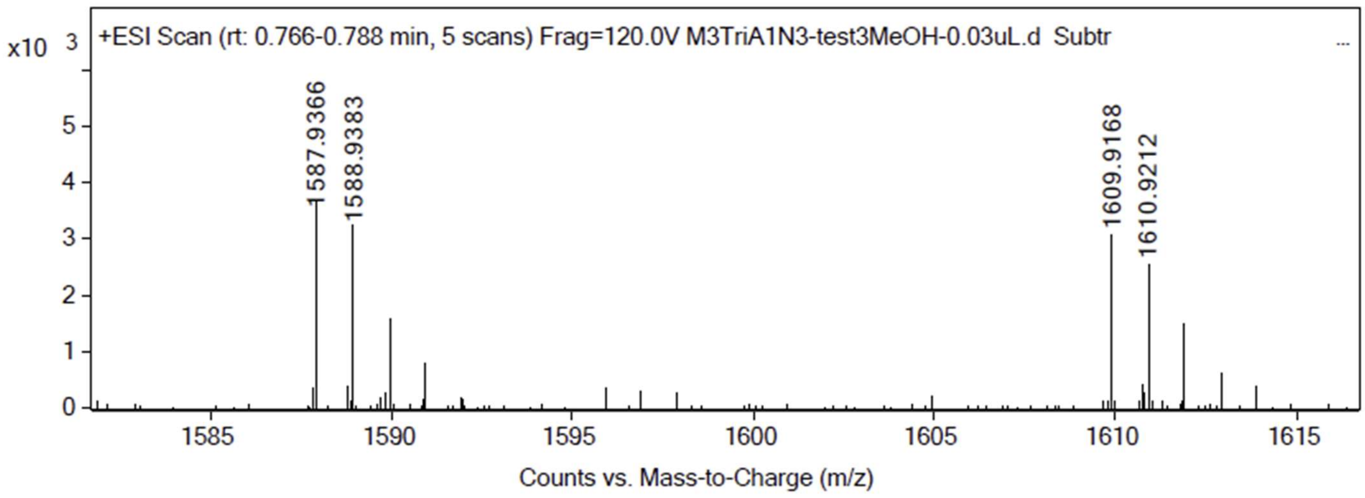

Cyc-Oct-triA1

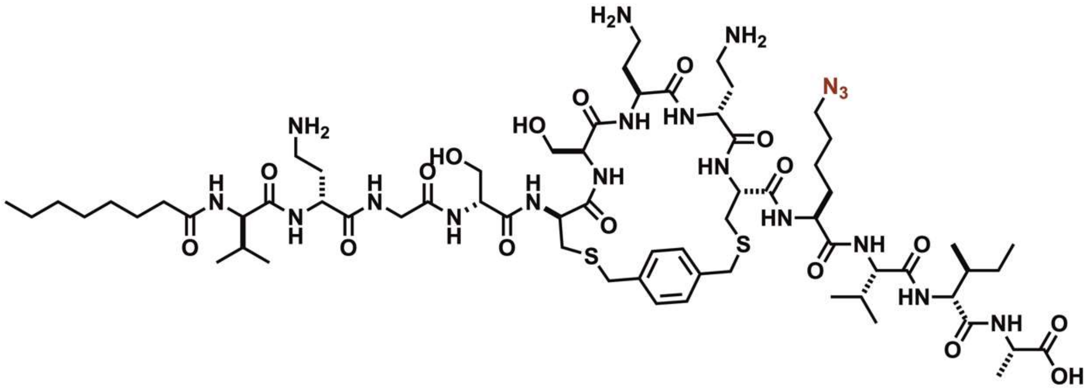

Analytical HPLC Chromatogram of **Cyc-Oct-triA1**

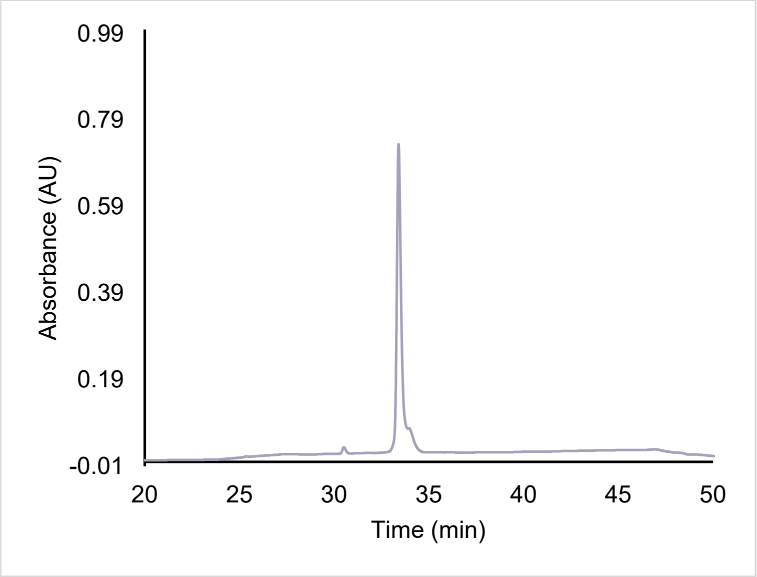

QTOF High Resolution Mass Spectrum for **Cyc-Oct-triA1** (m/z 1520.8003 for [M+H]+)

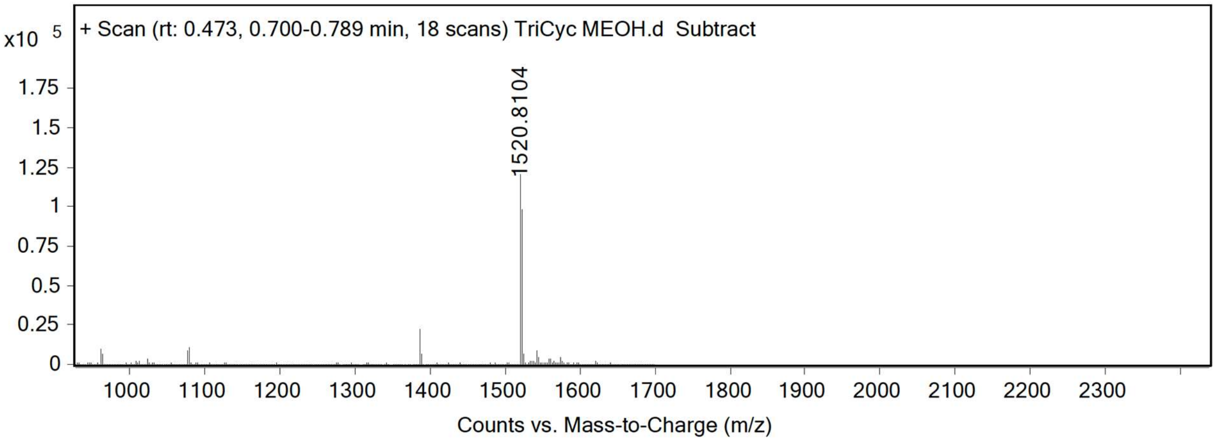

Lin-Oct-triA1

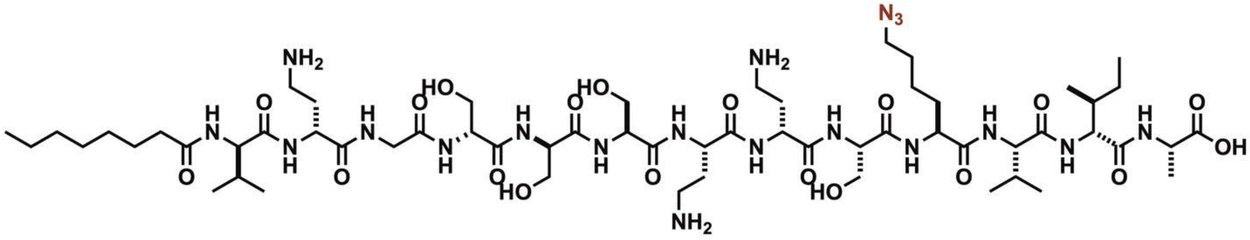

Analytical HPLC Chromatogram of **Lin-Oct-triA1**

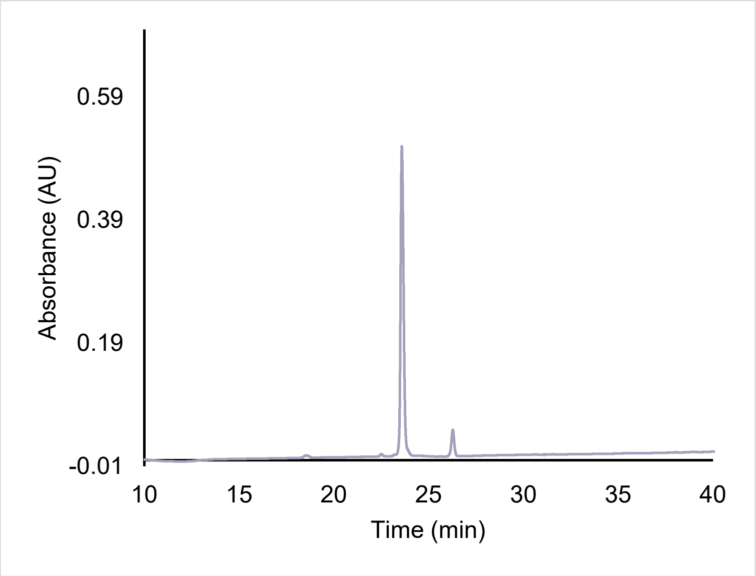

QTOF High Resolution Mass Spectrum for **Lin-Oct-triA1** (m/z 1386.7991 for [M+H]+)

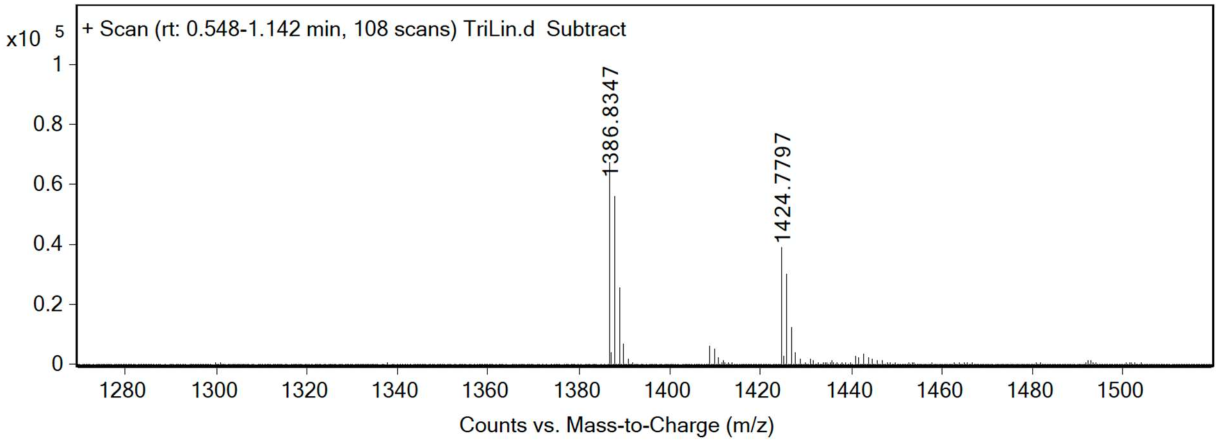

REFERENCES

(1) Jorgensen, W. L.; Chandrasekhar, J.; Madura, J. D.; Impey, R. W.; Klein, M. L. Comparison of Simple Potential Functions for Simulating Liquid Water. *J. Chem. Phys.* **1983**, *79* (2), 926–935. https://doi.org/10.1063/1.445869.

(2) Jo, S.; Kim, T.; Iyer, V. G.; Im, W. CHARMM-GUI: A Web-Based Graphical User Interface for CHARMM. *J. Comput. Chem.* **2008**, *29* (11), 1859–1865. https://doi.org/10.1002/jcc.20945.

(3) Huang, J.; Rauscher, S.; Nawrocki, G.; Ran, T.; Feig, M.; de Groot, B. L.; Grubmüller, H.; MacKerell, A. D. CHARMM36m: An Improved Force Field for Folded and Intrinsically Disordered Proteins. *Nat. Methods* **2017**, *14* (1), 71–73. https://doi.org/10.1038/nmeth.4067.

(4) Essmann, U.; Perera, L.; Berkowitz, M. L.; Darden, T.; Lee, H.; Pedersen, L. G. A Smooth Particle Mesh Ewald Method. *J. Chem. Phys.* **1995**, *103* (19), 8577–8593. https://doi.org/10.1063/1.470117.

(5) Feller, S. E.; Zhang, Y.; Pastor, R. W.; Brooks, B. R. Constant Pressure Molecular Dynamics Simulation: The Langevin Piston Method. *J. Chem. Phys.* **1995**, *103* (11), 4613–4621. https://doi.org/10.1063/1.470648.
